# Conformational ensembles for protein structure prediction

**DOI:** 10.1038/s41598-024-84066-z

**Published:** 2025-03-12

**Authors:** Jiaan Yang, Wen Xiang Cheng, Peng Zhang, Gang Wu, Si Tong Sheng, Junjie Yang, Suwen Zhao, Qiyue Hu, Wenxin Ji, Qiong Shi

**Affiliations:** 1 Shenzhen Institutes of Advanced Technology, Chinese Academy of Sciences, Shenzhen, 518055 Guangdong China; 2 Micro Biotech, Ltd., Shanghai, 200123 China; 3 School of Basic Medicine, Tongji Medical College, Huazhong University of Science and Technology, Wuhan, 430030 Hubei China; 4 HYK High-Throughput Biotechnology Institute, Shenzhen, 518057 Guangdong China; 5 Wuhan International Biohub Cooperation, Wuhan, 430075 Hubei China; 6 iHuman Institute, ShanghaiTech University, Shanghai, 201210 China; 7 Beyang Therapeutics Co. Ltd, Shanghai, 201210 China; 8 National Facility for Protein Science in Shanghai, Shanghai Advanced Research Institute, Chinese Academy of Sciences, Shanghai, 201210 China; 9 Laboratory of Aquatic Genomics, College of Life Sciences and Oceanography, Shenzhen University, Shenzhen, 518057 China; 10 Biomedical Engineering, Shenzhen University of Advanced Technology, Shenzhen, 518060 China

**Keywords:** Protein structure prediction, Protein conformation, Protein folding, Intrinsically disordered protein, Protein intrinsic disorder, AlphaFold, Computational biology and bioinformatics, Molecular biology, Structural biology

## Abstract

Acquisition of conformational ensembles for a protein is a challenging task, which is actually involving to the solution for protein folding problem and the study of intrinsically disordered protein. Despite AlphaFold with artificial intelligence acquired unprecedented accuracy to predict structures, its result is limited to a single state of conformation and it cannot provide multiple conformations to display protein intrinsic disorder. To overcome the barrier, a FiveFold approach was developed with a single sequence method. It applied the protein folding shape code (PFSC) uniformly to expose local folds of five amino acid residues, formed the protein folding variation matrix (PFVM) to reveal local folding variations along sequence, obtained a massive number of folding conformations in PFSC strings, and then an ensemble of multiple conformational protein structures is constructed. The P53_HUMAN as a well-known protein and LEF1_HUMAN and Q8GT36_SPIOL as typical disordered proteins are token as the benchmark to evaluate the predicted outcomes. The results demonstrated an effective algorithm and biological meaningful process well to predict protein multiple conformation structures.

## Introduction

A native protein has flexible conformations because of lower free-energy barriers between different folding states. Although AlphaFold and other AI machine learning-based methods can predict protein structures in single status with accuracy against 3D structural data from experimental measurements, it has the limitation to obtain multiple conformation structures^1,2^. A native protein structure should be fully represented by an ensemble of multiple conformations in vary degrees^3,4^. Essentially, to overcome the limitation is into the challenge involving the solution for protein folding problem. A protein may take various pathways folding into a stable state when it emerges from a ribosome, also it may repeatedly unfold and refold during its lifetime, or take an altered conformation under different conditions^5^. According to the Levinthal paradox, a protein can be folded into an astronomical number of structural conformations^6^.

Generally, a protein itself may well curve into a folded state, and also its conformation may be changed under different environments and interactions. Several forces drive a protein itself to fold into 3D structure^7,8^. First, the hydrogen bond is the primary force to form secondary fragment, such as α-helix and β-strand in protein structure. Second, the hydrophobic interactions make the residues tightly packed in protein, which particularly lead hydrophobic residues predominantly in the core while the polar residues usually trend on the surface. Third, the electrostatic interactions between amino acids make attracting or repelling residue contact. Fourth, the constraints of preferred dihedral angles of neighboring backbone bond influence the protein folding with space orientation. However, a protein structure is not static, the folded proteins are in constant motion, which is crucial for biological activity. In vitro and cell studies, the free-energy barriers, ΔG^+^, for folding conformation change is quite small, which is only on the order of ~ 5 kcal/mol^3,4,9^. So, it is not supervised that a protein has folding flexibility, and its conformation may be changed in various physiological conditions, such as solvent, temperature, ligand and protein interaction, etc.^10^. The protein always folds into a structure that is thermodynamically stable under physiological conditions^11^. It is apparently that protein conformation has flexibility which is able to fluctuate between different structural states by itself or under different conditions. From perspective view of intrinsically disordered proteins (IDP) or intrinsically disordered range (IDR), many proteins fail to form a stable structure for entire protein or partial ranges in structure, which may have impact on biological functions or diseases^12,13^.

The acquisition of protein structure from its amino acid sequence is a challenge in structural biology for longstanding. The protein structures are investigated by both experimental measurements and computational predictions. The experimental techniques required the protein structure state to exist for a long enough time scale for observing and detection while the computational dynamics simulations are generally limited to modelling events with very short timescales on the order of about a microsecond^14^. X-ray crystallography and Cryo-electron microscopy (Cryo-EM) are efficient methods to determine some of protein folding 3D structure^15^. Protein nuclear magnetic resonance (NMR) is the primary method to collect multiple folding states for relative small proteins in solvent^16^. So far, over 225,000 structures are available in the Protein Data Bank (PDB)^17^, which provided rich information for protein folding study. Also, other methods are also able to provide the information for protein folding, such as fluorescence spectroscopy^18^, circular dichroism spectroscopy^18^ and single-molecule force spectroscopy^19^ etc. However, the progress of experimental measurements for protein 3D structures cannot keep up with the pace of rapid increase number of protein sequences determined by genetic code. So, the computational approaches to predict protein structure according to amino acid sequence are important. Since 1994, the Critical Assessment of Techniques for Protein Structure Prediction (CASP) has organized and promoted the community for protein structure prediction, and the quality of methods has been assessed^20,21^. Over hundreds of computational prediction methods are developed, which are categorized into two categories. One is template-based modelling, which is based on homologous proteins to predict similar structures^22^. Another is template-free modelling, which is based on force fields to obtain protein structure with a minimum in Gibbs free energy^23–25^. Some effective computational methods have developed, such as I-TASSER (Iterative Threading ASSEmbly Refinement)^26^ with the detection of structure templates from the PDB,and HHpred^27^ with sequence searches for protein homology information. Recently, with deep learning in artificial intelligence (AI), AlphaFold approach, including AphaFold 2 and AlphaFold 3, successfully predicted protein structures with higher accuracy than any other methods^20,21,28^. AlphaFold first handled the residues as nodes and the connection of residues for protein structure. Then, with neural network system, it trained residue-to-residue and atom–atom using an internal confidence measure based on protein 3D structures from PDB for unknown structures. The protein structure was refined by evolutionarily related multiple sequence alignment (MSA). With iterating process, AlphaFold obtained protein structure with higher accuracy, and its database contained over 200 million of predicted protein 3D structures. Although AlphaFold well predicted protein structure in single state^29^, it has not delivered a comprehensive solution for the protein folding problem^30^. In other words, despite the advances in protein structure prediction recently, the goal to acquire complete ensemble of protein folding conformations has not been approached yet.

The shortcomings and limitations of AlphaFold approach have been revealed in several aspects^31–34^. First, the predicted result from AlphaFold only represents a single state of protein structure. So the result cannot fully embody a native protein structure with conformational heterogeneity. Second, although the deep learning neural network technology helped to solve the complex protein prediction problem, but it cannot directly provide understanding how the protein is folded in biophysical principles. Third, AlphaFold is not single sequence method, which heavily depends on multiple sequence alignment (MSA) to predict protein structure. So, its predicted result is lower confidence if the number of homologous sequences in database is less. Also, it is not able to specify the subtle changes in sequence, such as residue mutation or species differentiation how to impact the change of folding conformation. Although some models have been proposed, such as RoseTTAFold, ESMFold, OpenFold and EigenFold etc.^34^, the correlation with protein folding flexibility still does not have the solution^34^.

Here, a new approach, FiveFold, has been developed trying to answer the protein folding problem and to provide comprehensive complete conformations for protein 3D structure prediction. The FiveFold approach is a single sequence method which contains four modules. First, a set of 27 of protein folding shape code (PFSC) in alphabet letters is established to cover complete folding space for five amino acid residues, which greatly simplify the complex protein folding object^35^. For any given protein 3D structure, its conformation can be expressed by a PFSC string and a PDB-PFSC database is created. Second, a database is generated which contained contains all possible folding patterns in PFSC letter for any combination of five amino acid residues. Third, the protein folding variation matrix (PFVM)assembled all possible local folding variants in each column with PFSC letter along sequence in matrix, which directly displays the fluctuation of folding conformations for the entire protein^36^. Also, it well revealed how the protein folding features were related to the order of amino acids in sequence. With optimization of various combinations of PFSC letters, a massive number of PFSC strings can be produced from PFVM, which represent a massive number of conformations. Fourth, an ensemble of multiple conformation 3D structures are constructed by high-throughput homogenous conformation search screening PDB-PFSC database with using these PFSC strings for protein structure prediction. The FiveFold approach has at least three significant features for protein structure prediction. First, the information for complete folding conformations is held in PFVM. An astronomical number of conformations for each protein is able to be explicitly obtained from its PFVM, which provides an unambiguous answer to Levinthal paradox. Of course, a set of most probable conformations in a limited number can be acquired, and then an ensemble of protein 3D structures is able to be predicted. Second, the FiveFold approach is a single sequence method, which a PFVM depended on its amino acid sequence, i.e. different sequence has different folding variations and features. Thus, any residue mutation in sequence or same protein for differentiation species has different PFVM, and then has different conformational structures. Third, the FiveFold provided a biophysically understanding algorithm to predict an ensemble of multiple conformation protein structures. Therefore, the FiveFold offered a meaningful approach which can overcome the shortcomings and limitations of current computational protein structure prediction.

## Materials and methods

The FiveFold approach is based on all possible folding patterns for free connected five points as well as five amino acid residues in protein, and then the conformational ensembles for protein structures are predicted. The approach is composed of four modules, such as PFSC, 5AAPFSC database, PFVM and construction of ensemble of protein structures. Also, the software, web server and supporting database are already established.

### Four modules

#### PFSC

**Fig. 1:**
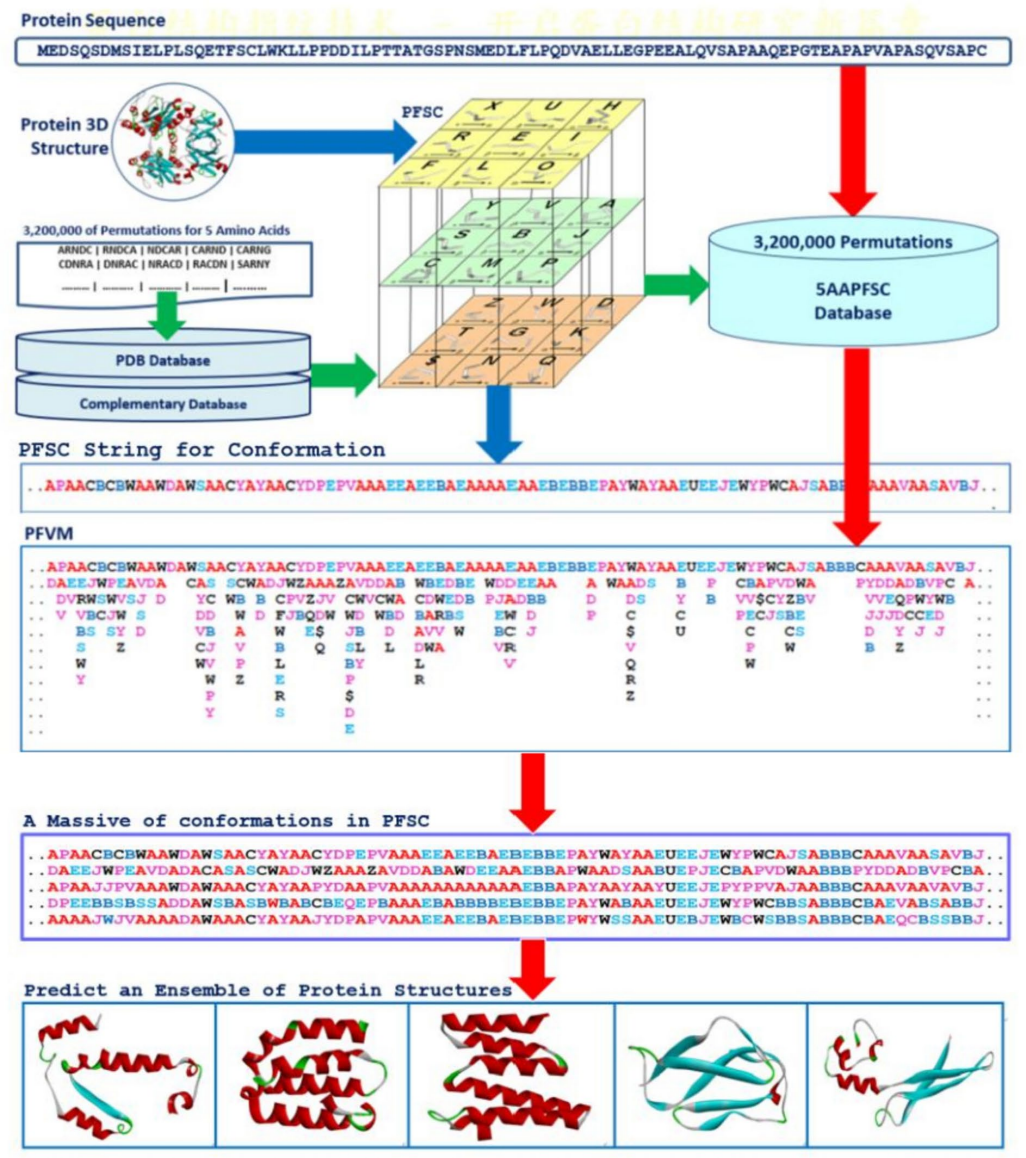
FiveFold approach to predict the conformational ensembles of protein structures with PFSC and PFVM algorithms. The cubic contains a set of 27 PFSC as folding patterns. The blue arrows indicated the process how to obtain the PFSC string from a protein with known 3D structure. The green arrows indicated the process to construct 5AAPFSC database, which contained all folding shapes in PFSC letters for 3,200,000 of permutations of five amino acids. The red arrows indicated the process to predict an ensemble of protein structures from a protein sequence with the PFVM. The PFSC letters with red and pink colors are for typical helix and alike-helix local folds; the blue and light blue colors for beta strand and alike-beta strand and block color for irregular folds^36^.

Any protein conformation is able to be completely described by a PFSC (protein folding shape code) alphabetic string. Mathematically, starting from a model of five points with successive connection without any constrain, a set of folding shapes for five of amino acid residues is obtained completely to cover folding space, which are represented by 27 letters inculding “$” as PFSC. The PFSC well character protein folding features, including alpha-helix, beta-strand, irregular folds and mixture in various degrees. For any protein with given 3D structure, its PFSC string is able fully to describe the folding conformation without gap along sequence, covering secondary structure fragments as well as tertiary structure fragments. The conversion procedure from a given protein 3D structure to PFSC is described by blue arrows in Fig. 1. Thus, a PFSC string, as the fingerprint of protein folding, corresponds to one protein conformation^35^. The conformations for all given structures in PDB were converted into PFSC string and stored into PDB-PFSC database.

#### 5AAPFSC

5AAPFSC database assembled all possible folding patterns for each five amino acid fragment in PFSC letters. Based on 20 amino acids, 3,200,000 permutations of five amino acids exist. All folding shapes for each set of five amino acids are collected from protein database or computed by MD (molecular dynamics) simulation. About two-thirds of permutations of five amino acids are available in PDB, so their folding patterns are able to be collected. The remnant one-third of permutations of five amino acids were computed by MD simulation with CHARMM (Chemistry at Harvard Macromolecular Mechanics)^37^. Then, all folding patterns for five amino acids are converted into the PFSC letters and stored in 5AAPFSC database. The procedure for database setup is described by green arrows in Fig. 1.

#### PFVM

The local folding variations for any protein can be fully presented by its protein folding variation matrix (PFVM), and the multiple conformations for entire proteins can be produced. Any set of five amino acid residues has different folding variants and different number of folding patterns, and these folding features are represented by PFSC letters which are extracted from 5AAPFSC database. For a protein, five amino acid residues are continuously taken along sequence and each step moves forward only one residue beginning from N-terminus. Extracting all possible folding shapes from 5AAPFSC database, the PFSC letters for each of five amino acids are ranked in a column according to favorability, and then PFVM is formed along sequence from N-terminus to C-terminus. The procedure forming PFVM is described by first two of red arrows in Fig. 1. To take a PFSC letter from each column in PFVM, an astronomical number of folding conformations in PFSC strings for a protein can be explicitly obtained without ambiguity. However, in practice a limit number of most possible folding conformations are interested to be obtained. The order of PFSC letters in each column are ranked from higher possibility to lower. So, the most possible local folds appeared on top in matrix. One of the most possible conformations is the PFSC string at the first row in PFVM that is named as PFVM-01, and more possible conformations can be obtained with PFVM-01 by replacing PFSC letter in the same column. In order to obtain a limited number of optimum conformations, the PFSC letters at top rows in PFVM are considered for replacement in PFVM-01. Thus, a massive number of protein folding conformations in PFSC strings can be formed by the local folding variations in PFVM, which is represented by third red arrow in Fig. 1. It is apparently that any protein sequence has its proprietary PFVM, which provided its special local folding variations to construct a massive of PFSC strings for protein conformations^36^.

#### Construction of ensemble of protein 3D structures

It is a critical step how to construct an ensembles of protein 3D structures from PFSC string. According to folding patterns from each PFSC string, its similar folding 3D protein structure can be found by high-throughput screening PDB-PFSC database with conformational homologous search. Because of digital description, the process of high-throughput screening with homologous conformation search is very effectively. Generally, a similar given 3D protein structure as the searched result is accepted if the normalized score of Protein Folding Structural Alignment (PFSA)^38^ is larger than 0.70. Due to larger protein is hard to meet the criteria, a protein is usually divided into multiple fragments with about 40–50 residues to satisfy the criteria, and then fragments are connected back for the whole protein. After that, the protein structure may be optimized by molecule dynamics simulation. Thus, the protein 3D structure is able to be constructed according each PFSC string, and the ensembles of protein 3D structures for multiple conformations are predicted according to multiple PFSC strings formed from its PFVM, which is presented by fourth red arrow in Fig. 1.

### Validation

The quantitative accuracy of FiveFold predicted protein structures is validated by comparison with given experimental structures. The protein structures are converted into the conformations with PFSC strings, and then the PFSC strings are aligned for comparison. A set of multiple conformational structures can be formed from its PFVM, which may cover wider range of flexible conformational space than given structures. If a protein has given structure, the predicted structures should have at least one conformation well to align with the given structure with 1.00 for the score of Protein Folding Structural Alignment (PFSA).

### Computational efficiency and feasibility

The computational overhead is low and the performance is efficient because the most routines in FiveFold are carried out based on digital alphabetic description for protein folding conformations. The software code was developed by Java (jdk1.8.0) and the database is stored in XML format. The task to predict conformational ensembles of protein structures was carried out on PC (i7-1355U, 1.70 GHz, 64-bit operating system) with Windows 11 platform. For example, the prediction of 10 conformational structures for a protein with 1000 amino acids in sequence takes a short period of time. Generally, from a sequence to obtain its PFVM takes about 5 s; from PFVM to form 10 PFSC strings takes about 10 s; the high-throughput homologous conformation search screening for 10 PFSC strings takes about 20 min; to construct 10 of protein 3D structures takes about 5 min. So, it takes less than 30 min to generate 10 conformational structures from its protein sequence.

## Results

The conformational ensembles of protein 3D structures are predicted by FiveFold approach with using PFVM and PFSC algorithm. Based on a sequence its specific PFVM is obtained, and then a massive number of PFSC strings is formed by combination of PFSC letters from PFVM, which represents the multiple folding conformations. According to these PFSC strings, an ensemble of 3D structures is constructed to characterize the most possible multiple conformations for a native protein. P53_HUMAN (P04637) is a structural well-known multiple domain protein with over two hundreds of given 3D structural data in PDB, and LEF1_HUMAN (Q9UJU2) and Q8GT36_SPIOL (Q8GT36) are two typical intrinsically disordered proteins. These proteins are good benchmarks to evaluate the predicted results of an ensemble of multiple conformation structures. Here, with FiveFold, an ensemble of multiple conformation structures are predicted for each protein, which are compared with given protein structures as well as AlphaFold predicted structure.

### P53_HUMAN

The P53_HUMAN (P04637) is composed of 393 amino acid residues which is a regulatory protein and prevents cancer formation. The P53_HUMAN is a protein with four domains. As a well-studied protein, the P53_HUMAN has about 260 of given 3D structural data in PDB, including over 140 for DNA-binding domain (100–288) and 3 for the entire protein. These structures provided rich folding information to evaluate the predicted results. Generally, the protein structure of a domain may swing around certain conformation while the fragments between domains and both terminuses have more folding flexibility. With FiveFold, the structure prediction will first focus on the DNA-binding domain, and then on the entire P53_HUMAN protein.

#### Domain structure prediction

**Fig. 2:**
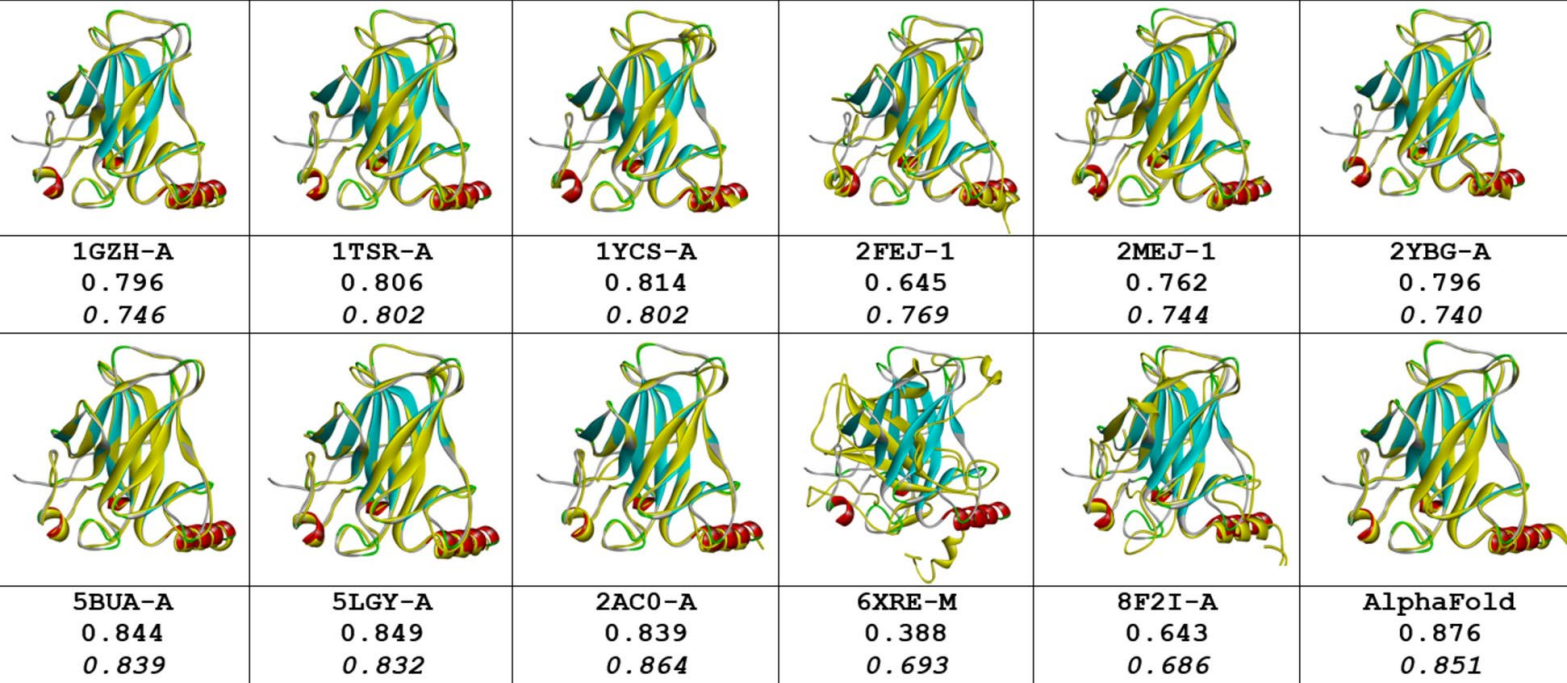
Pair comparisons between 2PCX-A structure and each given 3D structure of DNA-binding domain (100–288) of P53_HUMAN (P04637). The image of superposition of each pair of structures is displayed, the colorful structure is for 2PCX-A and yellow for the compared structure. The PDB-ID and chain name of the compared structure is listed under image. The overlap similarity score is displayed under PBD-ID, which was obtained by overlaying the molecule structures with alignment by a combination of 50% steric component and 50% electrostatic component in software of Discovery Studio v24.1.0. Also, with PFSC string alignment, the score of protein folding structural alignment (PFSA) in italics is listed in bottom.

As P53_HUMAN has many given 3D structures, it is important to understand the conformations of given 3D structures before protein structure prediction. Although a domain in protein may have a stable structure, the factors of environment and measurement methods indeed affect the folding conformation for a domain. The information about conditions, environment and measurement methods for 12 of P53 DNA-binding domain structures plus AlphaFold prediction are listed in Table 1. These protein structures were measured by different means crossing time span from 1995 to 2022. It is apparently that some of protein structures interact with ions, some with protein or DNA. So, it is not surprising that P53 DNA-binding domain may have different folding conformations in some degrees. Each structure is separately paired with 2PCX for comparison, and the 3D structural superposition and the overlay similarity scores are displayed in Fig. 2. Although the values of scores exposed which pair may have higher or lower similarity, but it is hard visually to distinguish or illustrate the difference between each pair of conformations. However, the PFSC is able well to reveal the conformation difference. Each conformation of given 3D structure is first expressed by a PFSC string. And each PFSC string is separately aligned with 2PCX in the C section of Table 2 for comparison. It obviously displayed the conformational similarity and dissimilarity, and the scores of protein folding structural alignment (PFSA) in italics are listed in Fig. 2. First, both similarity scores for most structures have parallel trend to evaluate the protein conformation similarity. Second, the PFSC alignment is better than the structure superposition to expose the local folding similarity and difference. The PFSC letters with red and pink colors are for typical helix and alike-helix local folds; the blue and light blue colors for beta strand and alike-beta strand. So, with quick scan on color of PFSC code, the secondary structure fragments are generally aligned while the difference of local folds are remarked by yellow color on background. Also, with using PFSC, the AlphaFold structure conformation (D section in Table 2) is able to be compared with given protein structures. Thus, the PFSC alignment better revealed the folding similarity and difference for given P53_HUMAN structures.

**Table 1:**
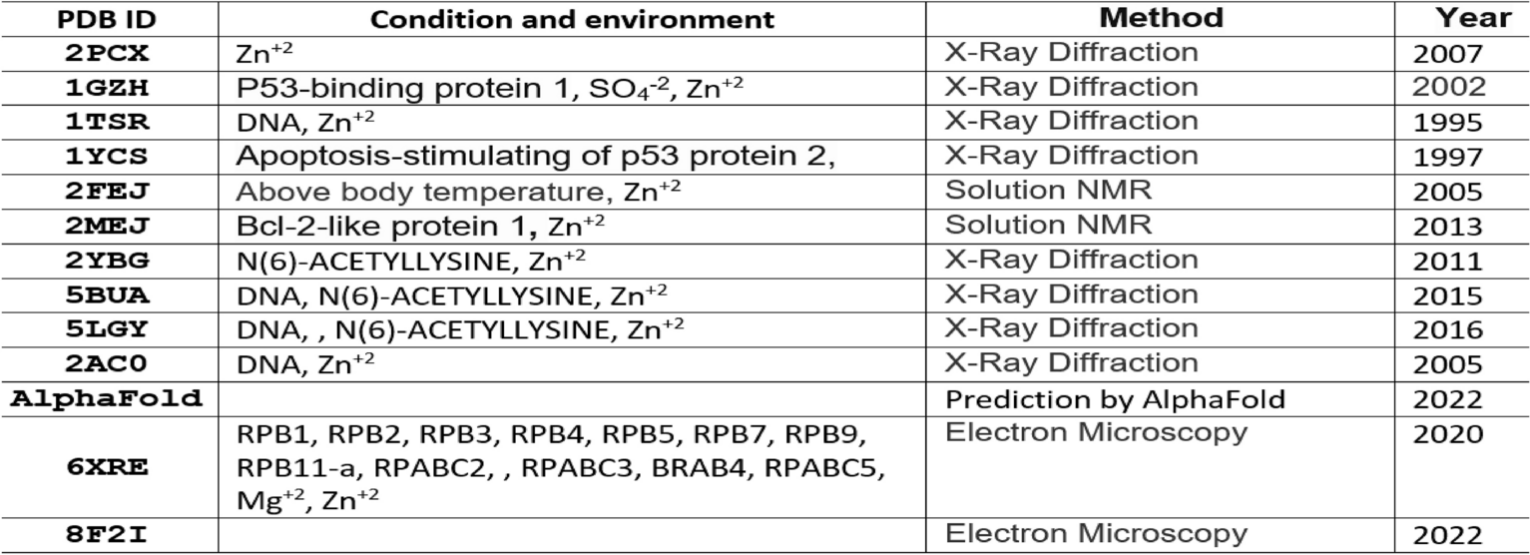
The different condition, environment and measurement method for 3D structures of DNA-binding domain regions (100–288) of P53_HUMAN (P04637) protein.

There are 13 protein structures timing span 1995–2022, which 8 were measured by X-Ray diffraction, 2 by solution NMR, 2 by electron microscopy in PDB and one from AlphaFold database. Also, the differences of condition and environment for each structure are listed.

**Table 2:**
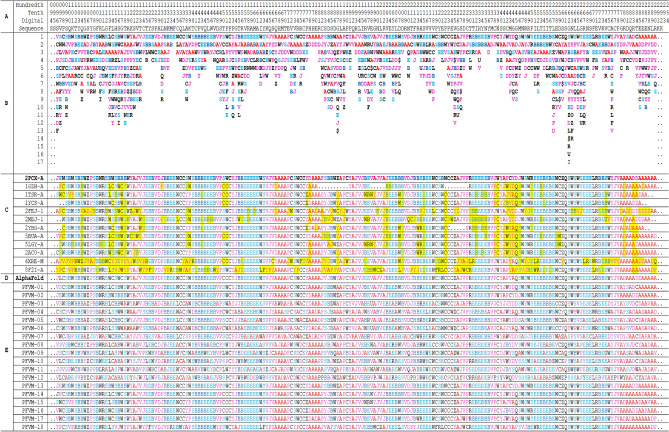
The PFVM and PFSC for DNA-binding domain region (100–288) of P53_HUMAN (P04637).

The protein sequence is listed in section A; the PFVM in section B; the conformations in PFSC string for given 3D structures in section C; the conformation of AlphaFold in PFSC string in section D and a set of conformations in PFSC string predicted by FiveFold in section E. The PFSC letters with red and pink colors are for typical helix and alike-helix local folds; the blue and light blue colors for beta strand and alike-beta strand and block color for irregular folds.

**Fig. 3:**
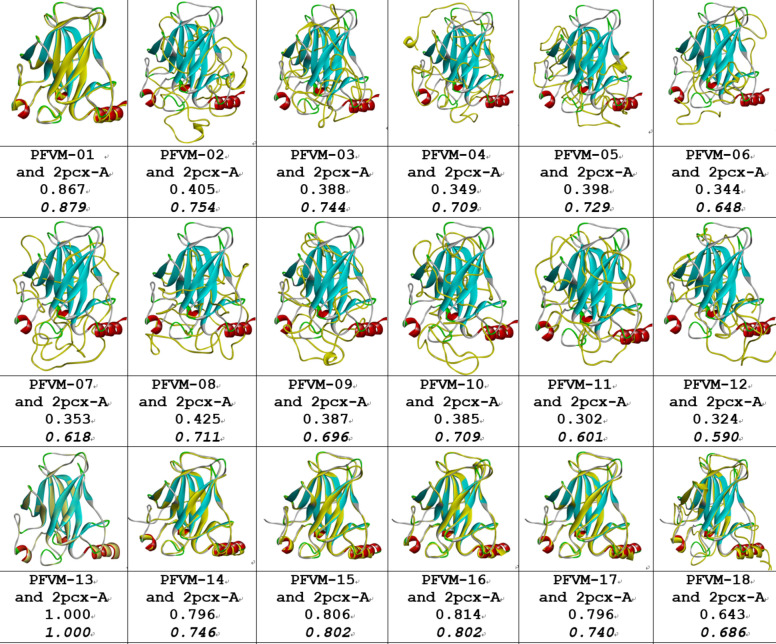
Comparisons between the given 2PCX-A structure and 18 of FiveFold predicted structure (yellow) for DNA-binding domain (100–288) of P53_HUMAN. The images of superposition of each pair of structures are displayed. The conformation name for FiveFold and given structure are listed under image. The overlap similarity score is displayed under structure name, which was obtained by overlaying the molecule structures with alignment by a combination of 50% steric component and 50% electrostatic component in software of Discovery Studio v24.1.0. Also, with PFSC string alignment, the score of protein folding structural alignment (PFSA) in italics is listed.

**Fig. 4:**
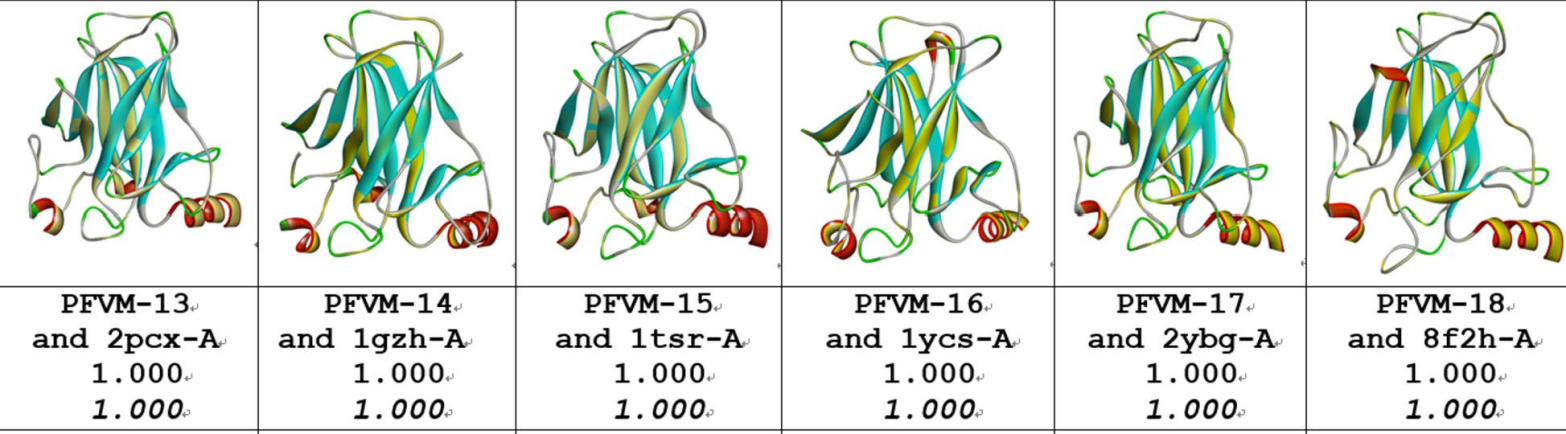
Comparisons between the FiveFold predicted structure (yellow) and each of 6 given structures for DNA-binding domain (100–288) of P53_HUMAN. The images of superposition of each pair of structures are displayed. The conformation name for FiveFold and given structure are listed under image. The overlap similarity score is displayed under structure name, which was obtained by overlaying the molecule structures with alignment by a combination of 50% steric component and 50% electrostatic component in software of Discovery Studio v24.1.0. Also, with PFSC string alignment, the score of protein folding structural alignment (PFSA) in italics is listed.

An ensemble of protein multiple conformation 3D structures for P53 DNA-binding domain is able to be constructed by FiveFold approach. The PFVM for DNA-binding domain regions (100–288) of P53_HUMAN (Section B of Table 2) is obtained according to its amino acid sequence in section A. The local folding variations of P53 are fluctuated in both folding patterns and numbers along sequence, which are explicitly displayed by PFSC at each column in PFVM. The first row in PFVM is a complete PFSC string, which is named as PFVM-01 representing one of most possible conformations. If any PFSC letter in PFVM-01 is replaced by a letter in same column, then total 1.33 × 10^150^ PFSC strings will be formed, i.e. astronomical number of folding conformations can be fashioned from PFVM. However, most biology researchers are interesting in a limit number of stable conformations. Based on PFVM-01, a set of most probable conformations with limit number is formed by the optimized process to replace the PFSC letters in same column in PFVM. For examples, with using PFSC letters in the second and third rows for replacement, a set of PFSC strings is formed. Also, with using PFSC vector feature, the optimized coupling PFSC string is able to be obtained. The section E of Table 2 listed 18 PFSC strings, which are some of possible multiple conformations of P53_HUMAN. It is obvious that 12 PFSC strings for given structures at the sections of C, a PFSC string from AlphaFold at the section D and 18 PFSC strings from FiveFold prediction at the section E in Table 2 are able to be aligned for comparison, and the local folding similarity and differences are exposed. Subsequently, 18 protein 3D structures can be constructed according to each PFSC string from FiveFold prediction with high-throughput screening PDB-PFSC database to search structures or fragments with conformation homology. Finally, 18 of protein 3D structures is able to be predicted. The superposition between each of the predicted protein 3D structure and the chain A of given P53_HUMAN protein structure 2PCX is displayed in Fig. 3, and the overlay similarity scores and the scores of PFSA in italics are listed. It is noticed that the values of overlay similarity scores in Fig. 3 for predicted structures are more dispersed than values in Fig. 2, which indicated the FiveFold predicted structures with wider flexibility than the given structures. Also, one conformation of the FiveFold predicted structures (PFVM-13) in Finger 3 is well matched with the given structure of 2PCX. It is not surprised that the PFVM contains the local folding variations which embrace all local folding patterns for given structures^36^. At the section C of Table 2, if the PFSC letter in each column for given structures is different from 2PCX, the background is remarked by yellow. In each column, the PFSC letter for 2PCX itself and other PFSC letters with yellow color are embraced by the PFSC letters of same column of PFVM in the section B. So, any conformation of protein given structure can be formed from PFVM. The Fig. 4 showed that each of 6 FiveFold predicted structures was well matched with its corresponding given structure. The results demonstrated that the FiveFold had a capability, which does not only can predict the multiple conformational structures in flexibility, but also can predict structure in accuracy to compare with given structure. Thus, an ensemble of multiple conformation protein P53 DNA-binding domain 3D structures with dispersed folding conformations is obtained while the structure with accuracy is simultaneously predicted.

#### Structure prediction for entire protein

**Fig. 5:**
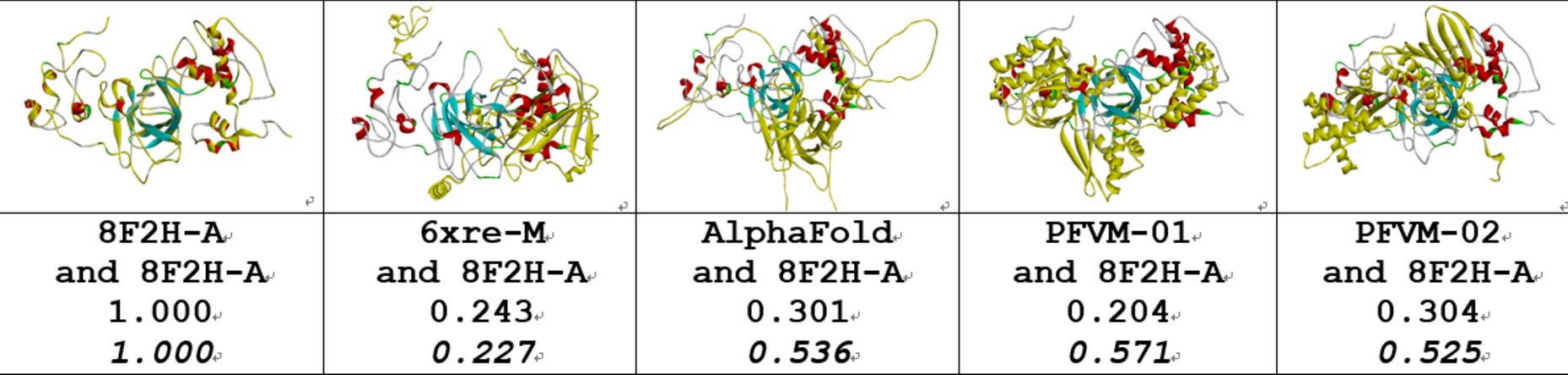
Pair comparisons between 8F2I-A and two given 3D structures, AlphaFold prediction and two FiveFold predicted structures for entire of P53_HUMAN protein. The image of superposition of each pair of structures is displayed, the colorful structure is for 8F2I-A and yellow color for compared structures. The structure names of the compared structures are listed under image. The overlap similarity score is displayed under structure name, which was obtained by overlaying the molecule structures with alignment by a combination of 50% steric component and 50% electrostatic component in software of Discovery Studio v24.1.0. Also, with PFSC string alignment, the score of protein folding structural alignment (PFSA) in italics is listed.

The 53 protein is composed of four domains, such as transactivation domain1 (6–30), transactivation domain 2 (35–59), DNA-binding domain (100–288) and tetramerisation domain (319–357). The ranges of these domains are marked by yellow color for sequence background in Table 3. The conformations of any domain may be fluctuated around a stable scaffold. As the P53_HUMAN has multiple domains in structure, higher disordered folds may happen at fragments between domains or N-terminal and C-terminal. For examples, the ranges between domains of 1–5, 31–34, 60–99 and 289–318 in PFVM do not have stable secondary structure fragment. Due to the 3D structure for entire P53_HUMAN protein being harder to obtain by experimental measurement or computational prediction, the limited number of structural data is available. Three given protein 3D structures (8F2I, 8F2H and 6XRE) are found in PDB which covered almost full range of P53_HUMAN protein, and a predicted structure in AlphaFold database. More of multiple conformations for P53_HUMAN structure can be obtained by FiveFold approach. The PFVM with full range of P53_HUMAN is displayed in the section under its sequence in Table 3. The PFSC conformations of given 3D structures and AlphaFold predicted structure as well as two FiveFold structural conformations (PFVM-01 and PFVM-02) formed from PFVM are listed at the bottom in Table 3. With PFSC colors, such as the red and pink for alpha helix, the blue and light blue for beta strand and black for irregular folding shape, it is apparently that the conformations of predicted P53 protein structures are overall aligned with given structures by secondary structure fragments. Two of FiveFold predicted 3D structures for P53 can be constructed by the PFSC strings (PFVM-01 and PFVM-02). Furthermore, the superposition between each 3D structure and 8F2I-A is visually displayed in Fig. 5. As multiple domains in P53 protein with several intrinsic disordered fragments, the structural difference in space orientation is not surprised. So, the structure superposition for protein comparison cannot obtain higher score. However, the PFSC alignment for protein comparison makes more sense because it avoids the difference in space orientation for protein fragments. With comparison of given structure of 8F2I-A , the overlap similarity score (with combination of 50% steric component and 50% electrostatic component) and the PFSA (folding structural alignment) score are listed under structures in Fig. 5. It is showed that the two FiveFold predicted structures have higher similar score of PFSA than AlphaFold structure, which indicated the similarity in conformation despite of space orientation difference. Therefore, it demonstrated the multiple conformational structures for entire P53_HUMAN protein with multiple domains can be predicted by FiveFold approach.

**Table 3:**
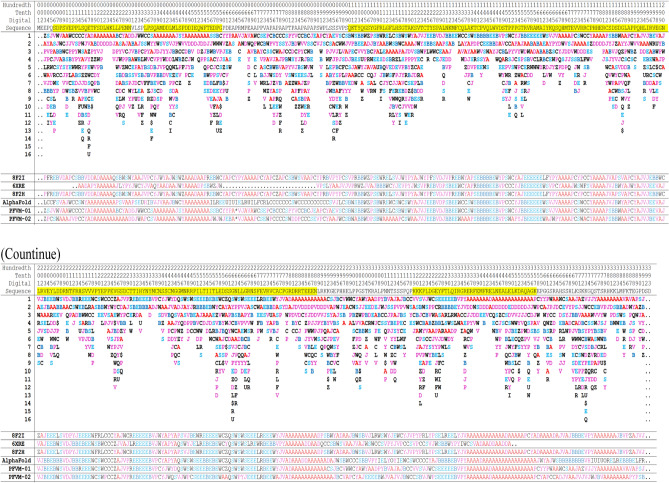
The PFVM and PFSC for entire P53_HUMAN.

The protein sequence is listed on top section; the PFVM on middle section and a set of conformations in PFSC strings for given structures, AlphaFold and FiveFold predicted structures on bottom section. The PFSC letters with red and pink colors are for typical helix and alike-helix local folds; the blue and light blue colors for beta strand and alike-beta strand and block color for irregular folds. The ranges of four domains are marked with yellow color in sequence.

### LEF1_HUMAN

**Fig. 6:**
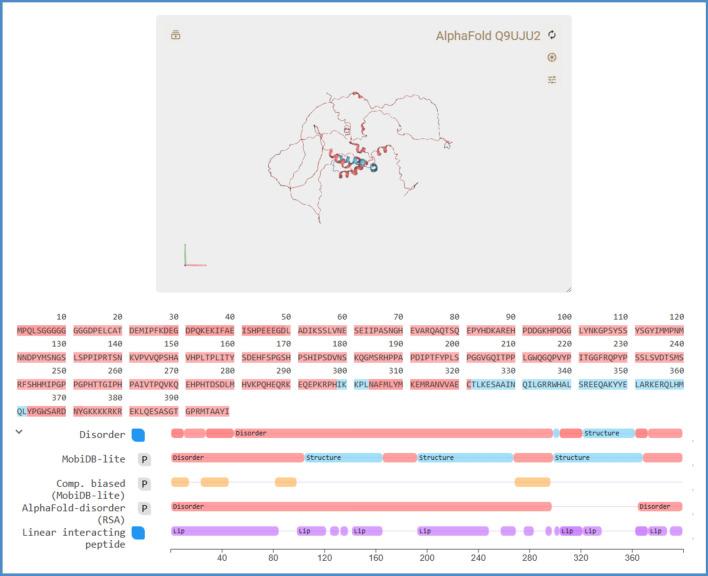
The intrinsically disordered information for LEF1_HUMAN protein from MobiDB. The 3D structure predicted by AlphaFold is displayed on top. The disorder ranges are marked by red color with various methods.

**Fig. 7:**
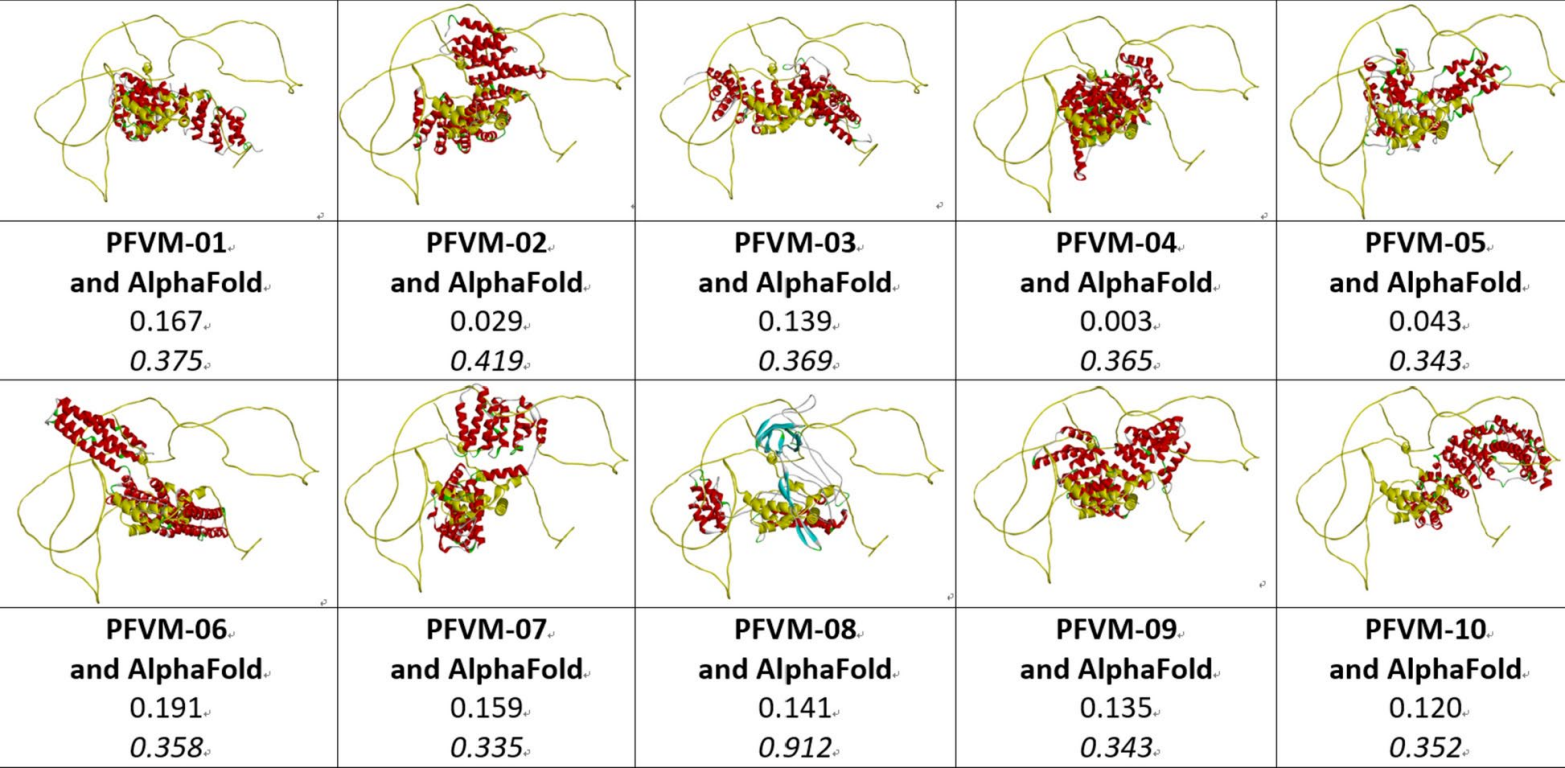
Structure superposition between AlphaFold structure and each of ten of FiveFold predicted structures. The image of AlphaFold structure is remarked by yellow color. Each of FiveFold predicted structure is constructed according its PFSC string in Table 4. The overlap similarity score is displayed under structure name, which was obtained by overlaying the molecule structures with alignment by a combination of 50% steric component and 50% electrostatic component in software of Discovery Studio v24.1.0. Also, with PFSC string alignment, the score of protein folding structural alignment (PFSA) in italics is listed.

The LEF1_HUMAN (Q9UJU2) is an intrinsically disordered protein, which is composed of 399 amino acid residues with two disorder regions, i.e. CTNNB1 binding domain (9–213) and high mobility group domain (298–368). The disordered information is available in database, such as the InterPro (integrated resource of protein families, domains and functional sites) or the protein disorder MobiDB^39,40^. The LEF1_HUMAN protein contains about 88% of disordered structure which is displayed in Fig. 6, it does not have any 3D structure data available in PDB, but a predicted structure can be obtained from AlphaFold database. Although, the AlphaFold offered a single state of 3D structural data, it indicated the LEF1_HUMAN as an intrinsically disordered protein. With FiveFold approach, the multiple conformational structures of LEF1_HUMAN can be predicted by its PFVM. The sequence of LEF1_HUMAN is listed in top section of Table 4, its PFVM is displayed in middle section, the conformations in PFSC for AlphaFold structure and 10 conformations obtained from PFVM are listed in bottom section. The folding similarity and dissimilarity are well exposed by alignment of these PFSC strings. It is obvious that the conformations formed from PFVM have more alpha-helical fragments than AlphaFold perdition. Also, most of the secondary structural fragments are aligned locally. According to each PFSC string formed from PFVM, its 3D structure can be constructed by FiveFold approach. Therefore, an ensemble of multiple conformation 3D structures for LEF1_HUMAN protein are able to be obtained. Each of ten of predicted structures predicted by FiveFold is directly compared with AlphaFold structure which is displayed in Fig. 7. The images apparently showed that most of structural superimpositions are widely dispersed while one of FiveFold structures (PFVM-08) has higher protein folding structural alignment (PFSA) score. It indicated higher similarity in folding conformation despite the difference of fragment space orientations. The results showed that the FiveFold is able to predict more of different conformation structures for the intrinsic disordered LEF1_HUMAN protein.

**Table 4:**
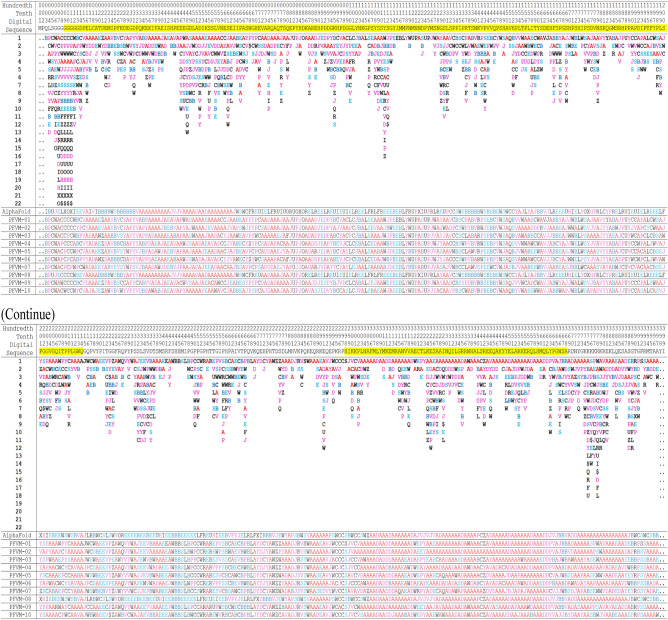
The PFVM and PFSC conformations for LEF1_HUMAN protein.

The protein sequence is listed on top section; the PFVM on middle section; a conformation of AlphaFold in PFSC string and ten of PFSC conformations formed from PFVM on bottom section. The PFSC letters with red and pink colors are for typical helix and alike-helix local folds; the blue and light blue colors for beta strand and alike-beta strand and block color for irregular folds.

### Q8GT36_SPIOL

**Fig. 8:**
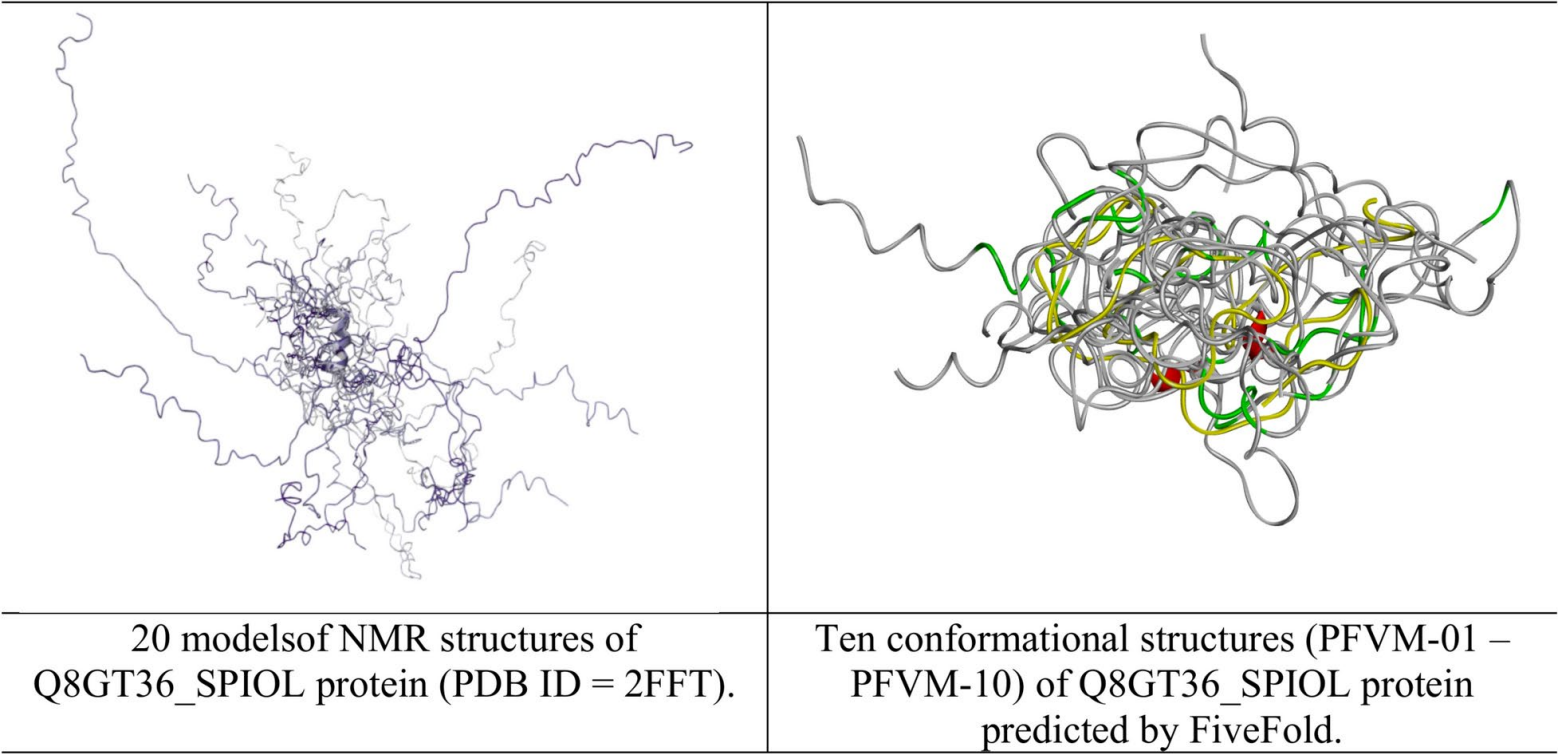
The images of superposition for NMR structures and superposition for an ensemble of multiple conformational structures predicted by FiveFold for Q8GT36_SPIOL protein.

The Q8GT36_SPIOL (Q8GT36) is a Spinach specific protein in the photosynthetic thylakoid membrane with 103 amino acids in sequence. This protein with TSP9 as its gene name is a characteristic sample to illustrate the intrinsically disordered protein with largely flexible conformations in NMR structures^41^. The PFVM of Q8GT36_SPIOL is obtained according to its sequence, then a set of possible conformations in PFSC is formed and an ensemble of 3D structures are predicted. The sequence of Q8GT36_SPIOL protein is displayed in section A in Table 5, its PFVM in section B and 22 possible conformations in PFSC string built from PFVM at section C while the conformations of 20 models of NMR structures (PDB ID = 2FFT) expressed by PFSC strings in section D. According the PFSC strings at section C, 10 conformational 3D structures are predicted and displayed in Fig. 8, which are compared with 20 models of NMR structures. With 3D structure comparison, it is obvious that both NMR structures and FiveFold predicted structures for the Q8GT36_SPIOL protein certainly exhibited flexible conformations. However, the visual observation is hard specifically to illustrate how the conformations are similar and different. Particularly, it is hard to compare more than two protein structures together. The PFSC alignment is easily to overcome these obstructs. With PFSC alignment of 22 FiveFold predicted conformations from PFVM in section C and 20 conformations of NMR structures in section D, the local folding similarity and dissimilarity are well uncovered. First, the conformation in range 35–43 has all alpha-helical folding features for both FiveFold predicted structures and NMR structures, and the same folding feature is also exposed at the first row PFSC in PFVM. Second, the conformation in range 78–82 of NMR structures have alpha-helical folding feature, but the FiveFold predicted structures from PFVM trend beta strand feature. Third, the conformations in other ranges certainly have various different folding features. These factors showed that the local folding similarity and difference for Q8GT36_SPIOL protein are well exposed by PFSC alignment. Thus, the FiveFold provided a useful tool to predict an ensemble of multiple conformational structures for intrinsically disordered proteins, the PFVM directly reveal the local folding variations and the PFSC alignment well expose the folding similarity and difference for all structures along sequence.

**Table 5:**
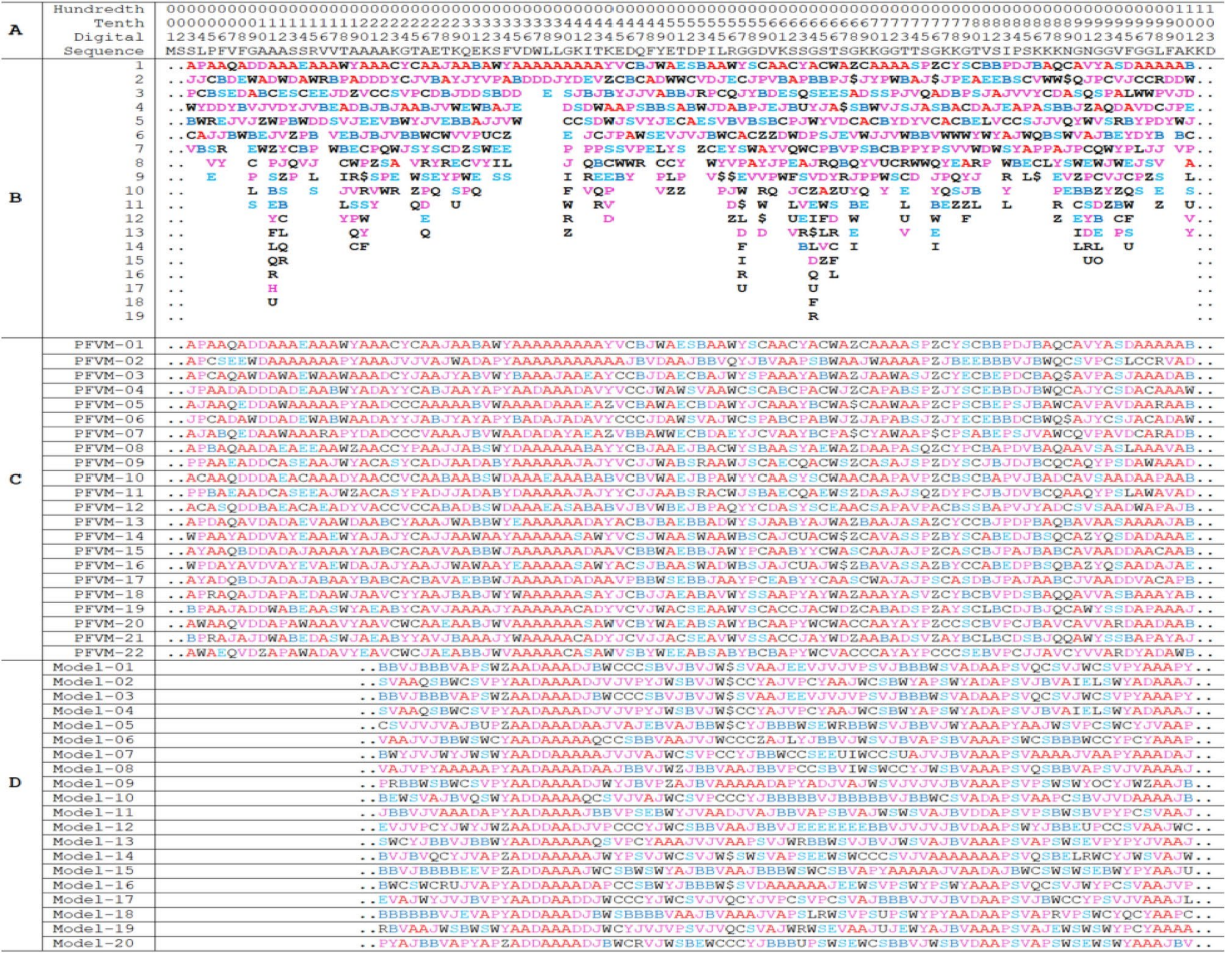
The PFVM and conformations in PFSC strings for Q8GT36_SPIOL protein.

The sequence is listed in section A; its PFVM in section B; 22 conformations in PFSC string built from PFVM in section C and 20 conformations of NMR structures (PDB ID = 2FFT) expressed in PFSC string in section D. The PFSC letters with red and pink colors are for typical helix and alike-helix local folds; the blue and light blue colors for beta-strand and alike-beta strand and block color for irregular folds.

## Discussion

The FiveFold approach in protein structure fingerprint technology is a single sequence method, which depends on the order of amino acids in a sequence to predict the conformational ensembles of protein structures. Any protein sequence has its exclusive PFVM which represents its local folding characteristic along sequence. Based on its PFVM, the protein multiple conformations are formed and represented by PFSC strings, and then an ensemble of multiple conformation structures can be constructed. Below discussion is about issues how the FiveFold may impact on the protein structure prediction.

### Accuracy vs. flexibility

The process of protein structure prediction is a computational approach to acquire three-dimensional structure according to its amino acid sequence. Many of molecular dynamics simulation approaches as well as AlphaFold deep learning-based system have achieved remarkable success with higher accuracy in the competition of CASP (Critical Assessment of Structure Prediction). It is indeed a big progress that the acquisition with high accuracy is archived for prediction of complex protein structure. However, the accuracy is only relative to a single state among protein flexible conformations. Most of native protein structures are conformation flexible or do not stay in a static state, i.e. it may fold and unfold itself in process for biological function, or alter the conformation as its environment and condition change. Thus, except the accuracy, the acquisition of multiple folding conformations for flexibility is another criterion to evaluate the results of protein structure prediction. Obviously, it is a pair of contradictions simultaneously to achieve both accuracy and flexibility. The protein folding process is guided by the interplay between residue interactions as well as the influence of environment, seeking to minimize the energy of the system and achieve a stable conformation or keep intrinsic disorder. The structure of a native protein is represented by the most energetically favorable state under physiological conditions. The consensus is that the order of amino acid in sequence determined its more possible folding conformations. And so, different proteins have different folding conformations while homology protein has similar folding conformation. The critical issue is how to predict protein structure with accuracy as well as how to obtain multiple conformations presenting flexibility. The FiveFold approach took a set of five amino acid residues as folding element, and all possible folds are described with PFSC alphabet digital letters. PFVM does not only well display the local folding fluctuations in pattern and number along sequence, but it is able exclusively to form an astronomical number of structural conformations for each individual sequence. Theoretically, the PFVM is able to provide comprehensive conformations against the protein folding problem. Practically, biological research is interesting to know a limited number of ensemble of folding conformation 3D structures. An ensemble of possible folding conformation 3D structures can be constructed by the optimized combinations using PFSC on top rows in PFVM with replacement of PFSC letters at same column. It is significant that the PFVM does not only construct the multiple conformations for flexible protein structures, but it also can predict the mutiple protein structures with high accuracy against each given structure. For accuracy, the PFVM contains all necessary PFSC letters which is able to form the PFSC strings matching exact conformations for all given structures measured by experimental approaches. Therefore, according to each of these matching PFSC strings, the FiveFold is able to construct 3D structures with accuracy separately to match each given structure. Thus, the FiveFold is a useful tool for protein multiple conformation structure prediction while both accuracy and flexibility are accommodated.

### Deep learning vs. meaningful biophysical algorithm

The protein structure prediction traditionally involved with biophysical base approaches, such as comparative modelling, fold recognition or energy minimization under force fields, but they are facing either result reliability or costing expensive computationally. Recently the AI deep learning methodology of artificial neural networks, such as AlphaFold and etc. successfully predicted the protein structures with remarkable accuracy and effectiveness. The AlphaFold trained on large amount protein data to discover the relationship between structural feature and data to identify statistical patterns, but it does not directly extract them into a format readily to be understood. Although AI deep neural networks are really good for solving the complex protein problems, it does not directly expose the terms of theory or model for biological scientists. Also, another problem is that the AI deep learning method depended on the amount of high-quality data. If training data is lack, the reliability of prediction would be lower.

The FiveFold approach adopted a more meaningful biophysical algorithm to predict protein structure. First, the five of amino acid residues has two consecutive dihedral angles which is the smallest folding element in protein backbone. Second, a set of 27 PFSC covered complete folding space for five point connection without constrain, but less than 27 PFSC are actually needed for five amino acid residues because of constrain. Third, each PFSC is a folding shape which is represented by a digital letter for simplification, which makes the possibility easily handling a massive number of complex protein folds. Fourth, any set of five amino acids have different number of various folding patterns but could not exceeding 27, which avoided the infinite folding images. Fifth, based on a sequence, its PFVM can be obtained, each column lists the folding shapes in PFSC for five amino acids which well represented the local folding fluctuations along sequence. Sixth, more important is that each protein has its specific PFVM, and a massive number of PFSC strings are able to be formed to represent multiple conformations. Finally, the multiple conformation protein 3D structures can be constructed based on PFSC strings with homologous conformation search, and then an ensemble of protein 3D structures are predicted for any protein. Thus, the meaningful biophysical algorithm is permeated into each step in FiveFold approach for protein structure prediction. The unique features and capabilities between AlphaFold and FiveFold can be compared. In summary, AlphaFold depended on multiple sequence alignment, but FiveFold adopted single sequence. The AlphaFold provided a protein structure in a single state and indicated the regions of intrinsic disorder, the FiveFold is able to provide a set conformational structures for protein multiple states and is able to provide various folding patterns for intrinsic disordered regions. The AlphaFold applied AI technology to predict the complex protein structures, the FiveFold adopted digital alphabetic description for five residues to simplify the complex protein folding problem.

### MSA vs. single sequence method

The multiple sequence alignment (MSA) is popularly applied by the AI deep learning process for protein structure prediction. It trains multiple sequences in protein database to obtain the predicted structure, so the results relied on plenty of homologous sequences^34^. Generally, it is true that the homologous sequences have similar folding conformation. However, the protein folding pattern is fundamentally determined by the order of amino acids in sequence. Thus, any difference in sequence may impact the protein folding conformation in various degrees. Especially it is significant to study protein mutation, to investigate the same protein for differentiation species or to design protein. Nevertheless, due to MSA application AlphaFold does not have the capability to distinguish the structural difference caused by few residue differences in mutation or species difference. In order avoid MSA process, some of single sequence methods, such as RGN2, ESMFold, OmegaFold and HelixFold-Single etc., have been developed with adoption of large language models (LLM) to predict protein structure. So far, however, it is still hard to reveal the structure difference caused by a few residue differences. Also, it does not provide knowledge for human-understandable form and the higher reliability in results. It is still a challenge to reveal the protein structural difference caused by slight variance in sequence, which is important to impact protein biological function, to understand the cause of diseases or to design new protein for biomedicine.

The FiveFold approach is a single sequence method, which is only based on its specific sequence to predict protein structure and is able to distinguish the structure difference caused by individual residue replacement. As different proteins have different sequences, they have different PFVM. Thus, although the same protein for different biological species is higher homologous, the conformational difference can be directly revealed by PFVM. Also, a single residue mutation in sequence will impact local fold patterns in five columns, which can be explicitly exposed in PFVM. The mutations in Hemoglobin subunit beta (HBB_HUMAN) protein caused different diseases, for example, the residue mutation of E7V caused Sickle cell disease and E27K caused Haemoglobin E. The residue mutations of E7V and E27K triggered the different local folding variations in PFVM, which are displayed in Table 6. The different local folding features are caused by mutations, and the folding change ranges are marked in red frames. With FiveFold, the 3D structures before and after mutation may be separately predicted by PFVM-01, which is the PFSC string on the first row of PFVM. It is obvious that the mutation E27K changed the residue physicochemical property from acidic to alkaline, and also the folding feature of typical alpha helix is deformed, which are marked in ovals in section A, B and C. The structure after mutation is colored with yellow in section C. It is obviously that the deformational helix around E27K twisted and caused the residue side chain to orientate different direction. Therefore, the PFVM is a significant single sequence approach with capability to reveal the folding difference in structure for both protein mutation and the same proteins of species differentiation.

**Table 6:**
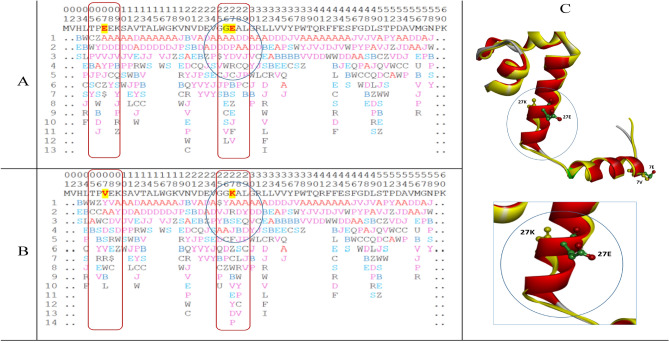
The conformation structure difference affected by mutations in Hemoglobin subunit beta (HBB_HUMAN) protein.

The PFVM before mutation is displayed in section A and the PFVM after mutation in section B. The differences of local folding features are marked in red rectangle and the folding features for E27K in oval. The 3D structures before and after mutation were predicted by FiveFold according to the PFSC string on the first row of PFVM, and superimposed and displayed in section C. The structure after mutation is yellow color, and the mutated residues are labelled around.

### Future development

It is significant to apply the FiveFold approach to predict protein structures and obtain multiple conformational structures. In PFVM, the protein local folding patterns are well represented by digital PFSC alphabetic letters in each column along sequence, which may benefit systematically to investigate the relationship between folding pattern changes and biological functions for complex proteins. However, there are many issues are needed to further advance and to associated with pharmaceutical and disease studies. First, a PFVM database may need to be generated for biological researchers to directly access. Also, the database can explicitly provide the folding patterns for study of intrinsically disordered protein. Second, with digital description, the PFVM may provide a useful means to explore the correlation between change of folding conformation and protein function or disease. It may become a systematical platform to associate with the cumulated rich experimental and clinical data. Third, to extract a limited number of optimum conformations from an astronomical number of possibilities of PFSC letter combinations from PFVM is a neural network process with multiple pathways, and hope an improved AI process to play better roles in future. Forth, the PFVM provided an effective platform with digital alphabetic letters presenting the comprehensive local folding shapes along protein sequence. It is significant to apply the PFVM to further explore an astronomical number of conformations for the protein folding problem as well as to provide concrete folding patterns for intrinsically disordered proteins.

## Supplementary Information

Supplementary Information 1.

Supplementary Information 2.

Supplementary Information 3.

Supplementary Information 4.

## Data Availability

The protein sequences in this study are available in UniProt database: P53_HUMAN: https://www.uniprot.org/uniprotkb/P04637/entry LEF1_HUMAN: https://www.uniprot.org/uniprotkb/Q9UJU2/entry Q8GT36_SPIOL: https://www.uniprot.org/uniprotkb/Q8GT36/entry The related protein 3D structures are available in PDB database: https://www.rcsb.org/structure/2PCXhttps://www.rcsb.org/structure/1GZHhttps://www.rcsb.org/structure/1TSRhttps://www.rcsb.org/structure/1YCShttps://www.rcsb.org/structure/2FEJhttps://www.rcsb.org/structure/2MEJhttps://www.rcsb.org/structure/5BUAhttps://www.rcsb.org/structure/5LGYhttps://www.rcsb.org/structure/2AC0https://www.rcsb.org/structure/6XREhttps://www.rcsb.org/structure/8F2I Alphafold Protein Structure Database: https://alphafold.com/search/text/P04637https://alphafold.com/search/text/Q9UJU2 The raw data in this study are available in supplementary files. Supplementary-01.rtf and supplementary-02.pdb are for P53_HUMAN protein; Supplementary-03.rtf for LEF1_HUMAN protein; Supplementary-04.rtf for Q8GT36_SPIOL protein.
