# Supplementary material for: Conformational ensembles for protein structure prediction
Source: Sci Rep. 2025 Mar 12;15:8513. doi: 10.1038/s41598-024-84066-z (PMC11904239; doi:10.1038/s41598-024-84066-z)
Supplement: Supplementary file 1 — Supplementary Information 1. [file 41598_2024_84066_MOESM1_ESM.rtf]

3D structure superimpose of 10 of given 3D structures from PDB  
                                                  


Sequence Alignment 
[DNA-binding domain regions(100-288)of P53_HUMAN]for 10 of given 3D structures from PDB 
1GZH	       SSVPSQKTYQGSYGFRLGFLHSGTAKSVTCTYSPALNKMFCQLAKTCPVQLWVDSTPPPGTRVRAMAIYKQSQHMTEVVRRCPHHERC......APPQHLIRVEG.LRVEYLDDRNTFRHSVVVPYEPP....ECTTIHYNYMCNSSCMGGMNRRPILTIITLEDSSGNLLGRNSFEVRVCACPGRDRRTEEENLRKK	
1TSR	      SSSVPSQKTYQGSYGFRLGFLHSGTAKSVTCTYSPALNKMFCQLAKTCPVQLWVDSTPPPGTRVRAMAIYKQSQHMTEVVRRCPHHERCSDSDGLAPPQHLIRVEGNLRVEYLDDRNTFRHSVVVPYEPPEVGSDCTTIHYNYMCNSSCMGGMNRRPILTIITLEDSSGNLLGRNSFEVRVCACPGRDRRTEEENL	
1YCS	         VPSQKTYQGSYGFRLGFLHSGTAKSVTCTYSPALNKMFCQLAKTCPVQLWVDSTPPPGTRVRAMAIYKQSQHMTEVVRRCPHHERCSDSDGLAPPQHLIRVEGNLRVEYLDDRNTFRHSVVVPYEPPEVGSDCTTIHYNYMCNSSCMGGMNRRPILTIITLEDSSGNLLGRNSFEVRVCACPGRDRRTEEE	
2FEJ	      SSSVPSQKTYQGSYGFRLGFLHSGTAKSVTCTYSPALNKMFCQLAKTCPVQLWVDSTPPPGTRVRAMAIYKQSQHMTEVVRRCPHHERCSDSDGLAPPQHLIRVEGNLRVEYLDDRNTFRHSVVVPYEPPEVGSDCTTIHYNYMCNSSCMGGMNRRPILTIITLEDSSGNLLGRNSFEVRVCACPGRDRRTEEENLRKKGEPHH	
2MEJ	        SVPSQKTYQGSYGFRLGFLHSGTAKSVTCTYSPALNKMFCQLAKTCPVQLWVDSTPPPGTRVRAMAIYKQSQHMTEVVRRCPHHERCSDSDGLAPPQHLIRVEGNLRVEYLDDRNTFRHSVVVPYEPPEVGSDCTTIHYNYMCNSSCMGGMNRRPILTIITLEDSSGNLLGRNSFEVRVCACPGRDRRTEEENLR	
2YBG	        SVPSQKTYQGSYGFRLGFLHSGTAxSVTCTYSPALNKMFCQLAKTCPVQLWVDSTPPPGTRVRAMAIYKQSQHMTEVVRRCPHHERCSDSDGLAPPQHLIRVEGNLRVEYLDDRNTFRHSVVVPYEPPEV.SDCTTIHYNYMCNSSCMGGMNRRPILTIITLEDSSGNLLGRNSFEVRVCACPGRDRRTEEEN	
5BUA	      SSSVPSQKTYQGSYGFRLGFLHSGTAxSVTCTYSPALNKMFCQLAKTCPVQLWVDSTPPPGTRVRAMAIYKQSQHMTEVVRRCPHHERCSDSDGLAPPQHLIRVEGNLRVEYLDDRNTFRHSVVVPYEPPEVGSDCTTIHYNYMCNSSCMGGMNRRPILTIITLEDSSGNLLGRNSFEVRVCACPGRDRRTEEENLRKKG	
5LGY	       SSVPSQKTYQGSYGFRLGFLHSGTAxSVTCTYSPALNKMFCQLAKTCPVQLWVDSTPPPGTRVRAMAIYKQSQHMTEVVRRCPHHERCSDSDGLAPPQHLIRVEGNLRVEYLDDRNTFRHSVVVPYEPPEVGSDCTTIHYNYMCNSSCMGGMNRRPILTIITLEDSSGNLLGRNSFEVRVCACPGRDRRTEEENLRK	
2AC0	      SSSVPSQKTYQGSYGFRLGFLHSGTAKSVTCTYSPALNKMFCQLAKTCPVQLWVDSTPPPGTRVRAMAIYKQSQHMTEVVRRCPHHERCSDSDGLAPPQHLIRVEGNLRVEYLDDRNTFRHSVVVPYEPPEVGSDCTTIHYNYMCNSSCMGGMNRRPILTIITLEDSSGNLLGRNSFEVRVCACPGRDRRTEEENLRKK	
2PCX	HHHHHHSSSVPSQKTYQGSYGFRLGFLHSGTAKSVTCTYSPALNKMFCQLAKTCPVQLWVDSTPPPGTRVRAMAIYKQSQHMTEVVRRCPHHERCSDSDGLAPPQHLIRVEGNLRVEYLDDRNTFRHSVVVPYEPPEVGSDCTTIHYNYMCNSSCMGGMNRRPILTIITLEDSSGNLLGRNSFEVRVCACPGRDQRTEEENLRKK	


PFSC (Protein Folding Shape Code) Alignment 
[DNA-binding domain regions(100-288)of P53_HUMAN]for 13 of given 3D structures from PDB and AlphaFold
	000000111111111111111111111111111111111111111111111111111111111111111111111111111111111111111111111111111122222222222222222222222222222222222222222222222222222222222222222222222222222222222222222222222222
999999000000000011111111112222222222333333333344444444445555555555666666666677777777778888888888999999999900000000001111111111222222222233333333334444444444555555555566666666667777777777888888888899999999
456789012345678901234567890123456789012345678901234567890123456789012345678901234567890123456789012345678901234567890123456789012345678901234567890123456789012345678901234567890123456789012345678901234567	
1GZH	 ..PCSBWSBBWZPSBWRELCSWCSWYAJVJEEBVDPSBEEWCCSWSBBBBEEBVPCCCYJBBEEEEEWSVJVDAAAPCSWCCYAAA..........YAJVJBE.....BBEBBBVDJBBEBEEWCSW........REEEEEBVPCYJWYDQSWSWSEEBBEBEWCZQSWSWSEEELRBEEWYJVADAAAAADAAAA..	
1TSR	..SWCSVPSBBWZPSBWRELSBBWSWYAJVJEEBVDJBBEEWCCYPSBBEEEEBVPCCCYJBBEEEEEWSVJVAAAAPCSWCCZAAAAPYJBWYAPCYAJVJWRWYJVJBEEBWSVDJBBEEEEWCSWCCCZAJVPREBEBBBVPCYAJVAQSWSWSEEBBEBEWCZQSWSWSEEELRBEBWYJVADAAAADAA..	
1YCS	   ..SBWSBEWZPSBWREWCSBWSWYAJVJEEBVDJBBEEWCCYPSBBBBEEBVPCCCYJBBEEEEEWSVJVAAAAPCSWCCZDAAAPYJBWYAPCYAJVJBEVAJVAJEEBWSVDJBBEEEEWCSWCCCZAJVPREEEEEBVPCYJBVDQSWSWREEEEEBEWCZQSWSWSEEELRBBBWYJVAAAADDA..	
2FEJ	..APCSBWSBVADJBBWRBWSEWCSWZJWYJEBBVDJBBEEWCCSWSBBBBEEBVPSWCYJBBEBEBEWSVJVDAAAJWSWCCYAADAAJVJWCYJWYAJVJEEVJBVJBEEBBBVDJBBVJEELCSWCCCYDJVPSBBEEEBVJWYAJVDDJWYJEEEEBEBEWCZQSBBVJEEELREEBWYJVDADDDDDDDDAAJWSVJ..	
2MEJ	  ..WSBWSBBWZPSBWREWSBBWSBVAJWSEEBVDPSBEELCCSWSBBEBBBBVPSWCYJWSEBEEEWSVJVAAAAPCSWCCYAAAAJBVJWZAJWYAJVJWRVAJVJBEEBBBVQCSBBBEEWCSWCSWZAJVPSBBBBBBVPCYAPYDDJWSWRBEBBEBEWCZQSWSWSEEELRBEUPYJVADAAADDDAA..	
2YBG	  ..CSBWSBBWZPSBWREWCSBWSWYAJWSEEBVDJBBEEWCCYPSBBEEEEBVPSWCYJBBEEEEEWSVJVAAAAPCSWCCZDADAPYJBWYAPCYAJVJBEVAJVJBEEBWSVDJBWREEEWCSWCC.....PRBBEBBBVPCYJWYAQSWSWSEEEBEBEWCZQSWSWSEEELRBBEWYJVADADAAAA..	
5BUA	..VPCSBWSBBWZPSBWRELCSBWSWCSWYJEEBVDJBBEEWCCSWSBBBBEEBVPCCCYJBBEEEEEWSVJVDAAAPCSWCCYDAAAJBBBWYAPCYAJVJBEVAJVJBBEBWSVDJBWREEEWCSWCCCZAJVPREBEEBBVPCYJWYDQSWSWREEBBEBEBWZQSWSWSEEELRBBEWYJVADAAAAAAAAADD..	
5LGY	 ..SWSBBBBBWZPSBBEELCSBWSWYAJVJEEBVDJBBEEWCCSWSBBEEEEBVPSWCYJBBEEEEEWSVJVDAAAPCSWCCYAAAAPYAJWZAPCYAJVJWRWYJVJBEEBBBVDJBWREEEWCSWCCCZAJVPREBEEEBVPCYJWYDQSWSWREEEBEBEBWZQSWSWSEEELRBBEWYJVADAAADAAAAA..	
2AC0	..CSWSBWSBBWZPSBWREWSBWCSWYJBVJEEBVDJBBEEWCCYPSBBBBEEBVPSWCYJBBEEEEEWSVJVDAAAPCSWCCYAAAAPYJBWYAPCYAJVJBEVAJVAJEEBWSVDJBWREEEWCSWCCCZAJVPREBEBEBVPCYJWYDQSWSWREEBBEBEWCZQSWSWSEEELRBBEWYJVADAAAAAAAAA...	
2PCX	BVJEWSBWSBEWZPSBWREBWSBBBWYAJVJEEBVDJBBEEWCCSWSBBBEEEBVPSWCYJBBEEEEEWSVJVAAAAPCSWCCYDAAAPSBBWZAPCYAJVJBEVAJVAJEEBBBVDJBBEBEEWCSBWCCZAJVPREEEEEBVJWYAPCYDJWSWREEEBEBEWCZQSWSWSEEELRBBEWYJVAAAADDAAAAAA..	
 6XRE  ..AJVJVPRWZJVAJBBBWCYJEVPYJWSVPREBVDPSBEEWCYAPRBBBBEEBVPCCCYJBEEEEEEWSVPCYAAAJWSWFSVAAAAJVJBWYAJWYAJVJEEVAJVAJEEBWSVDJBBEBEEWSBWCCCZJVAJLREEEEBVJWYAPYAQSWYJELREBEBEWCYQSWSWSLREWRBEBBVPYAAAAAADDAADQCCSBW..
 8F2I  ..YPCSVPRBBWZPSEWRELSVJWYPYAJWYPFSVDJVPREBWYAPSEBBBBEWYPSWCYAJEEEEEELFYPYAAAAPCYPCCYAAAAJVAPSVAPCYAJVJEBBWCZAJEEELSVDPYJEEEEWFRLCCCYAJWCREEEEEBVJWYAPYAPSVJBEWREEEEEWCZQSWSWSEEELREEEWYJVAAAAAAAAAADPSBWYA..
 AF    ..JLCSBWSBBWZPSBWREWCSWCSWYAJVJEEBVDPSBEEWCCSWSBBBBEEBVPCCCYJBBEEEEEWSVJVAAAAPCSWCCYAAAAJVJBWYAPCYAJVJEEVAJVJBBEBBBVDJBBEBEEWCSWCCCZAJVPREEEEEBVPCYAPYAQSWSWSEEBBEBEWCZQSWSWSEEELRBBEWYJVADAAADDAAAAAADAJW.


Folding difference between given 3D structure (Color marked difference from 1GZH), which revealed the local conformational difference
	000000111111111111111111111111111111111111111111111111111111111111111111111111111111111111111111111111111122222222222222222222222222222222222222222222222222222222222222222222222222222222222222222222222222
999999000000000011111111112222222222333333333344444444445555555555666666666677777777778888888888999999999900000000001111111111222222222233333333334444444444555555555566666666667777777777888888888899999999
456789012345678901234567890123456789012345678901234567890123456789012345678901234567890123456789012345678901234567890123456789012345678901234567890123456789012345678901234567890123456789012345678901234567
SSSVPSQKTYQGSYGFRLGFLHSGTAKSVTCTYSPALNKMFCQLAKTCPVQLWVDSTPPPGTRVRAMAIYKQSQHMTEVVRRCPHHERCSDSDGLAPPQHLIRVEGNLRVEYLDDRNTFRHSVVVPYEPPEVGSDCTTIHYNYMCNSSCMGGMNRRPILTIITLEDSSGNLLGRNSFEVRVCACPGRDQRTEEENLRKKGEPHH	
1GZH	 ..PCSBWSBBWZPSBWRELCSWCSWYAJVJEEBVDPSBEEWCCSWSBBBBEEBVPCCCYJBBEEEEEWSVJVDAAAPCSWCCYAAA..........YAJVJBE.....BBEBBBVDJBBEBEEWCSW........REEEEEBVPCYJWYDQSWSWSEEBBEBEWCZQSWSWSEEELRBEEWYJVADAAAAADAAAA..	
1TSR	..SWCSVPSBBWZPSBWRELSBBWSWYAJVJEEBVDJBBEEWCCYPSBBEEEEBVPCCCYJBBEEEEEWSVJVAAAAPCSWCCZAAAAPYJBWYAPCYAJVJWRWYJVJBEEBWSVDJBBEEEEWCSWCCCZAJVPREBEBBBVPCYAJVAQSWSWSEEBBEBEWCZQSWSWSEEELRBEBWYJVADAAAADAA..	
1YCS	   ..SBWSBEWZPSBWREWCSBWSWYAJVJEEBVDJBBEEWCCYPSBBBBEEBVPCCCYJBBEEEEEWSVJVAAAAPCSWCCZDAAAPYJBWYAPCYAJVJBEVAJVAJEEBWSVDJBBEEEEWCSWCCCZAJVPREEEEEBVPCYJBVDQSWSWREEEEEBEWCZQSWSWSEEELRBBBWYJVAAAADDA..	
2FEJ	..APCSBWSBVADJBBWRBWSEWCSWZJWYJEBBVDJBBEEWCCSWSBBBBEEBVPSWCYJBBEBEBEWSVJVDAAAJWSWCCYAADAAJVJWCYJWYAJVJEEVJBVJBEEBBBVDJBBVJEELCSWCCCYDJVPSBBEEEBVJWYAJVDDJWYJEEEEBEBEWCZQSBBVJEEELREEBWYJVDADDDDDDDDAAJWSVJ..	
2MEJ	  ..WSBWSBBWZPSBWREWSBBWSBVAJWSEEBVDPSBEELCCSWSBBEBBBBVPSWCYJWSEBEEEWSVJVAAAAPCSWCCYAAAAJBVJWZAJWYAJVJWRVAJVJBEEBBBVQCSBBBEEWCSWCSWZAJVPSBBBBBBVPCYAPYDDJWSWRBEBBEBEWCZQSWSWSEEELRBEUPYJVADAAADDDAA..	
2YBG	  ..CSBWSBBWZPSBWREWCSBWSWYAJWSEEBVDJBBEEWCCYPSBBEEEEBVPSWCYJBBEEEEEWSVJVAAAAPCSWCCZDADAPYJBWYAPCYAJVJBEVAJVJBEEBWSVDJBWREEEWCSWCC.....PRBBEBBBVPCYJWYAQSWSWSEEEBEBEWCZQSWSWSEEELRBBEWYJVADADAAAA..	
5BUA	..VPCSBWSBBWZPSBWRELCSBWSWCSWYJEEBVDJBBEEWCCSWSBBBBEEBVPCCCYJBBEEEEEWSVJVDAAAPCSWCCYDAAAJBBBWYAPCYAJVJBEVAJVJBBEBWSVDJBWREEEWCSWCCCZAJVPREBEEBBVPCYJWYDQSWSWREEBBEBEBWZQSWSWSEEELRBBEWYJVADAAAAAAAAADD..	
5LGY	 ..SWSBBBBBWZPSBBEELCSBWSWYAJVJEEBVDJBBEEWCCSWSBBEEEEBVPSWCYJBBEEEEEWSVJVDAAAPCSWCCYAAAAPYAJWZAPCYAJVJWRWYJVJBEEBBBVDJBWREEEWCSWCCCZAJVPREBEEEBVPCYJWYDQSWSWREEEBEBEBWZQSWSWSEEELRBBEWYJVADAAADAAAAA..	
2AC0	..CSWSBWSBBWZPSBWREWSBWCSWYJBVJEEBVDJBBEEWCCYPSBBBBEEBVPSWCYJBBEEEEEWSVJVDAAAPCSWCCYAAAAPYJBWYAPCYAJVJBEVAJVAJEEBWSVDJBWREEEWCSWCCCZAJVPREBEBEBVPCYJWYDQSWSWREEBBEBEWCZQSWSWSEEELRBBEWYJVADAAAAAAAAA...	
2PCX	BVJEWSBWSBEWZPSBWREBWSBBBWYAJVJEEBVDJBBEEWCCSWSBBBEEEBVPSWCYJBBEEEEEWSVJVAAAAPCSWCCYDAAAPSBBWZAPCYAJVJBEVAJVAJEEBBBVDJBBEBEEWCSBWCCZAJVPREEEEEBVJWYAPCYDJWSWREEEBEBEWCZQSWSWSEEELRBBEWYJVAAAADDAAAAAA..	
 6XRE  ..AJVJVPRWZJVAJBBBWCYJEVPYJWSVPREBVDPSBEEWCYAPRBBBBEEBVPCCCYJBEEEEEEWSVPCYAAAJWSWFSVAAAAJVJBWYAJWYAJVJEEVAJVAJEEBWSVDJBBEBEEWSBWCCCZJVAJLREEEEBVJWYAPYAQSWYJELREBEBEWCYQSWSWSLREWRBEBBVPYAAAAAADDAADQCCSBW..
 8F2I  ..YPCSVPRBBWZPSEWRELSVJWYPYAJWYPFSVDJVPREBWYAPSEBBBBEWYPSWCYAJEEEEEELFYPYAAAAPCYPCCYAAAAJVAPSVAPCYAJVJEBBWCZAJEEELSVDPYJEEEEWFRLCCCYAJWCREEEEEBVJWYAPYAPSVJBEWREEEEEWCZQSWSWSEEELREEEWYJVAAAAAAAAAADPSBWYA..
 AF    ..JLCSBWSBBWZPSBWREWCSWCSWYAJVJEEBVDPSBEEWCCSWSBBBBEEBVPCCCYJBBEEEEEWSVJVAAAAPCSWCCYAAAAJVJBWYAPCYAJVJEEVAJVJBBEBBBVDJBBEBEEWCSWCCCZAJVPREEEEEBVPCYAPYAQSWSWSEEBBEBEWCZQSWSWSEEELRBBEWYJVADAAADDAAAAAADAJW.


PFVM (Protein Folding Variation Matrix) for DNA-binding domain regions(100-288)of P53_HUMAN

Amino Acid Sequence:
     0000001111111111111111111111111111111111111111111111111111111111111111111111111111111111111111111111111111222222222222222222222222222222222222222222222222222222222222222222222222222222222222222222222
     9999990000000000111111111122222222223333333333444444444455555555556666666666777777777788888888889999999999000000000011111111112222222222333333333344444444445555555555666666666677777777778888888888999
     4567890123456789012345678901234567890123456789012345678901234567890123456789012345678901234567890123456789012345678901234567890123456789012345678901234567890123456789012345678901234567890123456789012
     SSSVPSQKTYQGSYGFRLGFLHSGTAKSVTCTYSPALNKMFCQLAKTCPVQLWVDSTPPPGTRVRAMAIYKQSQHMTEVVRRCPHHERCSDSDGLAPPQHLIRVEGNLRVEYLDDRNTFRHSVVVPYEPPEVGSDCTTIHYNYMCNSSCMGGMNRRPILTIITLEDSSGNLLGRNSFEVRVCACPGRDQRTEEENLRKK
PFVM:
   1 ..VSCSBWSBBWZPSBWRELCSBWSWYAJVJEEBVDJBBEEWCCSWSBBBBEEBVPSWCYJBBEEEEEWSVJVAAAAPCSWCCYAAAAPSBBWAAPCYAJVJBEVAJVJBEEBWSVDJBBREEEWCSWCCCZAJVPREBEEEBVPCYJWYDQSWSWSEEEBEBEWCZQSWSWSEEELRBEEWYJVAYSADCAAAAAA..
   2 ..CWWJVPBEEADJBYAABWSBWCAAAJWYSBBSAAPSAB LAYAPDPCEEBBDAVCCAPAJAABABAEAYPYDWVSJAAAASZDDDDJYJZAYYJWVVAAAWRUYBAEJBAABBAACSWEBLRASBBWYWYCDAJSBEBBBADJWAAJVADJAYJRBBBEBWYCAYAYPPVJASCWAEBBDASYDPJVAADDPVDD..
   3 ..JLVAPECVVYBCPALEAAAAPAJDVSBWPLWWCEAAJ  AVAYAAWVSWAYJCBLPYVYWDWADABABZSCYJDDVWDDDES   PABVJQZPWBZ DDEABWPWWAAAREEV QPADBWWCC EEVSAEWYPCERDA  D  SDVBQSVASVBEAJAAAEZVWAPBSBAPYB EESVUASP WAAJJWYJDDVV..
   4 ..PJSDJBESUPRWEESDSBRZLSPPPYEC R CEJEDD   WDJSRSSYA  WQARSJCSPSBVCLRBCEWPQSJJSSBLFWV   JSJYVJCSCSC  EBEWAJPRSELDD$  E J SARBJ  LPJV$VBB L  U     BZ AAJYQDPPBVCDJDSVBYWJCBVPVVW FB YAPB  YFCCVDZPJJPY..
   5 ..BCFCSJAWYJAVAUBQVEDEVPBYZCAE      BVP    ZDVPEEWS   EEPVSWPCERWWWDRDJYZPDPQ JW SB     WCASVDDB S  ZSLC$CAJVSDJJP  B   UJBSL    AJBDZY W  V      V PCPWZ DCCWW DLRBJBQYWCAEWSR PW SVBJ  C R  VPWWPJP..
   6 ..SPLPAARCC CQJ JBWSFPJYRBJDRA      Q      JPB  Y     WYWR ZWACDD   LVW  VY   ER J      QVWYCPWA    URCSCW EW  WWC  W   VPJDB    VSJPA            S DDYZY J  DP  WCWAJCDAADBCB  J   R C  P    YJYVWSJ..
   7 ..WBBVEDW A SAL CJYCJJAVCSEPLB      D       CJ         CJB A RWS    D    Z    DE R      YWPAPVQF    BDDAPS CB  BPL        YVE    WYPJV               JCA     LR    SEP$SVJCJBC        E        VBLE Q..
   8 ..AFYYY   W VRW FS FEREBDZ$BDD      S       E          JEF J SRJ              BJ         ACWBSJL    R VLE  BD  VLQ         WD     ZQY$               PQC      S    ASSP JVQQAJ        W        WCQC S..
   9 ..YE B    Z     I  VWWQRYJBESR      R       W          SYJ S EL                          PEC WVY    S  DY  SC  S            P      WQP                VS           CL$V PYYYYL        P        CSYY  ..
  10 ..ER W             JBVCJVVDW                             L B V                            SD Q Z       F                           DSQ                             RYV  ED DEP        R        S B   ..
  11 ..RY Z             RLYS WER                              E Q L                               E                                     RU                              J    ZO  LQ        L              ..
  12 ..DZ                Y I E                                                                    J                                     V                               P    DZ  UR        F              ..
  13 ..F                                                                                          $                                                                     D    LF            V              ..
  14 ..                                                                                                                                                                      $R                           ..
  15 ..                                                                                                                                                                      R                            ..
  16 ..                                                                                                                                                                      U                            ..
  17 ..                                                                                                                                                                      I                            ..
DonE!


PFVM contains all local folding shapes, which are able to construct the conformation for a given 3D sreucture
(All local folds for given 3D structure of 8F2I-A are covered in PFVM by green color)

             0000001111111111111111111111111111111111111111111111111111111111111111111111111111111111111111111111111111222222222222222222222222222222222222222222222222222222222222222222222222222222222222222222222
             9999990000000000111111111122222222223333333333444444444455555555556666666666777777777788888888889999999999000000000011111111112222222222333333333344444444445555555555666666666677777777778888888888999
             4567890123456789012345678901234567890123456789012345678901234567890123456789012345678901234567890123456789012345678901234567890123456789012345678901234567890123456789012345678901234567890123456789012
   Sequence: SSSVPSQKTYQGSYGFRLGFLHSGTAKSVTCTYSPALNKMFCQLAKTCPVQLWVDSTPPPGTRVRAMAIYKQSQHMTEVVRRCPHHERCSDSDGLAPPQHLIRVEGNLRVEYLDDRNTFRHSVVVPYEPPEVGSDCTTIHYNYMCNSSCMGGMNRRPILTIITLEDSSGNLLGRNSFEVRVCACPGRDQRTEEENLRKK
8F2I-A PFSC: ..JEWSBWSBEWZPSBWREBWSBBBWYAJVJEEBVDJBBEEWCCSWSBBBEEEBVPSWCYJBBEEEEEWSVJVAAAAPCSWCCYDAAAPSBBWZAPCYAJVJBEVAJVAJEEBBBVDJBBEBEEWCSBWCCZAJVPREEEEEBVJWYAPCYDJWSWREEEBEBEWCZQSWSWSEEELRBBEWYJVAAAADDAAAAAA..
        PFVM:
           1 ..VSCSBWSBBWZPSBWRELCSBWSWYAJVJEEBVDJBBEEWCCSWSBBBBEEBVPSWCYJBBEEEEEWSVJVAAAAPCSWCCYAAAAPSBBWAAPCYAJVJBEVAJVJBEEBWSVDJBBREEEWCSWCCCZAJVPREBEEEBVPCYJWYDQSWSWSEEEBEBEWCZQSWSWSEEELRBEEWYJVAYSADCAAAAAA..
           2 ..CWWJVPBEEADJBYAABWSBWCAAAJWYSBBSAAPSAB LAYAPDPCEEBBDAVCCAPAJAABABAEAYPYDWVSJAAAASZDDDDJYJZAYYJWVVAAAWRUYBAEJBAABBAACSWEBLRASBBWYWYCDAJSBEBBBADJWAAJVADJAYJRBBBEBWYCAYAYPPVJASCWAEBBDASYDPJVAADDPVDD..
           3 ..JLVAPECVVYBCPALEAAAAPAJDVSBWPLWWCEAAJ  AVAYAAWVSWAYJCBLPYVYWDWADABABZSCYJDDVWDDDES   PABVJQZPWBZ DDEABWPWWAAAREEV QPADBWWCC EEVSAEWYPCERDA  D  SDVBQSVASVBEAJAAAEZVWAPBSBAPYB EESVUASP WAAJJWYJDDVV..
           4 ..PJSDJBESUPRWEESDSBRZLSPPPYEC R CEJEDD   WDJSRSSYA  WQARSJCSPSBVCLRBCEWPQSJJSSBLFWV   JSJYVJCSCSC  EBEWAJPRSELDD$  E J SARBJ  LPJV$VBB L  U     BZ AAJYQDPPBVCDJDSVBYWJCBVPVVW FB YAPB  YFCCVDZPJJPY..
           5 ..BCFCSJAWYJAVAUBQVEDEVPBYZCAE      BVP    ZDVPEEWS   EEPVSWPCERWWWDRDJYZPDPQ JW SB     WCASVDDB S  ZSLC$CAJVSDJJP  B   UJBSL    AJBDZY W  V      V PCPWZ DCCWW DLRBJBQYWCAEWSR PW SVBJ  C R  VPWWPJP..
           6 ..SPLPAARCC CQJ JBWSFPJYRBJDRA      Q      JPB  Y     WYWR ZWACDD   LVW  VY   ER J      QVWYCPWA    URCSCW EW  WWC  W   VPJDB    VSJPA            S DDYZY J  DP  WCWAJCDAADBCB  J   R C  P    YJYVWSJ..
           7 ..WBBVEDW A SAL CJYCJJAVCSEPLB      D       CJ         CJB A RWS    D    Z    DE R      YWPAPVQF    BDDAPS CB  BPL        YVE    WYPJV               JCA     LR    SEP$SVJCJBC        E        VBLE Q..
           8 ..AFYYY   W VRW FS FEREBDZ$BDD      S       E          JEF J SRJ              BJ         ACWBSJL    R VLE  BD  VLQ         WD     ZQY$               PQC      S    ASSP JVQQAJ        W        WCQC S..
           9 ..YE B    Z     I  VWWQRYJBESR      R       W          SYJ S EL                          PEC WVY    S  DY  SC  S            P      WQP                VS           CL$V PYYYYL        P        CSYY  ..
          10 ..ER W             JBVCJVVDW                             L B V                            SD Q Z       F                           DSQ                             RYV  ED DEP        R        S B   ..
          11 ..RY Z             RLYS WER                              E Q L                               E                                     RU                              J    ZO  LQ        L              ..
          12 ..DZ                Y I E                                                                    J                                     V                               P    DZ  UR        F              ..
          13 ..F                                                                                          $                                                                     D    LF            V              ..
          14 ..                                                                                                                                                                      $R                           ..
          15 ..                                                                                                                                                                      R                            ..
          16 ..                                                                                                                                                                      U                            ..
          17 ..                                                                                                                                                                      I                            ..


Mutiple conformation search
1. A set of conformations in PFSC strings were generated from PFVM
2. Partition of each PFSC into small regions
3. Conformation searching for homolgy structures

PFVM-01
PFVM-01	Coupling	2-1 Mix	
5O1B	A	96	290	0.949	4IBT	A	96	290	0.787	5O1B	B	96	290	0.756	
5O1C	A	96	290	0.946	2AC0	A	96	290	0.774	5O1B	A	96	290	0.754	
6SI3	A	96	290	0.946	2AHI	B	96	290	0.774	5O1E	A	96	290	0.754	
5O1E	A	96	290	0.944	5O1B	A	96	290	0.772	5O1H	B	96	290	0.754	
5G4O	A	96	290	0.941	4IBU	A	96	290	0.769	6SI1	A	96	290	0.754	
6GGE	A	96	290	0.941	2J1Z	B	96	290	0.769	6SHZ	B	96	290	0.754	
2J1Z	B	96	290	0.941	5O1C	A	96	290	0.769	5AOL	A	96	290	0.751	
5O1D	A	96	290	0.941	5O1H	B	96	290	0.769	2J1W	B	96	290	0.751	
6SI2	A	96	290	0.941	6SI3	A	96	290	0.769	5O1C	A	96	290	0.751	
4AGM	A	96	290	0.938	4AGM	A	96	290	0.767	5O1D	A	96	290	0.751	
5AOM	A	96	290	0.938	3D0A	A	96	290	0.767	5O1D	B	96	290	0.751	
5G4N	A	96	290	0.938	3D0A	B	96	290	0.767	5O1G	A	96	290	0.751	
6GGB	A	96	290	0.938	5O1E	A	96	290	0.767	5A7B	B	96	290	0.751	
6GGC	A	96	290	0.938	5A7B	B	96	290	0.767	2AHI	B	96	290	0.751	
5O1F	A	96	290	0.938	6SHZ	B	96	290	0.767	5AOJ	B	96	290	0.751	
6SI1	A	96	290	0.938	5MCT	B	96	290	0.767	5AOK	B	96	290	0.751	
2BIM	A	96	290	0.936	5G4O	A	96	290	0.764	5O1E	B	96	290	0.751	
2J1W	B	96	290	0.936	6GGC	A	96	290	0.764	6SI1	B	96	290	0.751	
5O1A	A	96	290	0.936	5O1D	A	96	290	0.764	6SI3	B	96	290	0.751	
5O1G	A	96	290	0.936	5O1D	B	96	290	0.764	4XR8	D	96	290	0.751	
5O1I	A	96	290	0.936	6SI2	A	96	290	0.764	5MCW	B	96	290	0.751	


Coupling
Cut by 50 AA
	00000011111111111111111111111111111111111111111111 11111111111111111111111111111111111111111111111111 1111112222222222222222222222222222222222222222222 2222222222222222222222222222222222222222222222222222222
99999900000000001111111111222222222233333333334444 44444455555555556666666666777777777788888888889999 9999990000000000111111111122222222223333333333444 4444444555555555566666666667777777777888888888899999999
45678901234567890123456789012345678901234567890123 45678901234567890123456789012345678901234567890123 4567890123456789012345678901234567890123456789012 3456789012345678901234567890123456789012345678901234567
SSSVPSQKTYQGSYGFRLGFLHSGTAKSVTCTYSPALNKMFCQLAKTCPV QLWVDSTPPPGTRVRAMAIYKQSQHMTEVVRRCPHHERCSDSDGLAPPQH LIRVEGNLRVEYLDDRNTFRHSVVVPYEPPEVGSDCTTIHYNYMCNSSC MGGMNRRPILTIITLEDSSGNLLGRNSFEVRVCACPGRDQRTEEENLRKK	
Coupling	..VJWJBWABBWDPSBWAAAAAPCSWAAJVJEEBVDJBBEEWAAAPSBBB BEEBVPSWAPYJBEEEEEWAJWPYAAAPCSWACYAAAAPSBBWAAPCYAJ VJBEVAJVJBEEBWSVDJBBEEEEWCSWPCCZAJVPREBEEEBVPCYJW QSVJWPCSEEEBEBEWAAQSWPYJEEELRBEEWAJVAPSVDAAAAAAA..	
2-1 Mix	..VSWSBWBBBAZPBBWRBLCBBWAWYAWVJBEBADJBAEELCCAWSBCB BBEBAPSWAYJJBEBEEEESVPVAWAAPASWACYDAAAJSBZWAYPCYVJ VABEUAJVEBEABWBVDJSBRBEEACSWWCCYAJAPREEEEBBVJCYJJ YDDSWYWSEBEBBBECCZQYWSVSESELREEEDYJYAYSVDCDAAVAA.	

Add an Extra Residue for connection
Coupling	..VJWJBWABBWDPSBWAAAAAPCSWAAJVJEEBVDJBBEEWAAAPSBBB BEEBVPSWAPYJBEEEEEWAJWPYAAAPCSWACYAAAAPSBBWAAPCYAJ VJBEVAJVJBEEBWSVDJBBEEEEWCSWPCCZAJVPREBEEEBVPCYJW QSVJWPCSEEEBEBEWAAQSWPYJEEELRBEEWAJVAPSVDAAAAAAA..	

Coupling																				
94-143					144-193					194-243					244-297					
																				
4IBV	A	96	143	0.729	4IBU	B	143	193	0.824	2AHI	B	193	242	0.96	2PCX	A	242	290	0.724	
2AC0	A	96	143	0.719	5AB9	A	143	193	0.814	3KZ8	A	193	242	0.96	3VD1	I	262	310	0.704	
5MG7	A	96	143	0.719	5AOI	A	143	193	0.814	2AC0	C	193	242	0.95	3VD1	B	262	310	0.694	
5MG7	B	96	143	0.719	5AOM	A	143	193	0.814	7B4B	C	193	242	0.95	3VD1	C	262	310	0.694	


2-1Mix
	000000111111111111111 11111111111111111111111111111 111111111111111111111111 11111111111111111111111111 1111112222222222222222222222 222222222222222222222 22222222222222222222 22222222222222222222222222222222222
999999000000000011111 11111222222222233333333334444 444444555555555566666666 66777777777788888888889999 9999990000000000111111111122 222222223333333333444 44444445555555555666 66666667777777777888888888899999999
456789012345678901234 56789012345678901234567890123 456789012345678901234567 89012345678901234567890123 4567890123456789012345678901 234567890123456789012 34567890123456789012 34567890123456789012345678901234567
SSSVPSQKTYQGSYGFRLGFL HSGTAKSVTCTYSPALNKMFCQLAKTCPV QLWVDSTPPPGTRVRAMAIYKQSQ HMTEVVRRCPHHERCSDSDGLAPPQH LIRVEGNLRVEYLDDRNTFRHSVVVPYE PPEVGSDCTTIHYNYMCNSSC MGGMNRRPILTIITLEDSSG NLLGRNSFEVRVCACPGRDQRTEEENLRKK	
2-1 Mix	..VSWSBWBBBAZPBBWRBLC BBWAWYAWVJBEBADJBAEELCCAWSBCB BBEBAPSWAYJJBEBEEEESVPVA WAAPASWACYDAAAJSBZWAYPCYVJ VABEUAJVEBEABWBVDJSBRBEEACSW WCCYAJAPREEEEBBVJCYJJ YDDSWYWSEBEBBBECCZQY WSVSESELREEEDYJYAYSVDCDAAVAA..	
                              7EZJ : L :  114 :  132 : 0.763       |8DC4 : A :  115 :  143 : 0.741                           |1TSR : C :  144 :  167 : 0.833             |5BUA : A :  168 :  193 : 0.769                  |2BIO : A :  194 :  221 : 0.768                        |5JZR : A :   19 :   39 : 0.833         |7EZJ : b :  263 :  282 : 0.825     |2ARK : E :   82 :  109 : 0.750
94-143					144-193					194-243					244-297					
																				


2-2Mix
	000000111111111111111 11111111111111111111111111111 111111111111111111111111 11111111111111111111111111 1111112222222222222222222222 222222222222222222222 2222222222222222222222222222222222222222222222222222222
999999000000000011111 11111222222222233333333334444 444444555555555566666666 66777777777788888888889999 9999990000000000111111111122 222222223333333333444 44444445555555555666 66666667777777777888888888899999999
456789012345678901234 56789012345678901234567890123 456789012345678901234567 89012345678901234567890123 4567890123456789012345678901 234567890123456789012 34567890123456789012 34567890123456789012345678901234567
SSSVPSQKTYQGSYGFRLGFL HSGTAKSVTCTYSPALNKMFCQLAKTCPV QLWVDSTPPPGTRVRAMAIYKQSQ HMTEVVRRCPHHERCSDSDGLAPPQH LIRVEGNLRVEYLDDRNTFRHSVVVPYE PPEVGSDCTTIHYNYMCNSSC MGGMNRRPILTIITLEDSSG NLLGRNSFEVRVCACPGRDQRTEEENLRKK	
2-2 Mix	..CSCSVWSBEWDPSBARELS SWWSWAAJVSEBBVDPBBEEWACSWDBBB EEBBVPCWCYABAEEEBEWSYJYA AASPCSACSYAADAPSJBAAAPWYAJ AJWEVABVJBBEAWSVAJBBEELEWCBW CCWZCJVPSEBEBEAVPCAJW YAQJWSWREEEEEWEWCYQS WPWJEEEWRBEBWAJVAPSADAADAAAD..	
                              4XR8 : D :   96 :  114 : 0.816      |5A7B : A :  115 :  143 : 0.828                           |4A63 : A :  162 :  185 : 0.833             |5AB9 : A :  168 :  193 : 0.769                  |7B48 : D :  194 :  221 : 0.839                        |7EZJ : C :  242 :  262 : 0.786       |6KMM : A :  157 :  176 : 0.825 |4A63 : C :  283 :  310 : 0.786
94-143					144-193					194-243					244-297					
																				
 5AB9 	 B 	96	143	0.771	
 5AOI 	 B 	96	143	0.771	
 5AOM 	 B 	96	143	0.771	
 5LAP 	 B 	96	143	0.771	
 5O1A 	 B 	96	143	0.771	
 5O1C 	 B 	96	143	0.771	


2-3Mix
	000000111111111111111 11111111111111111111111111111 111111111111111111111111 11111111111111111111111111 1111112222222222222222222222 222222222222222222222 22222222222222222222 22222222222222222222222222222222222
999999000000000011111 11111222222222233333333334444 444444555555555566666666 66777777777788888888889999 9999990000000000111111111122 222222223333333333444 44444445555555555666 66666667777777777888888888899999999
456789012345678901234 56789012345678901234567890123 456789012345678901234567 89012345678901234567890123 4567890123456789012345678901 234567890123456789012 34567890123456789012 34567890123456789012345678901234567
SSSVPSQKTYQGSYGFRLGFL HSGTAKSVTCTYSPALNKMFCQLAKTCPV QLWVDSTPPPGTRVRAMAIYKQSQ HMTEVVRRCPHHERCSDSDGLAPPQH LIRVEGNLRVEYLDDRNTFRHSVVVPYE PPEVGSDCTTIHYNYMCNSSC MGGMNRRPILTIITLEDSSG NLLGRNSFEVRVCACPGRDQRTEEENLRKK	
2-3 Mix	..VWCSBPSBBAZJSBWAELC BBCSWYJJVJBESVDJSBEELCYSWSPBB BBEDVPSCCYJJBAEEEAWSVPVD AAAJCSWACZAAADPSBZWYAPCVAJ VABRVAJAJBEABBSVDCBBRBERWCSB CCCYADVPRBBEEBBDPCYAW YDDSASWSBEEBBBYWCZAS WSVSAEELABEEDYSVAYJADCDAPAAA..	
                               1KZY : B :   96 :  114 : 0.763      |2H1L : N :  115 :  143 : 0.810                          |2AC0 : B :  144 :  167 : 0.792             |4AGL : A :  168 :  193 : 0.865                   |3KMD : D :  194 :  221 : 0.804                      |6FF9 : D :  222 :  242 : 0.857     |3VD2 : C :  263 :  282 : 0.800   |2PCX : A :  263 :  290 : 0.750
94-143					144-193					194-243					244-297					
																				


2-4Mix
	00000011111111111111111111111111111111111111111111 11111111111111111111111111111111111111111111111111 1111112222222222222222222222222222222222222222222 2222222222222222222222222222222222222222222222222222222
99999900000000001111111111222222222233333333334444 44444455555555556666666666777777777788888888889999 9999990000000000111111111122222222223333333333444 4444444555555555566666666667777777777888888888899999999
45678901234567890123456789012345678901234567890123 45678901234567890123456789012345678901234567890123 4567890123456789012345678901234567890123456789012 3456789012345678901234567890123456789012345678901234567
SSSVPSQKTYQGSYGFRLGFLHSGTAKSVTCTYSPALNKMFCQLAKTCPV QLWVDSTPPPGTRVRAMAIYKQSQHMTEVVRRCPHHERCSDSDGLAPPQH LIRVEGNLRVEYLDDRNTFRHSVVVPYEPPEVGSDCTTIHYNYMCNSSC MGGMNRRPILTIITLEDSSGNLLGRNSFEVRVCACPGRDQRTEEENLRKK	
2-4 Mix	..CSWSVWBBEWDPBBARBLSSWWAWAAWVSEBBADPBAEEWACAWDBCB EEBBAPCWAYABAEBEBEESYJYAWASPASACSYDADAJSJBAAYPWYVJ AJWEUABVEBBEAWBVAJSBEELEACBWWCWZCJAPSEEEBEAVJCAJJ YAQJWYWREBEEEWECCYQYWPWJESEWREEBWAJYAPSVDAADAVAD..	

94-143					144-193					194-243					244-297					
																				


2-5Mix
	00000011111111111111111111111111111111111111111111 11111111111111111111111111111111111111111111111111 1111112222222222222222222222222222222222222222222 2222222222222222222222222222222222222222222222222222222
99999900000000001111111111222222222233333333334444 44444455555555556666666666777777777788888888889999 9999990000000000111111111122222222223333333333444 4444444555555555566666666667777777777888888888899999999
45678901234567890123456789012345678901234567890123 45678901234567890123456789012345678901234567890123 4567890123456789012345678901234567890123456789012 3456789012345678901234567890123456789012345678901234567
SSSVPSQKTYQGSYGFRLGFLHSGTAKSVTCTYSPALNKMFCQLAKTCPV QLWVDSTPPPGTRVRAMAIYKQSQHMTEVVRRCPHHERCSDSDGLAPPQH LIRVEGNLRVEYLDDRNTFRHSVVVPYEPPEVGSDCTTIHYNYMCNSSC MGGMNRRPILTIITLEDSSGNLLGRNSFEVRVCACPGRDQRTEEENLRKK	
2-5 Mix	..VWCJBPSEBAZJSYWAEWCBBCSAYJJYJBESVAJSBBELCYSPSPBE BBEDVVSCCPJJBAEAEAWAVPVDAVAJCAWACZADADPYBZWYAJCVAA VABRVYJAJJEABBSADCBWRBERWSSBCYCYADVJRBBBEBBDPWYAWVDDSASJSBEBBBBYWAZASPSVSAECLABBEDYSVDYJAACDAPADA..	

94-143					144-193					194-243					244-297					
																				
 1TSR 	 A 	96	143	0.667	
 1TUP 	 A 	96	143	0.667	
 4IBT 	 C 	96	143	0.667	


3-1Mix
	00000011111111111111111111111111111111111111111111 11111111111111111111111111111111111111111111111111 1111112222222222222222222222222222222222222222222 2222222222222222222222222222222222222222222222222222222
99999900000000001111111111222222222233333333334444 44444455555555556666666666777777777788888888889999 9999990000000000111111111122222222223333333333444 4444444555555555566666666667777777777888888888899999999
45678901234567890123456789012345678901234567890123 45678901234567890123456789012345678901234567890123 4567890123456789012345678901234567890123456789012 3456789012345678901234567890123456789012345678901234567
SSSVPSQKTYQGSYGFRLGFLHSGTAKSVTCTYSPALNKMFCQLAKTCPV QLWVDSTPPPGTRVRAMAIYKQSQHMTEVVRRCPHHERCSDSDGLAPPQH LIRVEGNLRVEYLDDRNTFRHSVVVPYEPPEVGSDCTTIHYNYMCNSSC MGGMNRRPILTIITLEDSSGNLLGRNSFEVRVCACPGRDQRTEEENLRKK	
3-1 Mix	..VSVSBWCBBYZPPBWRALCABWJWYABVJLEBCDJBJEEACCYWSBVB BAEBCPSWYYJWBEAEEEASVSVAJAAPWSWDCYAAAAASBJWAPPCYAJ VEBEWAJVABERBWVVDJABRWEECCSWVCCEAJPPREDEEEBVPCYJB YDVSWVWSEJEBABEVCZQBWSASEBELRSEEAYJVAYSJDCYAADAA.	

   1. .SWSBWSBBWZPSBWRELCSBWSWYAJVJEEBVDJBBEEWCCSWSBBB : 5AB9 : B :   96 :  143 : 0.771
   2. .SWSBWSBBWZPSBWRELCSBWSWYAJVJEEBVDJBBEEWCCSWSBBB : 5AOI : B :   96 :  143 : 0.771
   3. .SWSBWSBBWZPSBWRELCSBWSWYAJVJEEBVDJBBEEWCCSWSBBB : 5AOM : B :   96 :  143 : 0.771
   4. .SWSBWSBBWZPSBWRELCSBWSWYAJVJEEBVDJBBEEWCCSWSBBB : 5LAP : B :   96 :  143 : 0.771
   5. .SWSBWSBBWZPSBWRELCSBWSWYAJVJEEBVDJBBEEWCCSWSBBB : 5O1A : B :   96 :  143 : 0.771
   6. .SWSBWSBBWZPSBWRELCSBWSWYAJVJEEBVDJBBEEWCCSWSBBB : 5O1C : B :   96 :  143 : 0.771
   7. .SWSBWSBBWZPSBWRELCSBWSWYAJVJEEBVDJBBEEWCCSWSBBB : 5O1D : B :   96 :  143 : 0.771
   8. .SWSBWSBBWZPSBWRELCSBWSWYAJVJEEBVDJBBEEWCCSWSBBB : 5O1F : B :   96 :  143 : 0.771
   9. .SWSBWSBBWZPSBWRELCSBWSWYAJVJEEBVDJBBEEWCCSWSBBB : 5O1G : B :   96 :  143 : 0.771
  10. .SWSBWSBBWZPSBWRELCSBWSWYAJVJEEBVDJBBEEWCCSWSBBB : 5O1H : B :   96 :  143 : 0.771	   1. BEEBVPSWCYJBBEEEEEWSVJVAAAAPCSWCCYAAAAPSBBWYAPCYAJ : 5AB9 : A :  144 :  193 : 0.790
   2. BEEBVPSWCYJBBEEEEEWSVJVAAAAPCSWCCYAAAAPSBBWYAPCYAJ : 5AOI : A :  144 :  193 : 0.790
   3. BEEBVPSWCYJBBEEEEEWSVJVAAAAPCSWCCYAAAAPSBBWYAPCYAJ : 5AOM : A :  144 :  193 : 0.790
   4. BEEBVPSWCYJBBEEEEEWSVJVAAAAPCSWCCYAAAAPSBBWYAPCYAJ : 4LO9 : D :  144 :  193 : 0.790
   5. BEEBVPSWCYJBBEEEEEWSVJVAAAAPCSWCCYAAAAPSBBWYAPCYAJ : 4XR8 : C :  144 :  193 : 0.790
   6. BEEBVPSWCYJBBEEEEEWSVJVAAAAPCSWCCYAAAAPSBBWZAPCYAJ : 5AOI : B :  144 :  193 : 0.780
   7. BEEBVPSWCYJBBEEEEEWSVJVAAAAPCSWCCZAAAAPSBBWYAPCYAJ : 5AOK : A :  144 :  193 : 0.780
   8. BEEBVPSWCYJBBEEEEEWSVJVAAAAPCSWCCZAAAAPSBBWYAPCYAJ : 5AOL : A :  144 :  193 : 0.780
   9. BEEBVPSWCYJBBEEEEEWSVJVAAAAPCSWCCYDAAAPSBBWYAPCYAJ : 2GEQ : B :  141 :  190 : 0.780
  10. BEEBVPSWCYJBBEEEEEWSVJVAAAAPCSWCCYAAAAPSBBWZAPCYAJ : 4HJE : A :  144 :  193 : 0.780	   1. VJBEVAJVJBEEBWSVDJBBEBEEWCSWCCCZAJVPREBEEEBVPCYJB : 8DC4 : B :  194 :  242 : 0.776
   2. VJBEVAJVAJEEBWSVDJBBEEEEWCSWCCCZAJVPREEEEEBVPCYJB : 1YCS : A :  194 :  242 : 0.776
   3. VJBEVAJVAJEEBBBVDJBBEBEEWCSWCCCZAJVPREBEEEBVPCYJB : 2AHI : D :  194 :  242 : 0.765
   4. VJBEVAJVJBEEBWSVDJBBEEEEWCSWCCCZAJVPREBEBEBVPCYJB : 6FF9 : B :  194 :  242 : 0.765
   5. VJBEVAJVJBEEBWSVDJBWREEEWCSWCCCZAJVPREBEEEBVPCYJB : 4LO9 : D :  194 :  242 : 0.765
   6. VJBEVAJVJBEEBWSVDJBBEBEEWCSWCCCZAJVPREBEEEBVPCYJW : 2AHI : B :  194 :  242 : 0.755
   7. VJBEVAJVJBEEBWSVDJBWREEEWCSWCCCZAJVPREBEBEBVPCYJB : 7B46 : A :  194 :  242 : 0.755
   8. VJBEVAJVAJEEBBBVDJBBEBEEWCSWCCCZAJVPRBBEEEBVPCYJB : 8DC4 : A :  194 :  242 : 0.755
   9. VJBEVAJVJBEEBWSVDJBWREEEWCSWCCCZAJVPRBBEEEBVPCYJB : 5ECG : B :  194 :  242 : 0.755
  10. VJBBVAJVAJBEBBBVDJBBEWREWCSWCCCZAJVPREEEEEBVPCYJB : 4GUQ : B :  214 :  262 : 0.755	   1. YDQSWSWSEEEBEBEWCZQSWSWSEEELRBEEWYJVADAAAADAADAA : 3EXJ : A :  240 :  287 : 0.729
   2. YDQSWSWSEEBBEBEWCZQSWSWSEEELRBEEWYJVADAAAAAAADAA : 3EXL : A :  240 :  287 : 0.729
   3. YDQSWSWSEEEBEBEWCZQSWSWSEEELRBBEWYJVADAAAAAAAAAA : 4AGM : A :  243 :  290 : 0.719
   4. YDQSWSWSEEEBEBEWCZQSWSWSEEELRBBEWYJVADAAAAAAAAAA : 4AGN : A :  243 :  290 : 0.719
   5. YDQSWSWSEEEBEBEWCZQSWSWSEEELRBBEWYJVADAAADAAAAAA : 6GGC : A :  243 :  290 : 0.719
   6. YDQSWSWSEEEBEBEWCZQSWSWSEEELRBBEWYJVADAAADAAAAAA : 6GGE : A :  243 :  290 : 0.719
   7. YDQSWSWSEEBBEBEWCZQSWSWSEEELRBEEWYJVAAAAAAAAAAAD : 3KZ8 : A :  243 :  290 : 0.719
   8. YDQSWSWSEEBBEBEWCZQSWSWSEEELRBEEWYJVAAAAAAAAAAAD : 3KZ8 : B :  243 :  290 : 0.719
   9. YDQSWSWSEEEBEBEWCZQSWSWSEEELRBBEWYJVADAAAAAAAAAA : 5O1B : A :  243 :  290 : 0.719
  10. YDQSWSWSEEEBEBEWCZQSWSWSEEELRBBEWYJVADAAAAAAAAAA : 5O1C : A :  243 :  290 : 0.719	


3-2Mix
	00000011111111111111111111111111111111111111111111 11111111111111111111111111111111111111111111111111 1111112222222222222222222222222222222222222222222 2222222222222222222222222222222222222222222222222222222
99999900000000001111111111222222222233333333334444 44444455555555556666666666777777777788888888889999 9999990000000000111111111122222222223333333333444 4444444555555555566666666667777777777888888888899999999
45678901234567890123456789012345678901234567890123 45678901234567890123456789012345678901234567890123 4567890123456789012345678901234567890123456789012 3456789012345678901234567890123456789012345678901234567
SSSVPSQKTYQGSYGFRLGFLHSGTAKSVTCTYSPALNKMFCQLAKTCPV QLWVDSTPPPGTRVRAMAIYKQSQHMTEVVRRCPHHERCSDSDGLAPPQH LIRVEGNLRVEYLDDRNTFRHSVVVPYEPPEVGSDCTTIHYNYMCNSSC MGGMNRRPILTIITLEDSSGNLLGRNSFEVRVCACPGRDQRTEEENLRKK	
3-2 Mix	..JSCSPWSBVWBPSBLRELASPWSWVAJVPEWBVDABBEEWVCSWABBB WEYBVPLWCYYBDEEEAEWSZJCAAADPCSDCEYAAAAPSVBQAAPBYAJ DJAEVAWVJBAEEWSVQJBBBEWEWCEWCCAZWJVPEEBEEEDVPCDJW YSQAWSWEEEEAEEEWCAQSWBWPEEEERBEUWSJVAASADWAJAAAV..	

   1. JLCSBWSBBWZPSBWRELCSBWSWYAJVJEEBVDJBBEEWCCSWSBBB : 3Q05 : C :   96 :  143 : 0.771
   2. .CCSBWSBBWZPSBWRELCSBWSWYAJVJEEBVDJBBEEWCCSWSBBB : 6GGB : A :   96 :  143 : 0.760
   3. .CCSBWSBBWZPSBWRELCSBWSWYAJVJEEBVDJBBEEWCCSWSBBB : 2P52 : A :   93 :  140 : 0.760
   4. .CCSBWSBBWZPSBWRELCSBWSWYAJVJEEBVDJBBEEWCCSWSBBB : 6SI0 : A :   96 :  143 : 0.760
   5. CCCSBWSBBWZPSBWRELCSBWSWYAJVJEEBVDJBBEEWCCSWSBBB : 4XR8 : D :   96 :  143 : 0.760
   6. ..CSBWSBBWZPSBWRELCSBWSWYAJVJEEBVDJBBEEWCCSWSBBB : 5A7B : A :   96 :  143 : 0.750
   7. ..CSBWSBBWZPSBWRELCSBWSWYAJVJEEBVDJBBEEWCCSWSBBB : 5AB9 : A :   96 :  143 : 0.750
   8. .SWSBWSBBWZPSBWRELCSBWSWYAJVJEEBVDJBBEEWCCSWSBBB : 5AB9 : B :   96 :  143 : 0.750
   9. ..CSBWSBBWZPSBWRELCSBWSWYAJVJEEBVDJBBEEWCCSWSBBB : 5ABA : A :   96 :  143 : 0.750
  10. ..CSBWSBBWZPSBWRELCSBWSWYAJVJEEBVDJBBEEWCCSWSBBB : 4AGM : A :   96 :  143 : 0.750	   1. BEEBVPSWCYJBBEEEEEWSVJVAAAAPCSWCCYAAAAPSBBWYAPCYAJ : 5AB9 : A :  144 :  193 : 0.740
   2. BEEBVPSWCYJBBEEEEEWSVJVAAAAPCSWCCYAAAAPSBBWYAPCYAJ : 5AOI : A :  144 :  193 : 0.740
   3. BEEBVPSWCYJBBEEEEEWSVJVAAAAPCSWCCYAAAAPSBBWYAPCYAJ : 5AOM : A :  144 :  193 : 0.740
   4. BEEBVPSWCYJBEEEEEEWSVPYAAAAPCSWCCYAAAAPSBBWYAPCYAJ : 4KVP : D :  144 :  193 : 0.740
   5. BEEBVPSWCYJBBEEEEEWSVJVAAAAPCSWCCYAAAAPSBBWYAPCYAJ : 4LO9 : D :  144 :  193 : 0.740
   6. BEEBVPSWCYJBBEEEEEWSVJVAAAAPCSWCCYAAAAPSBBWYAPCYAJ : 4XR8 : C :  144 :  193 : 0.740
   7. BEEBVPSWCYJBBEEEEEWSVPYAAAAPCSWCCYAAAAPSBBWZAPCYAJ : 5ABA : B :  144 :  193 : 0.730
   8. BEEBVPSWCYJBBEEEEEWSVJVAAAAPCSWCCYAAAAPSBBWZAPCYAJ : 5AOI : B :  144 :  193 : 0.730
   9. BEEBVPSWCYJBBEEEEEWSVJVAAAAPCSWCCZAAAAPSBBWYAPCYAJ : 5AOK : A :  144 :  193 : 0.730
  10. BEEBVPSWCYJBBEEEEEWSVPYAAAAPCSWCCYAAAAPSVJWZAPCYAJ : 5AOK : B :  144 :  193 : 0.730	   1. VJBEVAJVJBBEBWSVDJBBEEEEWCSWCCCZAJVPREBEEEBVPCYJW : 3KZ8 : A :  194 :  242 : 0.755
   2. VJBEVAJVJBEEBWSVDJBBEBEEWCSWCCCZAJVPREBEEEBVPCYJW : 2AHI : B :  194 :  242 : 0.745
   3. VJBEVAJVJBBEBWSVDJBBEEEEWCSWCCCZAJVPREBEBEBVPCYJW : 6FJ5 : C :  194 :  242 : 0.745
   4. VJBEVAJVJBBEBWSVDJBBEEEEWCSWCCCZAJVPREBEBEBVPCYJW : 6FJ5 : D :  194 :  242 : 0.745
   5. VJEEVAJVJBBEBWSVDJBBEBEEWCSWCCCZAJVPREBEEEBVPCYJW : 3IGK : A :  194 :  242 : 0.745
   6. VJBEVAJVJBBEBWSVDJBBEEEEWCSWCCCZAJVPREBEEBBVPCYJW : 5MG7 : B :  194 :  242 : 0.745
   7. VJBEVAJVJBEEBWSVDJBBEEEEWCSWCCCZAJVPRBBEBEBVPCYJW : 2AC0 : C :  194 :  242 : 0.735
   8. VJBEVAJVJBBEBWSVDJBBEEEEWCSWCCCZAJVPREBEBBBVPCYJW : 7B49 : B :  194 :  242 : 0.735
   9. VJBEVAJVJBBEBWSVDJBBEEEEWCSWCCCZAJVPREBBBEBVPCYJW : 7B4A : B :  194 :  242 : 0.735
  10. VJBEVAJVJBEEBWSVDJBBEEEEWCSWCCCZAJVPREBEBBBVPCYJW : 7B4B : C :  194 :  242 : 0.735	   1. CYDJWSWREEEBEBEWCZQSWSWSEEELRBBEWYJVAAAADDAAAAAA : 2PCX : A :  243 :  290 : 0.771
   2. YDQSWSWREEEBEBEWCZQSWSWSEEEWRBBEWYJVAAAAAAAAAAAA : 4A63 : C :  263 :  310 : 0.740
   3. YDQSWSWREEEBEBEWCZQSWSWSEEELRBEEWYJVADAAAAAAAAA. : 2AC0 : B :  243 :  290 : 0.740
   4. YDQSWSWREEBBEBEWCZQSWSWSEEELRBBEWYJVADAAADAAAAAA : 2AC0 : D :  243 :  290 : 0.740
   5. YDQSWSWSEEEBEBEWCZQSWSWSEEELRBEEWYJVADAAADAAAAA. : 4AGO : A :  243 :  290 : 0.740
   6. YDQSWSWREEEBEBEWCZQSWSWSEEELRBBEWYJVADAAAAAAAAAA : 5AOL : A :  243 :  290 : 0.740
   7. YDQSWSWREEEBEBEWCZQSWSWSEEELRBBBWYJVAAAAADAAAAAD : 7B4D : A :  243 :  290 : 0.740
   8. YDQSWSWSEEEBEBEWCZQSWSWSEEELRBBEWYJVADAAADAAAAAA : 6GGC : A :  243 :  290 : 0.740
   9. YDQSWSWSEEEBEBEWCZQSWSWSEEELRBBEWYJVADAAADAAAAAA : 6GGE : A :  243 :  290 : 0.740
  10. YDQSWSWREEBBEBEWCZQSWSWSEEELRBBEWYJVADAAADAAAAAA : 5MCW : A :  243 :  290 : 0.740	

3-3Mix
	00000011111111111111111111111111111111111111111111 11111111111111111111111111111111111111111111111111 1111112222222222222222222222222222222222222222222 2222222222222222222222222222222222222222222222222222222
99999900000000001111111111222222222233333333334444 44444455555555556666666666777777777788888888889999 9999990000000000111111111122222222223333333333444 4444444555555555566666666667777777777888888888899999999
45678901234567890123456789012345678901234567890123 45678901234567890123456789012345678901234567890123 4567890123456789012345678901234567890123456789012 3456789012345678901234567890123456789012345678901234567
SSSVPSQKTYQGSYGFRLGFLHSGTAKSVTCTYSPALNKMFCQLAKTCPV QLWVDSTPPPGTRVRAMAIYKQSQHMTEVVRRCPHHERCSDSDGLAPPQH LIRVEGNLRVEYLDDRNTFRHSVVVPYEPPEVGSDCTTIHYNYMCNSSC MGGMNRRPILTIITLEDSSGNLLGRNSFEVRVCACPGRDQRTEEENLRKK	
3-3 Mix	..VLCSBESBBYZCSBWEELCABASWYSJVJLEWVDJABEEACASWSWBB BAEJVPSPCYJWBWEEEBWSVSVYAAAVCSWDCSAAAPPSBJWZAPCZAJ VEBBVAJWJBERBESVDPBBRWECWCSECCCEAYVPRRBEEEBVPCYVW YDVSSSWSAEEBABZWCZPSWSASYEELEBEEAYPVAYAADCYADAAA..	

   1. JLCSBWSBBWZPSBWRELCSBWSWYAJVJEEBVDJBBEEWCCSWSBBB : 3Q05 : C :   96 :  143 : 0.740
   2. ..CSBWSBBWZPSBWRELCSBWSWYAJVJEEBVDJBBEEWCCSWSBBB : 5A7B : A :   96 :  143 : 0.719
   3. ..CSBWSBBWZPSBWRELCSBWSWYAJVJEEBVDJBBEEWCCSWSBBB : 5AB9 : A :   96 :  143 : 0.719
   4. ..CSBWSBBWZPSBWRELCSBWSWYAJVJEEBVDJBBEEWCCSWSBBB : 5ABA : A :   96 :  143 : 0.719
   5. ..CSBWSBBWZPSBWRELCSBWSWYAJVJEEBVDJBBEEWCCSWSBBB : 4AGM : A :   96 :  143 : 0.719
   6. ..CSBWSBBWZPSBWRELCSBWSWYAJVJEEBVDJBBEEWCCSWSBBB : 4AGN : A :   96 :  143 : 0.719
   7. ..CSBWSBBWZPSBWRELCSBWSWYAJVJEEBVDJBBEEWCCSWSBBB : 4AGP : A :   96 :  143 : 0.719
   8. ..CSBWSBBWZPSBWRELCSBWSWYAJVJEEBVDJBBEEWCCSWSBBB : 4AGQ : A :   96 :  143 : 0.719
   9. ..CSBWSBBWZPSBWRELCSBWSWYAJVJEEBVDJBBEEWCCSWSBBB : 5AOI : A :   96 :  143 : 0.719
  10. ..CSBWSBBWZPSBWRELCSBWSWYAJVJEEBVDJBBEEWCCSWSBBB : 5AOJ : A :   96 :  143 : 0.719	   1. BEEBVPSWCYJBBEEEEEWSVJVAAAAPCSWCCYAAAAPSBBWZAPCYAJ : 5AOI : B :  144 :  193 : 0.800
   2. BEEBVPSWCYJBBEEEEEWSVJVAAAAPCSWCCYAAAAPSBBWZAPCYAJ : 4HJE : A :  144 :  193 : 0.800
   3. BEEBVPSWCYJBBEEEEEWSVJVAAAAPCSWCCYAAAAPSBBWZAPCYAJ : 2J1Z : B :  144 :  193 : 0.800
   4. BEEBVPSWCYJBBEEEEEWSVJVAAAAPCSWCCYAAAAPSBBWZAPCYAJ : 2J20 : B :  144 :  193 : 0.800
   5. BEEBVPSWCYJBBEEEEEWSVJVAAAAPCSWCCYAAAAPSBBWYAPCYAJ : 5AB9 : A :  144 :  193 : 0.790
   6. BEEBVPCCCYJBBEEEEEWSVJVDAAAPCSWCCYAAAAPSBBWZAPCYAJ : 2AC0 : B :  144 :  193 : 0.790
   7. BEEBVPSWCYJBBEEEEEWSVJVAAAAPCSWCCYDAAAPSBBWZAPCYAJ : 2ADY : A :  144 :  193 : 0.790
   8. BEEBVPSWCYJBBEEEEEWSVJVAAAAPCSWCCYAAAAPSBBWYAPCYAJ : 5AOI : A :  144 :  193 : 0.790
   9. BEEBVPSWCYJBBEEEEEWSVJVDAAAPCSWCCYAAAAPSVJWZAPCYAJ : 5AOL : B :  144 :  193 : 0.790
  10. BEEBVPSWCYJBBEEEEEWSVJVAAAAPCSWCCYAAAAPSBBWYAPCYAJ : 5AOM : A :  144 :  193 : 0.790	   1. VJBEVAJVJBEEBWSVDJBBEBEEWCSWCCCZAJVPREBEEEBVPCYJW : 2AHI : B :  194 :  242 : 0.776
   2. VJBEVAJVJBEEBBBVDJBBEBEEWCSWCCCZAJVPRBBEEEBVPCYJW : 2ATA : B :  194 :  242 : 0.765
   3. VJBEVAJVJBEEBBBVDJBBEBEEWCSWCCCZAJVPREBEBEBVPCYJW : 7B46 : D :  194 :  242 : 0.765
   4. VJBEVAJVJBEEBWSVDJBBEBEEWCSWCCCZAJVPRBBEEEBVPCYJW : 8DC4 : C :  194 :  242 : 0.765
   5. VJBEVAJVJBBEBBBVDJBBEWREWCSWCCCYAJVPRBBEEEBVPCYJW : 4GUO : K :  214 :  262 : 0.765
   6. VJBEVAJVJBEEBBBVDJBBEBEEWCSWCCCZAJVPREBEEEBVPCYAA : 2H1L : M :  194 :  242 : 0.765
   7. VJBEVAJVJBEEBWSVDJBBEBEEWCSWCCCZAJVPREEEEEBVPCYJW : 4HJE : B :  194 :  242 : 0.765
   8. VJBEVAJVJBBEBWSVDJBBEEEEWCSWCCCZAJVPREBEEEBVPCYJW : 3KZ8 : A :  194 :  242 : 0.765
   9. VJBEVAJVJBEEBWSVDJBBEEEEWCSWCCCZAJVPREBEEEBVPCYAJ : 2OCJ : C :  194 :  242 : 0.765
  10. VJBEVAJVJBEEBWSVDJBWREEEWCSWCCCZAJVPREBEEBBVPCYJW : 2AC0 : B :  194 :  242 : 0.755	   1. YDQSWSWSEEEBEBEWCZQSWSWSEEELRBBEWYJVADAAAAAAAAAA : 4AGM : A :  243 :  290 : 0.719
   2. YDQSWSWSEEEBEBEWCZQSWSWSEEELRBBEWYJVADAAAAAAAAAA : 4AGN : A :  243 :  290 : 0.719
   3. YDQSWSWSEEBBEBEWCZQSWSWSEEELRBEEWYJVADAAADDADAAA : 2GEQ : B :  240 :  287 : 0.719
   4. YDQSWSWSEEEBEBEWCZQSWSWSEEELRBBEWYJVADAAADAAAAAA : 6GGC : A :  243 :  290 : 0.719
   5. YDQSWSWSEEEBEBEWCZQSWSWSEEELRBBEWYJVADAAADAAAAAA : 6GGE : A :  243 :  290 : 0.719
   6. YDQSWSWSEEBBEBEWCZQSWSWSEEELRBEEWYJVAAAAAAAAAAAD : 3KZ8 : A :  243 :  290 : 0.719
   7. YDQSWSWSEEBBEBEWCZQSWSWSEEELRBEEWYJVAAAAAAAAAAAD : 3KZ8 : B :  243 :  290 : 0.719
   8. YDQSWSWSEEEBEBEWCZQSWSWSEEELRBBEWYJVADAAAAAAAAAA : 5O1B : A :  243 :  290 : 0.719
   9. YDQSWSWSEEEBEBEWCZQSWSWSEEELRBBEWYJVADAAAAAAAAAA : 5O1C : A :  243 :  290 : 0.719
  10. YDQSWSWSEEEBEBEWCZQSWSWSEEELRBBEWYJVADAAAAAAAAAA : 5O1D : A :  243 :  290 : 0.719	


3-4Mix
	00000011111111111111111111111111111111111111111111 11111111111111111111111111111111111111111111111111 1111112222222222222222222222222222222222222222222 2222222222222222222222222222222222222222222222222222222
99999900000000001111111111222222222233333333334444 44444455555555556666666666777777777788888888889999 9999990000000000111111111122222222223333333333444 4444444555555555566666666667777777777888888888899999999
45678901234567890123456789012345678901234567890123 45678901234567890123456789012345678901234567890123 4567890123456789012345678901234567890123456789012 3456789012345678901234567890123456789012345678901234567
SSSVPSQKTYQGSYGFRLGFLHSGTAKSVTCTYSPALNKMFCQLAKTCPV QLWVDSTPPPGTRVRAMAIYKQSQHMTEVVRRCPHHERCSDSDGLAPPQH LIRVEGNLRVEYLDDRNTFRHSVVVPYEPPEVGSDCTTIHYNYMCNSSC MGGMNRRPILTIITLEDSSGNLLGRNSFEVRVCACPGRDQRTEEENLRKK	
3-4 Mix	..JSVSPWCBVWBPPBLRALASPWJWVABVPEWBCDABJEEWVCYWABVB WEYBCPLWYYYBDEAEAEASZJCAJADPWSDCEYAAAAASVBQAPPBYAJ DJAEWAWVABAEEWVVQJABBEWECCEWVCAZWJPPEEDEEEDVPCDJB YSQAWVWEEJEAEEEVCAQBWBWPEBEERSEUWSJVAASJDWAJADAV..	

   1. .SWSBWSBBWZPSBWRELCSBWSWYAJVJEEBVDJBBEEWCCSWSBBB : 5AB9 : B :   96 :  143 : 0.635
   2. .SWSBWSBBWZPSBWRELCSBWSWYAJVJEEBVDJBBEEWCCSWSBBB : 5AOI : B :   96 :  143 : 0.635
   3. .SWSBWSBBWZPSBWRELCSBWSWYAJVJEEBVDJBBEEWCCSWSBBB : 5AOM : B :   96 :  143 : 0.635
   4. .SWSBWSBBWZPSBWRELCSBWSWYAJVJEEBVDJBBEEWCCSWSBBB : 5LAP : B :   96 :  143 : 0.635
   5. .SWSBWSBBWZPSBWRELCSBWSWYAJVJEEBVDJBBEEWCCSWSBBB : 5O1A : B :   96 :  143 : 0.635
   6. .SWSBWSBBWZPSBWRELCSBWSWYAJVJEEBVDJBBEEWCCSWSBBB : 5O1C : B :   96 :  143 : 0.635
   7. .SWSBWSBBWZPSBWRELCSBWSWYAJVJEEBVDJBBEEWCCSWSBBB : 5O1D : B :   96 :  143 : 0.635
   8. .SWSBWSBBWZPSBWRELCSBWSWYAJVJEEBVDJBBEEWCCSWSBBB : 5O1F : B :   96 :  143 : 0.635
   9. .SWSBWSBBWZPSBWRELCSBWSWYAJVJEEBVDJBBEEWCCSWSBBB : 5O1G : B :   96 :  143 : 0.635
  10. .SWSBWSBBWZPSBWRELCSBWSWYAJVJEEBVDJBBEEWCCSWSBBB : 5O1H : B :   96 :  143 : 0.635	   1. BEEBVPSWCYJBBEEEEEWSVJVAAAAPCSWCCYAAAAPSBBWYAPCYAJ : 5AB9 : A :  144 :  193 : 0.620
   2. BEEBVPSWCYJBBEEEEEWSVJVAAAAPCSWCCYAAAAPSBBWYAPCYAJ : 5AOI : A :  144 :  193 : 0.620
   3. BEEBVPSWCYJBBEEEEEWSVJVAAAAPCSWCCYAAAAPSBBWYAPCYAJ : 5AOM : A :  144 :  193 : 0.620
   4. BEEBVPSWCYJBEEEEEEWSVPYAAAAPCSWCCYAAAAPSBBWYAPCYAJ : 4KVP : D :  144 :  193 : 0.620
   5. BEEBVPSWCYJBBEEEEEWSVJVAAAAPCSWCCYAAAAPSBBWYAPCYAJ : 4LO9 : D :  144 :  193 : 0.620
   6. BEEBVPSWCYJBBEEEEEWSVJVAAAAPCSWCCYAAAAPSBBWYAPCYAJ : 4XR8 : C :  144 :  193 : 0.620
   7. BEEBVPSWCYJBBEEEEEWSVPYAAAAPCSWCCYAAAAPSBBWZAPCYAJ : 5ABA : B :  144 :  193 : 0.610
   8. BEEBVPSWCYJBBEEEEEWSVJVAAAAPCSWCCYAAAAPSBBWZAPCYAJ : 5AOI : B :  144 :  193 : 0.610
   9. BEEBVPSWCYJBBEEEEEWSVJVAAAAPCSWCCZAAAAPSBBWYAPCYAJ : 5AOK : A :  144 :  193 : 0.610
  10. BEEBVPSWCYJBBEEEEEWSVPYAAAAPCSWCCYAAAAPSVJWZAPCYAJ : 5AOK : B :  144 :  193 : 0.610	   1. VJBEVAJVAJEEBWSVDJBBEEEEWCSWCCCZAJVPREEEEEBVPCYJB : 1YCS : A :  194 :  242 : 0.612
   2. VJBEVAJVJBEEBWSVDJBBEBEEWCSWCCCZAJVPREBEEEBVPCYJB : 8DC4 : B :  194 :  242 : 0.602
   3. VJBEVAJVJBEEBWSVDJBBEEEEWCSWCCCZAJVPREBEBEBVPCYJB : 6FF9 : B :  194 :  242 : 0.602
   4. VJBEVAJVAJBEBBBVDJBBEEEEWCSWCCCZAJVJEEEEEEBVPCYJB : 3TS8 : A :  194 :  242 : 0.602
   5. VJBEVAJVAJBEBBBVDJBBEEEEWCSWCCCZAJVJEEEEEEBVPCYJB : 3TS8 : D :  194 :  242 : 0.602
   6. VJBEVAJVAJEEBBBVDJBBEBEEWCSWCCCZAJVPREBEEEBVPCYJB : 2AHI : D :  194 :  242 : 0.592
   7. VJBEVAJVJBBEBWSVDJBBEEEEWCSWCCCZAJVPREEEEEBVPCYAJ : 2BIN : A :  194 :  242 : 0.592
   8. VJEEVAJVJBBEBWSVDJBBEEEEWCSWCCCZAJVPRBBEBEBVPCYJB : 3D08 : A :  194 :  242 : 0.592
   9. VJBEVAJVAJBEBWSVDJBBEEEEWCSWCCCZAJVPREEEEEBVPCYJW : 2J1W : B :  194 :  242 : 0.592
  10. VJBEVAJVAJBEBWSVDJBBEEEEWCSWCCCZAJVPREEEEEBVPCYJW : 2J1Z : B :  194 :  242 : 0.592	   1. CYDJWSWREEEBEBEWCZQSWSWSEEELRBBEWYJVAAAADDAAAAAA : 2PCX : A :  243 :  290 : 0.667
   2. YDDJWSWREEEEEBEWCZQSBBVJEBELREEBWYJVAADDDDDAADAA : 2FEJ : A :  243 :  290 : 0.646
   3. YAQSWSWREEEBEBEWCZQSWSWSEEELRBEEWYJVADAAAAAAADA. : 3Q05 : C :  243 :  290 : 0.646
   4. YAQSWYJELREEEBEWCYQSWSWSEEELREEEWYJVAAAAAAAAAAAA : 2RMN : A :  274 :  321 : 0.646
   5. YADJWYJELREEEBEWCYQSWSWSEEELREEEWYJVAAAAAAAAAAAA : 2RMN : A :  274 :  321 : 0.646
   6. YADJWYJELREEEBEWCYQSWSWSEEELREEEWYJVAAAAAAAAAAAA : 2RMN : A :  274 :  321 : 0.646
   7. YADJWYJELREEEBEWCYQSWSWSEEELREEEWYJVAAAAAAAAAAAA : 2RMN : A :  274 :  321 : 0.646
   8. YAQSWYJELREEEBEWCYQSWSWSEEELREEEWYJVAAAAAAAAAAAA : 2RMN : A :  274 :  321 : 0.646
   9. YADJWYJELREEEBEWCYQSWSWSEEELREEEWYJVAAAAAAAAAAAA : 2RMN : A :  274 :  321 : 0.646
  10. YAQSWYJELREEEBEWCYQSWSWSEEELREEEWYJVAAAAAAAAAAAA : 2RMN : A :  274 :  321 : 0.646	


3-5Mix
	00000011111111111111111111111111111111111111111111 11111111111111111111111111111111111111111111111111 1111112222222222222222222222222222222222222222222 2222222222222222222222222222222222222222222222222222222
99999900000000001111111111222222222233333333334444 44444455555555556666666666777777777788888888889999 9999990000000000111111111122222222223333333333444 4444444555555555566666666667777777777888888888899999999
45678901234567890123456789012345678901234567890123 45678901234567890123456789012345678901234567890123 4567890123456789012345678901234567890123456789012 3456789012345678901234567890123456789012345678901234567
SSSVPSQKTYQGSYGFRLGFLHSGTAKSVTCTYSPALNKMFCQLAKTCPV QLWVDSTPPPGTRVRAMAIYKQSQHMTEVVRRCPHHERCSDSDGLAPPQH LIRVEGNLRVEYLDDRNTFRHSVVVPYEPPEVGSDCTTIHYNYMCNSSC MGGMNRRPILTIITLEDSSGNLLGRNSFEVRVCACPGRDQRTEEENLRKK	
3-5 Mix	..VLCABESVBYZCSAWEEACABASDYSJWJLEWVEJABEEACASASWBS BAEJVBSPCVJWBWEDEBWBVSVYADAVCDWDCSAAAPPBBJWZAWCZAD VEBBVPJWJAERBESVDPBDRWECWCSECSCEAYVCRRBAEEBVPSYVW QDVSSSBSAEABABZWWZPSSSASYEELEBVEAYPVWYAAJCYADAVA..	

   1. JLCSBWSBBWZPSBWREWCSBWSWYAJVJEEBVDJBBEEWCCSWSBBB : 8DC7 : A :   96 :  143 : 0.594
   2. JLCSBWSBBWZPSBWREWCSBWSWYAJVJEEBVDJBBEEWCCSWSBBB : 8DC8 : A :   96 :  143 : 0.594
   3. JLCSBWSBBWZPSBWRELCSBWSWYAJVJEEBVDJBBEEWCCSWSBBB : 3Q05 : C :   96 :  143 : 0.594
   4. .LCSBWSBBWZPSBWREWCSBWSWYAJVJEEBVDJBBEEWCYAPSBBE : 3D07 : B :   96 :  143 : 0.583
   5. ELCSBWSBBWZPSBWREWCSBWSWYAJWSEEBVDJBBEEWCCYPSBBE : 4IBY : A :   96 :  143 : 0.583
   6. JLCSBWSBBWZPSBWREWCSBWSWYJBVJEEBVDJBBEEWCCSWSBBE : 2H1L : W :   96 :  143 : 0.583
   7. JLCSBWSBBWZPSBWREWCSBWSWYAJVJEEBVDJBBEEWCCYPSBBE : 4QO1 : B :   96 :  143 : 0.583
   8. JLCSBWSBBWZPSBWREWCSBWSWYAJVJEEBVDJBBEEWCCYPSBBE : 2XWR : A :   96 :  143 : 0.583
   9. ..CSBWSBBWZPSBWRELCSBWSWYAJVJEEBVDJBBEEWCCSWSBBB : 5A7B : A :   96 :  143 : 0.573
  10. ..CSBWSBBWZPSBWRELCSBWSWYAJVJEEBVDJBBEEWCCSWSBBB : 5AB9 : A :   96 :  143 : 0.573	   1. BEEBVPSWCYJBBEEEEEWSVJVAAAAPCSWCCYAAAAJBBBWZAPCYAJ : 2AC0 : C :  144 :  193 : 0.660
   2. BEEBVPSWCYJBBEEEEEWSVJVAAAAPCSWCCYAAAAPSBBWZAPCYAJ : 5AOI : B :  144 :  193 : 0.660
   3. BEEBVPSWCYJBBEEEEEWSVJVAAAAPCSWCCYAAAAJBBBWZAPCYAJ : 8DC8 : A :  144 :  193 : 0.660
   4. BEEBVPSWCYJBBEEEEEWSVJVAAAAPCSWCCYAAAAPSBBWZAPCYAJ : 4HJE : A :  144 :  193 : 0.660
   5. BEEBVPSWCYJBBEEEEEWSVJVAAAAPCSWCCYAAAAPSBBWZAPCYAJ : 2J1Z : B :  144 :  193 : 0.660
   6. BEEBVPSWCYJBBEEEEEWSVJVAAAAPCSWCCYAAAAPSBBWZAPCYAJ : 2J20 : B :  144 :  193 : 0.660
   7. BEEBVPSWCYJBBEEEEEWSVJVAAAAPCSWCCYAAAAPSBBWYAPCYAJ : 5AB9 : A :  144 :  193 : 0.650
   8. BEEBVPCCCYJBBEEEEEWSVJVDAAAPCSWCCYAAAAPSBBWZAPCYAJ : 2AC0 : B :  144 :  193 : 0.650
   9. BEEBVPSWCYJBBEEEEEWSVJVAAAAPCSWCCYDAAAPSBBWZAPCYAJ : 2ADY : A :  144 :  193 : 0.650
  10. BEEBVPSWCYJBBEEEEEWSVJVAAAAPCSWCCYAAAAPSBBWYAPCYAJ : 5AOI : A :  144 :  193 : 0.650	   1. VJBEVAJVAJEEBWSVDJBWREEEWCSWCCCZAJVPREBEBEBVPCYJW : 2AC0 : A :  194 :  242 : 0.684
   2. VJBEVAJVJBEEBWSVDJBWREEEWCSWCCCZAJVPREBEEBBVPCYJW : 2AC0 : B :  194 :  242 : 0.684
   3. VJBEVAJVJBBEBWSVDJBWREEEWCSWCCCZAJVPREBEEEBVPCYJW : 4IBV : A :  194 :  242 : 0.684
   4. VJBEVAJVJBEEBWSVDJBWREEEWCSWCCCZAJVPREBEEEBVPCYAJ : 4LO9 : A :  194 :  242 : 0.684
   5. VJBEVAJVJBEEBBBVDJBWREEEWCSWCCCZAJVPREBEBEBVPCYJW : 4LO9 : B :  194 :  242 : 0.684
   6. VJBEVAJVJBEEBWSVDJBWREEEWCSWCCCZAJVPREBEEEBVPCYAJ : 4LO9 : C :  194 :  242 : 0.684
   7. VJBEVAJVJBBEBWSVDJBWREEEWCSWCCCZAJVPREBEEEBVPCYJW : 5MCT : A :  194 :  242 : 0.684
   8. VJBEVAJVJBBEBWSVDJBWREEEWCSWCCCZAJVPREBEEEBVPCYJW : 5MCT : B :  194 :  242 : 0.684
   9. VJBEVAJVJBBEBWSVDJBWREEEWCSWCCCZAJVPREBEEEBVPCYJW : 5MCW : A :  194 :  242 : 0.684
  10. VJBEVAJVJBBEBWSVDJBWREEEWCSWCCCZAJVPREBEEEBVPCYJW : 5MCW : B :  194 :  242 : 0.684	   1. YDQSWSWSEEEBEBEWCZQSWSWSEEELRBBEWYJVADAAAAAAAAAA : 4AGM : A :  243 :  290 : 0.583
   2. YDQSWSWSEEEBEBEWCZQSWSWSEEELRBBEWYJVADAAAAAAAAAA : 4AGN : A :  243 :  290 : 0.583
   3. YDQSWSWSEEEBEBEWCZQSWSWSEEELRBBEWYJVADAAADAAAAAA : 6GGC : A :  243 :  290 : 0.583
   4. YDQSWSWSEEEBEBEWCZQSWSWSEEELRBBEWYJVADAAADAAAAAA : 6GGE : A :  243 :  290 : 0.583
   5. YDQSWSWSEEEBEBEWCZQSWSWSEEELRBBEWYJVADAAAAAAAAAA : 5O1B : A :  243 :  290 : 0.583
   6. YDQSWSWSEEEBEBEWCZQSWSWSEEELRBBEWYJVADAAAAAAAAAA : 5O1C : A :  243 :  290 : 0.583
   7. YDQSWSWSEEEBEBEWCZQSWSWSEEELRBBEWYJVADAAAAAAAAAA : 5O1D : A :  243 :  290 : 0.583
   8. YDQSWSWSEEEBEBEWCZQSWSWSEEELRBBEWYJVADAAAAAAAAAA : 5O1E : A :  243 :  290 : 0.583
   9. YDQSWSWSEEEBEBEWCZQSWSWSEEELRBBEWYJVADAAAAAAAAAA : 6SI3 : A :  243 :  290 : 0.583
  10. VDQSWSWREEBBEBEWCZQSWSWSEEELRBBEWYJVADAAAAAAAAAA : 4XR8 : C :  243 :  290 : 0.573	


PFVM (Protein Folding Variation Matrix)
Conformation alignment in PFSC (Protein Folding Shape Code) for 10 given 3D structures, one Alphafold structure and 12 mutiple conformations from PFVM
A	Hundredth
    Tenth
  Digital
 Sequence	000000111111111111111111111111111111111111111111111111111111111111111111111111111111111111111111111111111122222222222222222222222222222222222222222222222222222222222222222222222222222222222222222222222222
999999000000000011111111112222222222333333333344444444445555555555666666666677777777778888888888999999999900000000001111111111222222222233333333334444444444555555555566666666667777777777888888888899999999
456789012345678901234567890123456789012345678901234567890123456789012345678901234567890123456789012345678901234567890123456789012345678901234567890123456789012345678901234567890123456789012345678901234567
SSSVPSQKTYQGSYGFRLGFLHSGTAKSVTCTYSPALNKMFCQLAKTCPVQLWVDSTPPPGTRVRAMAIYKQSQHMTEVVRRCPHHERCSDSDGLAPPQHLIRVEGNLRVEYLDDRNTFRHSVVVPYEPPEVGSDCTTIHYNYMCNSSCMGGMNRRPILTIITLEDSSGNLLGRNSFEVRVCACPGRDQRTEEENLRKK	
B	        1
        2
        3
        4
        5
        6
        7
        8
        9
       10
       11
       12
       13
       14
       15
       16
       17	..VSCSBWSBBWZPSBWRELCSBWSWYAJVJEEBVDJBBEEWCCSWSBBBBEEBVPSWCYJBBEEEEEWSVJVAAAAPCSWCCYAAAAPSBBWAAPCYAJVJBEVAJVJBEEBWSVDJBBREEEWCSWCCCZAJVPREBEEEBVPCYJWYDQSWSWSEEEBEBEWCZQSWSWSEEELRBEEWYJVAYSADCAAAAAA..
..CWWJVPBEEADJBYAABWSBWCAAAJWYSBBSAAPSAB LAYAPDPCEEBBDAVCCAPAJAABABAEAYPYDWVSJAAAASZDDDDJYJZAYYJWVVAAAWRUYBAEJBAABBAACSWEBLRASBBWYWYCDAJSBEBBBADJWAAJVADJAYJRBBBEBWYCAYAYPPVJASCWAEBBDASYDPJVAADDPVDD..
..JLVAPECVVYBCPALEAAAAPAJDVSBWPLWWCEAAJ  AVAYAAWVSWAYJCBLPYVYWDWADABABZSCYJDDVWDDDES   PABVJQZPWBZ DDEABWPWWAAAREEV QPADBWWCC EEVSAEWYPCERDA  D  SDVBQSVASVBEAJAAAEZVWAPBSBAPYB EESVUASP WAAJJWYJDDVV..
..PJSDJBESUPRWEESDSBRZLSPPPYEC R CEJEDD   WDJSRSSYA  WQARSJCSPSBVCLRBCEWPQSJJSSBLFWV   JSJYVJCSCSC  EBEWAJPRSELDD$  E J SARBJ  LPJV$VBB L  U     BZ AAJYQDPPBVCDJDSVBYWJCBVPVVW FB YAPB  YFCCVDZPJJPY..
..BCFCSJAWYJAVAUBQVEDEVPBYZCAE      BVP    ZDVPEEWS   EEPVSWPCERWWWDRDJYZPDPQ JW SB     WCASVDDB S  ZSLC$CAJVSDJJP  B   UJBSL    AJBDZY W  V      V PCPWZ DCCWW DLRBJBQYWCAEWSR PW SVBJ  C R  VPWWPJP..
..SPLPAARCC CQJ JBWSFPJYRBJDRA      Q      JPB  Y     WYWR ZWACDD   LVW  VY   ER J      QVWYCPWA    URCSCW EW  WWC  W   VPJDB    VSJPA            S DDYZY J  DP  WCWAJCDAADBCB  J   R C  P    YJYVWSJ..
..WBBVEDW A SAL CJYCJJAVCSEPLB      D       CJ         CJB A RWS    D    Z    DE R      YWPAPVQF    BDDAPS CB  BPL        YVE    WYPJV               JCA     LR    SEP$SVJCJBC        E        VBLE Q..
..AFYYY   W VRW FS FEREBDZ$BDD      S       E          JEF J SRJ              BJ         ACWBSJL    R VLE  BD  VLQ         WD     ZQY$               PQC      S    ASSP JVQQAJ        W        WCQC S..
..YE B    Z     I  VWWQRYJBESR      R       W          SYJ S EL                          PEC WVY    S  DY  SC  S            P      WQP                VS           CL$V PYYYYL        P        CSYY  ..
..ER W             JBVCJVVDW                             L B V                            SD Q Z       F                           DSQ                             RYV  ED DEP        R        S B   ..
..RY Z             RLYS WER                              E Q L                               E                                     RU                              J    ZO  LQ        L              ..
..DZ                Y I E                                                                    J                                     V                               P    DZ  UR        F              ..
..F                                                                                          $                                                                     D    LF            V              ..
..                                                                                                                                                                      $R                           ..
..                                                                                                                                                                      R                            ..
..                                                                                                                                                                      U                            ..
..                                                                                                                                                                      I                            ..
	


C	   2PCX-A	..JEWSBWSBEWZPSBWREBWSBBBWYAJVJEEBVDJBBEEWCCSWSBBBEEEBVPSWCYJBBEEEEEWSVJVAAAAPCSWCCYDAAAPSBBWZAPCYAJVJBEVAJVAJEEBBBVDJBBEBEEWCSBWCCZAJVPREEEEEBVJWYAPCYDJWSWREEEBEBEWCZQSWSWSEEELRBBEWYJVAAAADDAAAAAA..	
	   1GZH-A	 ..PCSBWSBBWZPSBWRELCSWCSWYAJVJEEBVDPSBEEWCCSWSBBBBEEBVPCCCYJBBEEEEEWSVJVDAAAPCSWCCYAAA..........YAJVJBE.....BBEBBBVDJBBEBEEWCSW........REEEEEBVPCYJWYDQSWSWSEEBBEBEWCZQSWSWSEEELRBEEWYJVADAAAAADAAAA..	
	   1TSR-A	..SWCSVPSBBWZPSBWRELSBBWSWYAJVJEEBVDJBBEEWCCYPSBBEEEEBVPCCCYJBBEEEEEWSVJVAAAAPCSWCCZAAAAPYJBWYAPCYAJVJWRWYJVJBEEBWSVDJBBEEEEWCSWCCCZAJVPREBEBBBVPCYAJVAQSWSWSEEBBEBEWCZQSWSWSEEELRBEBWYJVADAAAADAA..	
	   1YCS-A	   ..SBWSBEWZPSBWREWCSBWSWYAJVJEEBVDJBBEEWCCYPSBBBBEEBVPCCCYJBBEEEEEWSVJVAAAAPCSWCCZDAAAPYJBWYAPCYAJVJBEVAJVAJEEBWSVDJBBEEEEWCSWCCCZAJVPREEEEEBVPCYJBVDQSWSWREEEEEBEWCZQSWSWSEEELRBBBWYJVAAAADDA..	
	   2FEJ-1	..APCSBWSBVADJBBWRBWSEWCSWZJWYJEBBVDJBBEEWCCSWSBBBBEEBVPSWCYJBBEBEBEWSVJVDAAAJWSWCCYAADAAJVJWCYJWYAJVJEEVJBVJBEEBBBVDJBBVJEELCSWCCCYDJVPSBBEEEBVJWYAJVDDJWYJEEEEBEBEWCZQSBBVJEEELREEBWYJVDADDDDDDDDAA..	
	   2MEJ-1	  ..WSBWSBBWZPSBWREWSBBWSBVAJWSEEBVDPSBEELCCSWSBBEBBBBVPSWCYJWSEBEEEWSVJVAAAAPCSWCCYAAAAJBVJWZAJWYAJVJWRVAJVJBEEBBBVQCSBBBEEWCSWCSWZAJVPSBBBBBBVPCYAPYDDJWSWRBEBBEBEWCZQSWSWSEEELRBEUPYJVADAAADDDAA..	
	   2YBG-A	  ..CSBWSBBWZPSBWREWCSBWSWYAJWSEEBVDJBBEEWCCYPSBBEEEEBVPSWCYJBBEEEEEWSVJVAAAAPCSWCCZDADAPYJBWYAPCYAJVJBEVAJVJBEEBWSVDJBWREEEWCSWCC.....PRBBEBBBVPCYJWYAQSWSWSEEEBEBEWCZQSWSWSEEELRBBEWYJVADADAAAA..	
	   5BUA-A	..VPCSBWSBBWZPSBWRELCSBWSWCSWYJEEBVDJBBEEWCCSWSBBBBEEBVPCCCYJBBEEEEEWSVJVDAAAPCSWCCYDAAAJBBBWYAPCYAJVJBEVAJVJBBEBWSVDJBWREEEWCSWCCCZAJVPREBEEBBVPCYJWYDQSWSWREEBBEBEBWZQSWSWSEEELRBBEWYJVADAAAAAAAAAD..	
	   5LGY-A	 ..SWSBBBBBWZPSBBEELCSBWSWYAJVJEEBVDJBBEEWCCSWSBBEEEEBVPSWCYJBBEEEEEWSVJVDAAAPCSWCCYAAAAPYAJWZAPCYAJVJWRWYJVJBEEBBBVDJBWREEEWCSWCCCZAJVPREBEEEBVPCYJWYDQSWSWREEEBEBEBWZQSWSWSEEELRBBEWYJVADAAADAAAAAA..	
	   2AC0-A	..CSWSBWSBBWZPSBWREWSBWCSWYJBVJEEBVDJBBEEWCCYPSBBBBEEBVPSWCYJBBEEEEEWSVJVDAAAPCSWCCYAAAAPYJBWYAPCYAJVJBEVAJVAJEEBWSVDJBWREEEWCSWCCCZAJVPREBEBEBVPCYJWYDQSWSWREEBBEBEWCZQSWSWSEEELRBBEWYJVADAAAAAAAAAA..	
	   6XRE-M	..AJVJVPRWZJVAJBBBWCYJEVPYJWSVPREBVDPSBEEWCYAPRBBBBEEBVPCCCYJBEEEEEEWSVPCYAAAJWSWFSVAAAAJVJBWYAJWYAJVJEEVAJVAJEEBWSVDJBBEBEEWSBWCCCZJVAJLREEEEBVJWYAPYAQSWYJELREBEBEWCYQSWSWSLREWRBEBBVPYAAAAAADDAADQ..	
	   8F2I-A	..YPCSVPRBBWZPSEWRELSVJWYPYAJWYPFSVDJVPREBWYAPSEBBBBEWYPSWCYAJEEEEEELFYPYAAAAPCYPCCYAAAAJVAPSVAPCYAJVJEBBWCZAJEEELSVDPYJEEEEWFRLCCCYAJWCREEEEEBVJWYAPYAPSVJBEWREEEEEWCZQSWSWSEEELREEEWYJVAAAAAAAAAADP..	
D	AlphaFold	..JLCSBWSBBWZPSBWREWCSWCSWYAJVJEEBVDPSBEEWCCSWSBBBBEEBVPCCCYJBBEEEEEWSVJVAAAAPCSWCCYAAAAJVJBWYAPCYAJVJEEVAJVJBBEBBBVDJBBEBEEWCSWCCCZAJVPREEEEEBVPCYAPYAQSWSWSEEBBEBEWCZQSWSWSEEELRBBEWYJVADAAADDAAAAA..	


E	  PFVM-01	..VSCSBWSBBWZPSBWRELCSBWSWYAJVJEEBVDJBBEEWCCSWSBBBBEEBVPSWCYJBBEEEEEWSVJVAAAAPCSWCCYAAAAPSBBWAAPCYAJVJBEVAJVJBEEBWSVDJBBREEEWCSWCCCZAJVPREBEEEBVPCYJWYDQSWSWSEEEBEBEWCZQSWSWSEEELRBEEWYJVAYSADCAAAAAA..	
	 Coupling	..VJWJBWABBWDPSBWAAAAAPCSWAAJVJEEBVDJBBEEWAAAPSBBBBEEBVPSWAPYJBEEEEEWAJWPYAAAPCSWACYAAAAPSBBWAAPCYAJVJBEVAJVJBEEBWSVDJBBEEEEWCSWPCCZAJVPREBEEEBVPCYJWQSVJWPCSEEEBEBEWAAQSWPYJEEELRBEEWAJVAPSVDAAAAAAA..	
	  Mix12-1	..VSWSBWBBBAZPBBWRBLCBBWAWYAWVJBEBADJBAEELCCAWSBCBBBEBAPSWAYJJBEBEEEESVPVAWAAPASWACYDAAAJSBZWAYPCYVJVABEUAJVEBEABWBVDJSBRBEEACSWWCCYAJAPREEEEBBVJCYJJYDDSWYWSEBEBBBECCZQYWSVSESELREEEDYJYAYSVDCDAAVAA..	
	  Mix12-2	..CSCSVWSBEWDPSBARELSSWWSWAAJVSEBBVDPBBEEWACSWDBBBEEBBVPCWCYABAEEEBEWSYJYAAASPCSACSYAADAPSJBAAAPWYAJAJWEVABVJBBEAWSVAJBBEELEWCBWCCWZCJVPSEBEBEAVPCAJWYAQJWSWREEEEEWEWCYQSWPWJEEEWRBEBWAJVAPSADAADAAAD..	
	  Mix12-3	..VWCSBPSBBAZJSBWAELCBBCSWYJJVJBESVDJSBEELCYSWSPBBBBEDVPSCCYJJBAEEEAWSVPVDAAAJCSWACZAAADPSBZWYAPCVAJVABRVAJAJBEABBSVDCBBRBERWCSBCCCYADVPRBBEEBBDPCYAWYDDSASWSBEEBBBYWCZASWSVSAEELABEEDYSVAYJADCDAPAAA..	
	  Mix12-4	..CSWSVWBBEWDPBBARBLSSWWAWAAWVSEBBADPBAEEWACAWDBCBEEBBAPCWAYABAEBEBEESYJYAWASPASACSYDADAJSJBAAYPWYVJAJWEUABVEBBEAWBVAJSBEELEACBWWCWZCJAPSEEEBEAVJCAJJYAQJWYWREBEEEWECCYQYWPWJESEWREEBWAJYAPSVDAADAVAD..	
	  Mix12-5	..VWCJBPSEBAZJSYWAEWCBBCSAYJJYJBESVAJSBBELCYSPSPBEBBEDVVSCCPJJBAEAEAWAVPVDAVAJCAWACZADADPYBZWYAJCVAAVABRVYJAJJEABBSADCBWRBERWSSBCYCYADVJRBBBEBBDPWYAWVDDSASJSBEBBBBYWAZASPSVSAECLABBEDYSVDYJAACDAPADA..	
	  Mix13-1	..VSVSBWCBBYZPPBWRALCABWJWYABVJLEBCDJBJEEACCYWSBVBBAEBCPSWYYJWBEAEEEASVSVAJAAPWSWDCYAAAAASBJWAPPCYAJVEBEWAJVABERBWVVDJABRWEECCSWVCCEAJPPREDEEEBVPCYJBYDVSWVWSEJEBABEVCZQBWSASEBELRSEEAYJVAYSJDCYAADAA..	
	  Mix13-2	..JSCSPWSBVWBPSBLRELASPWSWVAJVPEWBVDABBEEWVCSWABBBWEYBVPLWCYYBDEEEAEWSZJCAAADPCSDCEYAAAAPSVBQAAPBYAJDJAEVAWVJBAEEWSVQJBBBEWEWCEWCCAZWJVPEEBEEEDVPCDJWYSQAWSWEEEEAEEEWCAQSWBWPEEEERBEUWSJVAASADWAJAAAV..	
	  Mix13-3	..VLCSBESBBYZCSBWEELCABASWYSJVJLEWVDJABEEACASWSWBBBAEJVPSPCYJWBWEEEBWSVSVYAAAVCSWDCSAAAPPSBJWZAPCZAJVEBBVAJWJBERBESVDPBBRWECWCSECCCEAYVPRRBEEEBVPCYVWYDVSSSWSAEEBABZWCZPSWSASYEELEBEEAYPVAYAADCYADAAA..	
	  Mix13-4	..JSVSPWCBVWBPPBLRALASPWJWVABVPEWBCDABJEEWVCYWABVBWEYBCPLWYYYBDEAEAEASZJCAJADPWSDCEYAAAAASVBQAPPBYAJDJAEWAWVABAEEWVVQJABBEWECCEWVCAZWJPPEEDEEEDVPCDJBYSQAWVWEEJEAEEEVCAQBWBWPEBEERSEUWSJVAASJDWAJADAV..	
	  Mix13-5	..VLCABESVBYZCSAWEEACABASDYSJWJLEWVEJABEEACASASWBSBAEJVBSPCVJWBWEDEBWBVSVYADAVCDWDCSAAAPPBBJWZAWCZADVEBBVPJWJAERBESVDPBDRWECWCSECSCEAYVCRRBAEEBVPSYVWQDVSSSBSAEABABZWWZPSSSASYEELEBVEAYPVWYAAJCYADAVA..	
