# Supplementary material for: Conformational ensembles for protein structure prediction
Source: Sci Rep. 2025 Mar 12;15:8513. doi: 10.1038/s41598-024-84066-z (PMC11904239; doi:10.1038/s41598-024-84066-z)
Supplement: Supplementary file 3 — Supplementary Information 3. [file 41598_2024_84066_MOESM3_ESM.rtf]

LEF1_HUMAN
Q9UJU2 · LEF1_HUMAN

Intrinsically disordered information


PFVM (Protein Folding Variation Matrix)
     000000000000000000000000000000000000000000000000000000000000000000000000000000000000000000000000000111111111111111111111111111111111111111111111111111111111111111111111111111111111111111111111111111122222222222222222222222222222222222222222222222222222222222222222222222222222222222222222222222222223333333333333333333333333333333333333333333333333333333333333333333333333333333333333333333333333333       
     000000000111111111122222222223333333333444444444455555555556666666666777777777788888888889999999999000000000011111111112222222222333333333344444444445555555555666666666677777777778888888888999999999900000000001111111111222222222233333333334444444444555555555566666666667777777777888888888899999999990000000000111111111122222222223333333333444444444455555555556666666666777777777788888888889999999999
     123456789012345678901234567890123456789012345678901234567890123456789012345678901234567890123456789012345678901234567890123456789012345678901234567890123456789012345678901234567890123456789012345678901234567890123456789012345678901234567890123456789012345678901234567890123456789012345678901234567890123456789012345678901234567890123456789012345678901234567890123456789012345678901234567890123456789
     MPQLSGGGGGGGGDPELCATDEMIPFKDEGDPQKEKIFAEISHPEEEGDLADIKSSLVNESEIIPASNGHEVARQAQTSQEPYHDKAREHPDDGKHPDGGLYNKGPSYSSYSGYIMMPNMNNDPYMSNGSLSPPIPRTSNKVPVVQPSHAVHPLTPLITYSDEHFSPGSHPSHIPSDVNSKQGMSRHPPAPDIPTFYPLSPGGVGQITPPLGWQGQPVYPITGGFRQPYPSSLSVDTSMSRFSHHMIPGPPGPHTTGIPHPAIVTPQVKQEHPHTDSDLMHVKPQHEQRKEQEPKRPHIKKPLNAFMLYMKEMRANVVAECTLKESAAINQILGRRWHALSREEQAKYYELARKERQLHMQLYPGWSARDNYGKKKKRKREKLQESASGTGPRMTAAYI    
   1 ..BPCWACCCCWBCVAAAAEZAAVBVCYAPYVABPAAAAJAEAVAPWASAAAAEAAAACCJAABEAYPYPCAADAAAJAAJUPJOAAADJEDPYBCVACLCJBALSEAAAWJPEEBLYWUPASUPJWAYAAVCBSCPABVRPSEBCYWSAQBVVWAAECWAVJABBYAJJWBLWSAJAAVYYABAJPUYCCAPALCWSAJYSPAAAWPYCAAAAJWCWAEEVPZAWQVYWAJEEVBAAAEEAWBBSLBPCCWRAREPVSBCAECBPBLAYDCYAWZZAAADJRVSWAAAEAVJWCCCSJVCVAAAAADAADDAAAAAADWEPYAAEAAAAAAAADAWAAACZAVAAAAADAAAAAADAAADDPVAJBBADAAAAASPWAVAAAEAADEBRPSSAAAA..
   2 .. CWVCPPPPAPPWYDDEWYDBBEBSBWVSYJPADDEWAD BBJAAJJWDDJJEVDDAYAVJWSCV$CWSSDADPECYFP JA  DDRPVAAAYEYJYPEEA  CADBJBBDB    B   B   VJSJJCWCCWLVWAEWEPWPV J SSJAAWBWECB  JACE SWBE PCYAVSJA  EWDC VWVWCCAJBVEWZACWBCECCSYVB  PSSB BSYEVAY V CSJWPWWDBA J     WCPSC E VSPCSSBWVEW  D  J    WYD B SS    SADAYAVJ  ACACDWZ    D BD QEEW ARA EDACDQDDDWSP AD BAYDYDD CJAJDPWJDA  EA CBJAWDSWJVPYBAEAWADDDADVYWYBD YBDDE..
   3 .. APPYWWWWCYSCDJ J VPW SSWCVWPCDWWVWDBDB  W CYVAYCJVAVCWWVZSEBEWSJ SJJDJWSD A J   W  VJS WSVVAJCDSYYAP  JBJEDJEA         J   Y CWBDPV YWD YBBBA B  A  JYDCCEDWPR  DWWW PSLA  VVPBD Z  R AJ  YYCSSESAAVCVPABWRCAWB  V  CBA   C$AB B C YAABWVB EJ P     EWSYA   UWWRCWWBEWB  J       CBV A  W    DVYP DSA   WPWSDE    S AJ VBBD   E DJJWVWYWPDPA    VVA AJS  EEBPJDDWB  DR YWVBJVYAZSYBJPBSJJYVBVJDAAPSC AWC W..
   4 .. WSYJAAAAPCJAJV V BVR  CZA AC  AYBJVEW     DDSYPSSYCDPJEYABJ PCWS JAV  B J P L      J A  ZJZVPEPAFR    R PVYESW               JPPS S SEJ SSAJW E     AS PDDLDYS  PPLZ B     YWYSW      V   JSBJBS EBP RQBDCDLWAW     AEB   UBBJ E   JRPABAC    Y       JBP   BC WWRE J C          YV     C    E JS V B   AWSV Y       S DYRC     CYCDJDSVJVCE    PWC B    RLDVSVSB      J SWCYVVSW JPCWJSBE JDSJJJVAS R   R..
   5 .. SAEWJJJJJABYVB L CSC  PES BB  SJZS PS     QJPZJVBDPB LJJDDC ADVC   W  J   S W      P B   WBCBBQVJA    Z YWBSP                BCEW   ZBB D CAB       CC JS ALZW   D S Y     W CEB          S SWW   CS SJJV WP JY     B     EIWD     SBBLYJV            VV    LA BEV  W S          VZ            BW P W   B RR         P SWYB     VZVVRC P  WS    JJL D    DREJQLBJ      L  ELSDCYP CERABDWJ  CBS VWVZ V    ..
   6 .. RRSVVVVVSZEDS     WJ    P YJ   ZPE        JCYDSJESWW PQBLCD DYY    P  V   R Y      S E    SD   PW     P RCCAC                VBV     CY J LVS       PF    UBE    V B A        DJ            J     FB BYSY FB SA           PJL      VVWCEPD            WW    WY FRB  B Y          P             SJ Q     D B            WDWJ     PCWBWS    DW    DPV S    LBWYCYP       B  RSCJP D VWJDCYDP  WV  YCJA      ..
   7 .. LBZSSSSSEWW B     CD       Q    W         YPDPVPCRBJ CBWSEW  B     A  E   Y        Y      JP   BC     Q CFVUU                WRC     J  W EC        W     JS     L C V         P                  RY Q$WC JS  L           WAC      WDSSJUE            BA    SB LFY    A          Q             E        Q A            CV L     QSBF V     Y    FYE Q    W  SY V       Z  P RP  Q WVDSVCSS  B   SJYB      ..
   8 .. VJCZYYYYRJA W              W              VSBWC R  F VS WYB  A     Y  H   E        Z       Z    V     Y VPWLA                DSR     F  B SR        D     P      E P C         V                   W ABYZ     V           YCS       PVDZCL            DF    CV EP     V                        P        S J             P E     BVDJ J     B    L D W        B         A  V WC    ZCBVEEYW  L   BSXW      ..
   9 .. YVAPBBBBYVR Z                             SUCBB V  U S   PL  V        I   Z                J    E        DERY                ZYF     V     W                     J V W         Z                     EDQ      R           JEY       ECVCD             Q        J      E                        C          P               Q     WBPI $     V      W          E         E  I       DSVCPBCR      PAE       ..
  10 .. FFQREEEEB V                               WVE   W  P B   Q            P                    Q             J CV                 EL           Y                                                                               D        YYF S                      A      F                        U                                LEYS E                                 W  S       PZYQYVFC      UFP       ..
  11 .. B BBFFFFI Y                                 U   P  Y E   V                                 R               VQ                 V                                                                                                     CDJ Y                      P      J                        V                                ZP   L                                             D$JQLQV      ZL        ..
  12 .. E IEZZZZV                                   Q   Y        W                                 S               D$                                                                                                                                                                                  W                                 W                                                 YELRR        DR        ..
  13 .. D DQLLLL                                    W                                                              Y                                                                                                                                                                                                                                                                       LFYU                   ..
  14 ..   J$RRRR                                                                                                   I                                                                                                                                                                                                                                                                       $W I                   ..
  15 ..   OFQQQQ                                                                                                   P                                                                                                                                                                                                                                                                       Q  $                   ..
  16 ..    UDDDD                                                                                                   Z                                                                                                                                                                                                                                                                       R  D                   ..
  17 ..    DUUUU                                                                                                                                                                                                                                                                                                                                                                           F  F                   ..
  18 ..    IOOOO                                                                                                                                                                                                                                                                                                                                                                           U  L                   ..
  19 ..    LHHHH                                                                                                                                                                                                                                                                                                                                                                                                  ..
  20 ..    HIIII                                                                                                                                                                                                                                                                                                                                                                                                  ..
  21 ..    XXXXX                                                                                                                                                                                                                                                                                                                                                                                                  ..
  22 ..    O$$$$                                                                                                                                                                                                                                                                                                                                                                                                  ..
AF    ..IBUJELXOXIEEVAIVIBBBBWSBBBBBBVAAAAAAAAAAJVJVAAAADAADAAAAAAAAJWSWCFREUIELFRUIUORUOXORELREELREUIEUIELREELFRLFREEEBEBLFRUPXIUPRLREUPCCSWSBBBBBWSBEBEWSWCCYAJLYAJBBVJLREEEUHIELYOXHPRLCYPRELRUIEUIELREEELFXHIEBEEWSWSBVAJLRBWCSLSWYOREEEEBEEBEEUHIEEBBBEEEEELFREUHIEEBVPYJELRLFXIBBBVJEWYAPSBWSVAAAAAAPSWCCSBWCCSWZAADAAADAAAAAADAJVJVJVADAAAAAADAAAAPCZADAAAAADAAAAAADAAADDJVJBBVADDAAAAAAAAAAAAAAAAJBWCSWCSBB..
   1 ..BPCWACCCCWBCVAAAAEZAAVBVCYAPYVABPAAAAJAEAVAPWASAAAAEAAAACCJAABEAYPYPCAADAAAJAAJUPJOAAADJEDPYBCVACLCJBALSEAAAWJPEEBLYWUPASUPJWAYAAVCBSCPABVRPSEBCYWSAQBVVWAAECWAVJABBYAJJWBLWSAJAAVYYABAJPUYCCAPALCWSAJYSPAAAWPYCAAAAJWCWAEEVPZAWQVYWAJEEVBAAAEEAWBBSLBPCCWRAREPVSBCAECBPBLAYDCYAWZZAAADJRVSWAAAEAVJWCCCSJVCVAAAAADAADDAAAAAADWEPYAAEAAAAAAAADAWAAACZAVAAAAADAAAAAADAAADDPVAJBBADAAAAASPWAVAAAEAADEBRPSSAAAA..                                         


Conformation Alignment for structures of Alphafold prediction and mutiple conformations from PFVM
Alignment in PFSC (Protein Folding Shape Code)

A		000000000000000000000000000000000000000000000000000000000000000000000000000000000000000000000000000111111111111111111111111111111111111111111111111111111111111111111111111111111111111111111111111111122222222222222222222222222222222222222222222222222222222222222222222222222222222222222222222222222223333333333333333333333333333333333333333333333333333333333333333333333333333333333333333333333333333       
000000000111111111122222222223333333333444444444455555555556666666666777777777788888888889999999999000000000011111111112222222222333333333344444444445555555555666666666677777777778888888888999999999900000000001111111111222222222233333333334444444444555555555566666666667777777777888888888899999999990000000000111111111122222222223333333333444444444455555555556666666666777777777788888888889999999999
123456789012345678901234567890123456789012345678901234567890123456789012345678901234567890123456789012345678901234567890123456789012345678901234567890123456789012345678901234567890123456789012345678901234567890123456789012345678901234567890123456789012345678901234567890123456789012345678901234567890123456789012345678901234567890123456789012345678901234567890123456789012345678901234567890123456789
MPQLSGGGGGGGGDPELCATDEMIPFKDEGDPQKEKIFAEISHPEEEGDLADIKSSLVNESEIIPASNGHEVARQAQTSQEPYHDKAREHPDDGKHPDGGLYNKGPSYSSYSGYIMMPNMNNDPYMSNGSLSPPIPRTSNKVPVVQPSHAVHPLTPLITYSDEHFSPGSHPSHIPSDVNSKQGMSRHPPAPDIPTFYPLSPGGVGQITPPLGWQGQPVYPITGGFRQPYPSSLSVDTSMSRFSHHMIPGPPGPHTTGIPHPAIVTPQVKQEHPHTDSDLMHVKPQHEQRKEQEPKRPHIKKPLNAFMLYMKEMRANVVAECTLKESAAINQILGRRWHALSREEQAKYYELARKERQLHMQLYPGWSARDNYGKKKKRKREKLQESASGTGPRMTAAYI    	
B	     1
     2
     3
     4
     5
     6
     7
     8
     9
    10
    11
    12
    13
    14
    15
    16
    17
    18
    19
    20
    21
    22	..BPCWACCCCWBCVAAAAEZAAVBVCYAPYVABPAAAAJAEAVAPWASAAAAEAAAACCJAABEAYPYPCAADAAAJAAJUPJOAAADJEDPYBCVACLCJBALSEAAAWJPEEBLYWUPASUPJWAYAAVCBSCPABVRPSEBCYWSAQBVVWAAECWAVJABBYAJJWBLWSAJAAVYYABAJPUYCCAPALCWSAJYSPAAAWPYCAAAAJWCWAEEVPZAWQVYWAJEEVBAAAEEAWBBSLBPCCWRAREPVSBCAECBPBLAYDCYAWZZAAADJRVSWAAAEAVJWCCCSJVCVAAAAADAADDAAAAAADWEPYAAEAAAAAAAADAWAAACZAVAAAAADAAAAAADAAADDPVAJBBADAAAAASPWAVAAAEAADEBRPSSAAAA..
.. CWVCPPPPAPPWYDDEWYDBBEBSBWVSYJPADDEWAD BBJAAJJWDDJJEVDDAYAVJWSCV$CWSSDADPECYFP JA  DDRPVAAAYEYJYPEEA  CADBJBBDB    B   B   VJSJJCWCCWLVWAEWEPWPV J SSJAAWBWECB  JACE SWBE PCYAVSJA  EWDC VWVWCCAJBVEWZACWBCECCSYVB  PSSB BSYEVAY V CSJWPWWDBA J     WCPSC E VSPCSSBWVEW  D  J    WYD B SS    SADAYAVJ  ACACDWZ    D BD QEEW ARA EDACDQDDDWSP AD BAYDYDD CJAJDPWJDA  EA CBJAWDSWJVPYBAEAWADDDADVYWYBD YBDDE..
.. APPYWWWWCYSCDJ J VPW SSWCVWPCDWWVWDBDB  W CYVAYCJVAVCWWVZSEBEWSJ SJJDJWSD A J   W  VJS WSVVAJCDSYYAP  JBJEDJEA         J   Y CWBDPV YWD YBBBA B  A  JYDCCEDWPR  DWWW PSLA  VVPBD Z  R AJ  YYCSSESAAVCVPABWRCAWB  V  CBA   C$AB B C YAABWVB EJ P     EWSYA   UWWRCWWBEWB  J       CBV A  W    DVYP DSA   WPWSDE    S AJ VBBD   E DJJWVWYWPDPA    VVA AJS  EEBPJDDWB  DR YWVBJVYAZSYBJPBSJJYVBVJDAAPSC AWC W..
.. WSYJAAAAPCJAJV V BVR  CZA AC  AYBJVEW     DDSYPSSYCDPJEYABJ PCWS JAV  B J P L      J A  ZJZVPEPAFR    R PVYESW               JPPS S SEJ SSAJW E     AS PDDLDYS  PPLZ B     YWYSW      V   JSBJBS EBP RQBDCDLWAW     AEB   UBBJ E   JRPABAC    Y       JBP   BC WWRE J C          YV     C    E JS V B   AWSV Y       S DYRC     CYCDJDSVJVCE    PWC B    RLDVSVSB      J SWCYVVSW JPCWJSBE JDSJJJVAS R   R..
.. SAEWJJJJJABYVB L CSC  PES BB  SJZS PS     QJPZJVBDPB LJJDDC ADVC   W  J   S W      P B   WBCBBQVJA    Z YWBSP                BCEW   ZBB D CAB       CC JS ALZW   D S Y     W CEB          S SWW   CS SJJV WP JY     B     EIWD     SBBLYJV            VV    LA BEV  W S          VZ            BW P W   B RR         P SWYB     VZVVRC P  WS    JJL D    DREJQLBJ      L  ELSDCYP CERABDWJ  CBS VWVZ V    ..
.. RRSVVVVVSZEDS     WJ    P YJ   ZPE        JCYDSJESWW PQBLCD DYY    P  V   R Y      S E    SD   PW     P RCCAC                VBV     CY J LVS       PF    UBE    V B A        DJ            J     FB BYSY FB SA           PJL      VVWCEPD            WW    WY FRB  B Y          P             SJ Q     D B            WDWJ     PCWBWS    DW    DPV S    LBWYCYP       B  RSCJP D VWJDCYDP  WV  YCJA      ..
.. LBZSSSSSEWW B     CD       Q    W         YPDPVPCRBJ CBWSEW  B     A  E   Y        Y      JP   BC     Q CFVUU                WRC     J  W EC        W     JS     L C V         P                  RY Q$WC JS  L           WAC      WDSSJUE            BA    SB LFY    A          Q             E        Q A            CV L     QSBF V     Y    FYE Q    W  SY V       Z  P RP  Q WVDSVCSS  B   SJYB      ..
.. VJCZYYYYRJA W              W              VSBWC R  F VS WYB  A     Y  H   E        Z       Z    V     Y VPWLA                DSR     F  B SR        D     P      E P C         V                   W ABYZ     V           YCS       PVDZCL            DF    CV EP     V                        P        S J             P E     BVDJ J     B    L D W        B         A  V WC    ZCBVEEYW  L   BSXW      ..
.. YVAPBBBBYVR Z                             SUCBB V  U S   PL  V        I   Z                J    E        DERY                ZYF     V     W                     J V W         Z                     EDQ      R           JEY       ECVCD             Q        J      E                        C          P               Q     WBPI $     V      W          E         E  I       DSVCPBCR      PAE       ..
.. FFQREEEEB V                               WVE   W  P B   Q            P                    Q             J CV                 EL           Y                                                                               D        YYF S                      A      F                        U                                LEYS E                                 W  S       PZYQYVFC      UFP       ..
.. B BBFFFFI Y                                 U   P  Y E   V                                 R               VQ                 V                                                                                                     CDJ Y                      P      J                        V                                ZP   L                                             D$JQLQV      ZL        ..
.. E IEZZZZV                                   Q   Y        W                                 S               D$                                                                                                                                                                                  W                                 W                                                 YELRR        DR        ..
.. D DQLLLL                                    W                                                              Y                                                                                                                                                                                                                                                                       LFYU                   ..
..   J$RRRR                                                                                                   I                                                                                                                                                                                                                                                                       $W I                   ..
..   OFQQQQ                                                                                                   P                                                                                                                                                                                                                                                                       Q  $                   ..
..    UDDDD                                                                                                   Z                                                                                                                                                                                                                                                                       R  D                   ..
..    DUUUU                                                                                                                                                                                                                                                                                                                                                                           F  F                   ..
..    IOOOO                                                                                                                                                                                                                                                                                                                                                                           U  L                   ..
..    LHHHH                                                                                                                                                                                                                                                                                                                                                                                                  ..
..    HIIII                                                                                                                                                                                                                                                                                                                                                                                                  ..
..    XXXXX                                                                                                                                                                                                                                                                                                                                                                                                  ..
..    O$$$$                                                                                                                                                                                                                                                                                                                                                                                                  ..	
C	AF	..IBUJELXOXIEEVAIVIBBBBWSBBBBBBVAAAAAAAAAAJVJVAAAADAADAAAAAAAAJWSWCFREUIELFRUIUORUOXORELREELREUIEUIELREELFRLFREEEBEBLFRUPXIUPRLREUPCCSWSBBBBBWSBEBEWSWCCYAJLYAJBBVJLREEEUHIELYOXHPRLCYPRELRUIEUIELREEELFXHIEBEEWSWSBVAJLRBWCSLSWYOREEEEBEEBEEUHIEEBBBEEEEELFREUHIEEBVPYJELRLFXIBBBVJEWYAPSBWSVAAAAAAPSWCCSBWCCSWZAADAAADAAAAAADAJVJVJVADAAAAAADAAAAPCZADAAAAADAAAAAADAAADDJVJBBVADDAAAAAAAAAAAAAAAAJBWCSWCSBB..	
D	PFVM-01	..BPCWACCCCWBCVAAAAEZAAVBVCYAPYVABPAAAAJAEAVAPWASAAAAEAAAACCJAABEAYPYPCAADAAAJAAJUPJOAAADJEDPYBCVACLCJBALSEAAAWJPEEBLYWUPASUPJWAYAAVCBSCPABVRPSEBCYWSAQBVVWAAECWAVJABBYAJJWBLWSAJAAVYYABAJPUYCCAPALCWSAJYSPAAAWPYCAAAAJWCWAEEVPZAWQVYWAJEEVBAAAEEAWBBSLBPCCWRAREPVSBCAECBPBLAYDCYAWZZAAADJRVSWAAAEAVJWCCCSJVCVAAAAADAADDAAAAAADWEPYAAEAAAAAAAADAWAAACZAVAAAAADAAAAAADAAADDPVAJBBADAAAAASPWAVAAAEAADEBRPSSAAAA..	
	Coup	..BPCWACCCCCYPCYAAAEVAAVBVCYAPYVAPYAAAAJBEBVAPYAJWAAAJEVAAAAJVAPSWJPYPCSADAAAJAAJUPJOAAADJEDPYAJVAAPCJBALSEVAAJBWEEBLYWUPAJUPJWAJWADPCSWPYBVRWJEBBVWJAQSVAAAADDPRVJAABEAJWWALWSVJVAJYYABWJPUYJVAPCSCWAAJVAPYAAPCYAAAAAJWCSBEEVPZAAQVYWAJEEVAAAAAEAWBBSLBWPCCREREWPSBWAEVBWBLAYDJYAWZPYAADJRVSWAAAAAAJWCCCSJVAAAAAAADAADDAAAAAADAEEYAAAAAAAAAAADAAAAAAAAAAAAAADAAAAAADAAADDPVAJBBVDAAAAAAPSWAAAAAAADAPRBSVAAAA..	
	Mix12-1	..BPCWACCPCWBPVADAAWZAABBVSYAVYVAPPADAAAAEABAPAASWAAAJAADACYJAAWEAVPYWCAAAAAEJAFJUPAOADADPEDPABCYACPCJBALSAAAJWJPBEBLYWUPASUPJVAYJAVCCSCLABARPSPBCVWSAQBVAWABECCAVJJBBEAJWWBLPSAAAAJYYAEAJCUYWCAPCLCBSAWYSPWAAEPYSAAAAJWSWAEEVPEAWYVYWAJEWVBWAAAEAWBBSLBPPCWRERESVSSCAEVBPBLAYDCYAWZWAAADJRSSWAAAAAVJACCCSJCCVAWAAADADDDAAAAEADAEPYEAECAADAAASDAAAABCZAYAAAAAAAAAWAAAAAEDDPBAJWBAWAAAYASEWAAAAAAAAYEBBPSSBAAE..	
	Mix12-2	..BPCWAPCCCABCVYADAEZDAVBBCBAPYYABPDAEAJAEAVAAWJSAADAEAVADCCJVABECY$YPCSADAPACAAJUPJOAADDJEAPYBEVJCLCEBALCEDAAWBPEEBLYWUPASUPJWJYAACCBSWPVBVRWSEBPYWSAQSVVWWAWCWAVJABCYAJJWELWSYJVAVYYABADPUYCCWPALJWVAJYAPAACWCYCAVAAJPCSAEESPZAAQVYWASEEVWADAEEJWBBSLWPCCCRARVPPSBCBECBWBLAYDJYAWZZYAADJRVSWAAAEAAJWCJCSJVCCAAAAADAADBAAAEAWDWEAYAAAADAAADAADAWDAACYAVADACADADAAADDAAADDPVAABDADAVAAAAPAAVADAEAVDWBRPSSAADA..	
	Mix12-3	..BPCWACPCCWPCVADAEEZABVBVSYWPYVJBPADAWJAEBVAPAAJAAAJEAADAACJAJBEAVPCPCADDAAEJYAJUJJOADARJEDAYBCYAYLCJAALSAABAWJDEEBLYBUPABUPJVASAAVWBSCLAWVRPEEBCVWJAQBJVWABEEWAVJABBEASJWBLWSAAASVYYABAJCUVCCACALCBSEJYSCAAAEPCCAABAJWSWBEEVYZAWYVVWAJJEVBWABEEAWBBSLBCCCWRARESVCBCAWCBPBLDYDCYAWZWADADJSVSWAASEAVYWCCCSAVCVDAAAADAADDDAAAEADWEPYAAECAQAAAWADAAAAACZDVAAAAJDAAPAAAAAAADDCVAJWBSDAAPAASEWWVAADEAAYEYRPSYAAAE..	
	Mix12-4	..BPCWAPCPCABPVYADAWZDABBBCBAVYYAPPDAEAAAEABAAWJSWADAJAVADCYJVAWECY$YWCSAAAPACAFJUPAOAADDPEAPABEVJCPCEBALCEDAJWBPBEBLYWUPASUPJWJYJACCCSWPVBARWSPBPYWSAQSVAWWAWCCAVJJBCYAJWWELPSYJVAJYYAEADPUYWCWPCLJWVAWYAPWACWCYSAVAAJPCSAEESPEAAQVYWASEWVWADAAEJWBBSLWPPCCRERVPPSSCBEVBWBLAYDJYAWZZYAADJRSSWAAAAAAJACJCSJCCCAWAAADADDBAAAEAWDAEAYEAAADADADASDAWDABCYAYADACAAADAWADDAAEDDPBAABDAWAVAYAAPAAAADAAAVDWBBPSSBADA..	
	Mix12-5	..BPCWACPCPWPCWADAEEYABVEVSYWPSVJBAADAWJDEBVJPAAJADAJEEADAACAAJBSAVPCPSADDDAEJYAPUJJOADARJVDAYYCYAYLEJAALSAABABJDEEBLYBUPABUPJVASAJVWBCCLAWVEPEEWCVWJASBJVAABEEWBVJAABEASJBBLWCAAASVAYABWJCUVCVACAACBSEJZSCABAEPCCYABAJWSWBEBVYZVWYVVWCJJEPBWABEEAWBBSLBCCSWRARESVCBSAWCEPBLDYDCYAWZWADABJSVSWAASEDVYWVCCSAVAVDAZAADAADDDAQAEADWRPYADECAQADAWAPAAAAAAZDVDAAAJDJAPAJAAAAAADCVJJWBSDJAPABSEWWVDADEDAYEYRDSYADAE..	
	Mix13-1	..BPCWACCWCWBSVAJAAEZAAVBVWYAWYVAWPAWAADAEAWAPYASYAAAAAAWACZJAAEEAJPYJCAAWAAAJAJJUPWOAVADJEDPVBCCACYCJBALSBAADWJPEEBLYWUPASUPJYAYWAVCVSCWABYRPSABCYWSAQBVDWAEECPAVJDBBWAJSWBLWSAPAAVYYARAJJUYYCAPSLCASACYSPBAACPYBAAAAJWBWAEEVPAAWBVYWAJEBVBBAAJEAWBBSLBPSCWRAREWVSCCAEEBPBLAYDCYAWZCAAADJRWSWAAAVAVJDCCCSJWCVADAAADASDDAAAABADWEPYDAEWAAYAAAPDAWAAVCZAAAAAAAEAAADAABAADDDPWAJJBAAAAABASBWAJAAAVAAAEBSPSSWAAW..	
	Mix13-2	..BPCWAWCCCCBCVDAAAEZPAVBSCCAPYCABPVADAJAEAVACWVSAAJAEACAWCCJEABESYPYPCDADADAAAAJUPJOAAJDJESPYBJVDCLCABALJEJAAWEPEEBLYWUPASUPJWAYAADCBSYPDBVRBSEBBYWSAQJVVWCADCWAVJABWYAJJWALWSVJBAVYYABAAPUYCCCPALSWAAJYPPAARWAYCAAAAJCCAAEECPZAWQVYWAAEEVVAAAEEPWBBSLEPCCARARUPWSBCWECBBBLAYDCYAWZZBAADJRVSWAAAEAPJWCACSJVCWAAAAADAADAAAABADDWEEYAAJAVAAAPAADAWAAACAAVASAAADAPAAAWDAAADDPVABBVADASAAAPPSAVAVAEADDABRPSSAAAA..	
	Mix13-3	..BPCWACWCCWYCVAJAJEZAWVBVWYVPYVDBPAWABJAEAVAPYAAAAAVEAAWAVCJABBEAJPSPCAJDAAAJAAJUPJOAVASJEDVYBCCASLCJPALSBAEAWJAEEBLYWUPAJUPJYACAAVPBSCWABVRPBEBCYWAAQBYVWAEEWWAVJABBWAPJWBLWSAPADVYYABAJJUYCCASALCASVJYSAAAACPWCAAVAJWBWAEEV$ZAWBVCWAJAEVBBAEEEAWBBSLBWCCWRAREWVRBCABCBPBLJYDCYAWZCAVADJRVSWAADEAVJWCCCSJVCVSAAAADAADDJAAABADWEPYAAEWAWAAADADAWAAACZAVAAAAEDAAJAAABAAADDYVAJJBYDAAYAASBWJVAABEAAAEPRPSAAAAW..	
	Mix13-4	..BPCWAWCWCCBSVDAAAEZPAVBSCCAWYCAWPVADADAEAWACWVSYAJAAACAWCZJEAEESYPYJCDAWADAAAJJUPWOAAJDJESPVBJVDCYCABALJEJADWEPEEBLYWUPASUPJWAYWADCVSYPDBYRBSABBYWSAQJVDWCADCPAVJDBWYAJSWALWSVJBAVYYARAAPUYYCCPSLSWAACYPPBARWAYBAAAAJCCAAEECPAAWQVYWAAEBVVAAAJEPWBBSLEPSCARARUPWSCCWEEBBBLAYDCYAWZZBAADJRWSWAAAVAPJDCACSJWCWADAAADASDAAAABADDWEEYDAJAVAYAPAPDAWAAVCAAAASAAAEAPADAWDAADDDPWABBVAAASABAPPSAJAVAVADDABSPSSWAAA..	
	Mix13-5	..BPCWACWCWWYCCAJAJEVAWVSVWYVPPVDBWAWABJBEAVAPYAAACAVEVAWAVCSABBWAJPSPJAJDSAAJAAJUPJOAVASJWDVYACCASLYJPALSBAEAJJAEEBLYWUPAJUPJYACABVPBSCWABVBPBEBCYWAAQBYVCAEEWWRVJAWBWAPJLBLWVAPADVZYABAJJUYCYASAECASVJVSAAWACPWCAAVAJWBWAEEV$ZBWBVCWYJAEWBBAEEEAWBBSLBWCYWRAREWVRBWABCWPBLJYDCYAWZCAVAAJRVSWAADEYVJWSCCSJVPVSAEAADAADDJAVABADWEPYAJEWAWAWADAAAWAAAVZAVJAAAEDBAJADABAAARDYVVJJBYDZAYAJSBWJVYABEJAAEPRCSAACAW..	
	Mix14-1	..BPCWACCACWBJVAVAAEZAAVBVZYAAYVAAPAJAAWAEAVAPDASPAAACAAJACAJAAPEASPYACAABAAAJALJUPJOAJADJEDPZBCEACFCJBALSEAAYWJPEEBLYWUPASUPJWAYPAVCSSCEABSRPSWBCYWSAQBVVWADECYAVJPBBZAJJWBLWSAYAAVYYABAJPUYJCAPBLCESAJYSPDAALPYWAAAAJWEWAEEVPBAWEVYWAJEAVBCAAEEAWBBSLBPJCWRARECVSWCAEJBPBLAYDCYAWZYAAADJRCSWAAAEAVJVCCCSJACVAAAAADAADDAAAARADWEPYCAEDAASAAACDAWAAPCZABAAAAALAAAVAADAAADDPVAJCBAVAAAJASWWABAAADAAJEBAPSSAAAR..	
	Mix14-2	..BPCWAACCCPBCVJAAAEZVAVBCCAAPYVABPBAVAJAEAVADWSSAASAEAPAECCJJABEWYPYPCAADAJAPAAJUPJOAAADJEZPYBPVPCLCJBALREPAAWSPEEBLYWUPASUPJWAYAASCBSSPJBVRASEBEYWSAQAVVWDALCWAVJABLYAJJWBLWSWJSAVYYABAVPUYCCBPALCWBAJYQPAADWWYCAAAAJACBAEEUPZAWQVYWAREEVAAAAEEYWBBSLBPCCPRARBPVSBCEECBCBLAYDCYAWZZVAADJRVSWAAAEASJWCBCSJVCSAAAAADAADDAAAYACDWEPYAACAJAAAJAADAWAAACCAVAAAAADAVAAABDAAADDPVAWBYADAWAAACPJAVAAAEAJDJBRPSSAAAA..	
	Mix14-3	..BPCWACACCWCCVAVAVEZARVBVZYAPYVABPAJAEJAEAVAPDAYAAAYEAAJAYCJAABEASPJPCAADAAAJAAJUPJOAJAAJEDJYBCEAALCJBALSEAVAWJWEEBLYWUPASUPJWAJAAVCBSCEABVRPJEBCYWSAQBSVWADEDWAVJABBZABJWBLWSAYAWVYYABAJPUYCCAJALCESPJYSBAAALPACAAAAJWEWAEEVBZAWEVYWAJPEVBCAAEEAWBBSLBPCCWRARECVWBCAECBPBLAYDCYAWZYAAADJRVSWAAEEAVJWCCCSJVCVVAAAADAADDSAAARADWEPYAAEDADAAAVADAWAAACZAVAAAARDAASAAADAAADDJVAJCBVDAAAAASWWSVAAJEAAJEVRPSRAAAR..	
	Mix14-4	..BPCWAACACPBJVJAAAEZVAVBCCAAAYVAAPBAVAWAEAVADWSSPASACAPAECAJJAPEWYPYACAABAJAPALJUPJOAAADJEZPZBPVPCFCJBALREPAYWSPEEBLYWUPASUPJWAYPASCSSSPJBSRASWBEYWSAQAVVWDALCYAVJPBLYAJJWBLWSWJSAVYYABAVPUYJCBPBLCWBAJYQPDADWWYWAAAAJACBAEEUPBAWQVYWAREAVAAAAEEYWBBSLBPJCPRARBPVSWCEEJBCBLAYDCYAWZZVAADJRCSWAAAEASJVCBCSJACSAAAAADAADDAAAYACDWEPYCACAJASAJACDAWAAPCCABAAAAALAVAVABDAAADDPVAWBYAVAWAJACPJABAAADAJDJBAPSSAAAA..	
	Mix14-5	..BPCWACCJCWBBVABAAEZAAVBVEYABYVASPASAASAEAVAPJASJAAAPAALACDJAAAEACPYPCAAJAAAJAWJUPJOAPADJEDPBBCBACJCJBALSEAABWJPEEBLYWUPASUPJWAYCAVCBSCBABDRPSBBCYWSAQBVVWAAECZAVJABBSAJJWBLWSACAAVYYABAJPUYSCAPWLCWSAJYSPVAAPPYYAAAAJWCWAEEVPWAWQVYWAJELVBVAAEEAWBBSLBPVCWRAREAVSECAEWBPBLAYDCYAWZVAAADJRVSWAAAEAVJPCCCSJBCVAAAAADAADDAAAAYADWEPYVAEVAAAAAAWDAWAAJCZADAAAAARAAALAADAAADDPVAJLBACAAACASAWAWAAACAADEBVPSSAAAA..	
	Mix15-1	..BPCWACCJCWBBVABAAEZAAVBVEYABYVASPASAASAEAVAPJASJAAAPAALACDJAAAEACPYPCAAJAAAJAWJUPJOAPADJEDPBBCBACJCJBALSEAABWJPEEBLYWUPASUPJWAYCAVCBSCBABDRPSBBCYWSAQBVVWAAECZAVJABBSAJJWBLWSACAAVYYABAJPUYSCAPWLCWSAJYSPVAAPPYYAAAAJWCWAEEVPWAWQVYWAJELVBVAAEEAWBBSLBPVCWRAREAVSECAEWBPBLAYDCYAWZVAAADJRVSWAAAEAVJPCCCSJBCVAAAAADAADDAAAAYADWEPYVAEVAAAAAAWDAWAAJCZADAAAAARAAALAADAAADDPVAJLBACAAACASAWAWAAACAADEBVPSSAAAA..	
	Mix15-2	..BPCWAJCCCJBCVVAAAEZSAVBPCSAPYVABPZAAAJAEAVAQWPSAABAEAAAJCCJCABEVYPYPCAADAAASAAJUPJOAAADJEDPYBBVQCLCJBALZEYAAWPPEEBLYWUPASUPJWAYAAWCBSZPBBVRCSEBCYWSAQCVVWSAACWAVJABBYAJJWBLWSAJEAVYYABAJPUYCCSPALCWCAJYJPAAWWPYCAAAAJBCWAEEEPZAWQVYWABEEVJAAAEEAWBBSLBPCCWRARLPVSBCAECBSBLAYDCYAWZZZAADJRVSWAAAEAWJWCWCSJVCRAAAAADAADDAAAWABDWEPYAAVARAAAAAADAWAAACLAVAAAAADAJAAAJDAAADDPVAEBSADAPAAARPBAVAAAEASDVBRPSSAAAA..	
	Mix15-3	..BPCWACJCCWACVABALEZACVBVEYAPYVABPASAPJAEAVAPJAZAAADEAALAJCJAABEACPYPCAADAAAJAAJUPJOAPABJEDWYBCBAVLCJBALSEAWAWJPEEBLYWUPASUPJWABAAVCBSCBABVRPAEBCYWSAQBCVWAAELWAVJABBSAYJWBLWSACABVYYABAJPUYCCAWALCWSSJYSJAAAPPJCAAAAJWCWAEEVIZAWQVYWAJBEVBVAAEEAWBBSLBPCCWRAREAVBBCAECBPBLAYDCYAWZVAAADJRVSWAAAEAVJWCCCSJVCVRAAAADAADDPAAAYADWEPYAAEVACAAAAADAWAAACZAVAAAADDAAQAAADAAADDLVAJLBDDAAAAASAWDVAAAEAADEWRPSVAAAA..	
	Mix15-4	..BPCWAJCJCJBBVVAAAEZSAVBPCSABYVASPZAAASAEAVAQWPSJABAPAAAJCDJCAAEVYPYPCAAJAAASAWJUPJOAAADJEDPBBBVQCJCJBALZEYABWPPEEBLYWUPASUPJWAYCAWCBSZPBBDRCSBBCYWSAQCVVWSAACZAVJABBYAJJWBLWSAJEAVYYABAJPUYSCSPWLCWCAJYJPVAWWPYYAAAAJBCWAEEEPWAWQVYWABELVJAAAEEAWBBSLBPVCWRARLPVSECAEWBSBLAYDCYAWZZZAADJRVSWAAAEAWJPCWCSJBCRAAAAADAADDAAAWABDWEPYVAVARAAAAAWDAWAAJCLADAAAAARAJALAJDAAADDPVAEBSACAPACARPBAWAAACASDVBVPSSAAAA..	
	Mix15-5	..BPCWACJCJWACYABALECACVBVEYAPBVABJASAPJAEAVAPJAZAVADEBALAJCDAABDACPYPWAADAAAJAAJUPJOAPABJEDWYCCBAVLAJBALSEAWASJPEEBLYWUPASUPJWABAEVCBSCBABVRPAEBCYWSAQBCVJAAELWWVJADBSAYJWBLWWACABVYYABAJPUYCCAWALCWSSJSSJAAAPPJCAAAAJWCWAEEVIZDWQVYWSJBEYBVAAEEAWBBSLBPCVWRAREAVBBVAECBPBLAYDCYAWZVAAADJRVSWAAAEBVJWCCCSJVCVRAAAADAADDPASAYADWEPYAZEVACAPAAASAWAAAJZAVAAAADDEAQABADAAADDLVAJLBDDYAAAESAWDVJAAEBADEWRZSVAAAA..	


Conformation Search 
Each conformation from PFVM

D	PFVM-01	..BPCWACCCCWBCVAAAAEZAAVBVCYAPYVABPAAAAJAEAVAPWASAAAAEAAAACCJAABEAYPYPCAADAAAJAAJUPJOAAADJEDPYBCVACLCJBALSEAAAWJPEEBLYWUPASUPJWAYAAVCBSCPABVRPSEBCYWSAQBVVWAAECWAVJABBYAJJWBLWSAJAAVYYABAJPUYCCAPALCWSAJYSPAAAWPYCAAAAJWCWAEEVPZAWQVYWAJEEVBAAAEEAWBBSLBPCCWRAREPVSBCAECBPBLAYDCYAWZZAAADJRVSWAAAEAVJWCCCSJVCVAAAAADAADDAAAAAADWEPYAAEAAAAAAAADAWAAACZAVAAAAADAAAAAADAAADDPVAJBBADAAAAASPWAVAAAEAADEBRPSSAAAA..
   1. AADAADADQSWSVAAAAAAAAAAAJWYJVAAAADAAAAAADAAAAJVAAAAAAAAAADAAAAJVJVAADAAAADAAAAJVJVAAAAAAAAAAAAAAAPYJVAAADAAAAAAAAAAADJVAADAAPSVADAAAAAAAPYJVADADAAADAADDDAAAPCSVJWSBWCYAAAAAAAAAAAAPCZAAAADAAAAAAAJEBVPYAADAAAAADAAAAAAJVJVAAAAAAAADAADAAAAPSVAPYAADAAAAAAAAADAAAAAPCYAAAADAAAAAAAAAADJVAAAAAJVAAAPSWYAPCYAAAAAAAAAAADAJWYAAAAADDADAAAAAAPYJVAAAAAAAAAAAAADAAJVAAAAAAAAAAAAAAAJBWYAAAADAAAAAAAAADDJVAAAAAAAAAAA : 6D83 : B :   35 :  433 : 0.476
   2. AAADAAAPSVAAAADDAAADDAAAAAJVAJVJVAAAAAADAAAJVAAAAAAAADADAAAAAAJBVAJBWCYDAAAAAAAAAAAAAAAAAAAAAAAAAQSVAJVAAAAAAAAAAAAAAAJVAPSVAAAAAJVAAAAAAAAAAJVAPCYAAAAAAAAAAAADDAAJVJVADJVAAAADAAAAAAAAAAAAAAAAAPSBVAAAAAAAADDAAADADAAAAADDJVAP$ZJBVAAAAAAAAAAAJVJVAAAAAAAAAAAAAADDAAAJVJVAAAAAAAAAAADDAAAAAAAAJWCYJBWCCCSVAPZAAAAAAAAAAAAADAAADPCYADAAAAAAAAAAAPSWCYAAAAAAAAAAADAAAAPSVAJVADAAAAAAAAAAJWYAPCYAAAAAAAAAAAAAAAA : 6GX9 : A :   93 :  491 : 0.476
   3. DAAADDAPCSBVAAAAAAAAAAAAAAAAAAAAAAAAAAAAAAAAADAADDAAAADAAAAAAAAADAADAAAAAAAAAAAAAAAAAAAAAAAAAAAAAAAAAAAAAAAAAAAAAAAAAAAAAAAAAAAAAAAAAAAAAAAAAAAAAAAAAAADAADAAAAAADAAAAAADAAAAAAADAAAAAAAAAADAAAAAAJBWYAAAAAAAAAADAAADDAPYJVJBWYDAAADAADAAAAAAAAAADAAADAADDADAAAAAAAAAAAAAAAAADDPYJWCYAAADAAAAAAAAAADAADDAAAAAAAAAAAAAAAAAAAAAAAADAAAAADAAADQCCSWSVDJVAAAAAAAAAAAAAADDAADAAADAAAAAAAAAAAAAAAAAAAADAAAAAAAAAAAAAA : 7NYX : B :  215 :  613 : 0.476
   4. AAADAAAPSVAAAAAAAAADDAAAAAJVAJVJVAAAAAADAAAJVAAAAAAAADADAAAAAAJBVAJBWCYAAAAAADAAAAAAAAAAAAAAAAADAQSVAJVAAAAAAAAAAAAAAAJVAPSVAAAAAJVAADAAAAAAAJVAPCYAAAADDAAAAAADDAAJVJVADJVDAAADDDAAAAAAAAAAAAAAAPSBVAAAAAAAADDAAADADAAAAADDJVAP$ZJBVAAAAAAAAAAAJVJVAAAAAAAAAAAAAAADAAAJVJVAAAAAAAAAAAADAAAAAAAAJWCYJBWCCCSVAPZAAAADAAAAAAAADAAAAPCYADAAAADAAAAAAPSWCYADAAAAAAAAADAAAAPSVAJVADAAAADAAAAAJWYAPCYAAAAAAAAAAAAAAAA : 6GX9 : B :   93 :  491 : 0.474
   5. SWYQSWCCCCSVAAAAAAAAAAAAPYJVAAAAAAAAAAAAAAAAAAADJVJVAAAAAPYAAAAAADAAAAAAAPYJVAAAAAAAAAAADAADAPCYADAADAAAAAAAAAAAADAAAAAPSBWYAAAAAAAAAAAAAAAAAPCFCYDAJWYAAAAAAAAAAADAAAAAAAAAJWYJVAAAAAADAAAAAAADDPYPCYAADAAAAAAAAADAAJWCSWSVAAPCYAAAAAAAAAAADAAAPYPSVJLSBVAPYAAAAAAAAAAAADAPCYAAAAAPYAAAAJVJVAAAAAAAAAAAAAJVAJVAAAAAAAAAAAAAAJBVJVAAAAAAAAAAAAAAADJEEWYAAAAAAAAADAAAAAJVAAJWZAAAAAAAAAAADJVAAJWYAAAAAAAAAAADAAA : 7KTR : A :  1077 :  1475 : 0.471
   6. DAAADDAPCSBVAAAAAAAAAAAAAAAAAAAAAAAAAAAAAAAAADAADDAAAADAAAAAAAAADAADAAAAAAAAAAAAAAAAAAAAAAAAAAAAAADDAADADDDAAADAADAAADAAAAAAAAAAAADADAADADAAAAADDAADDDDDDDDDDDDDDDAAAAAADAAAAAAADAAAAAAAAAADAAAAAAJBWYAAAAAAAAAADAAADDAPYJVJBWYDAAADAADAAAAAAAAAADAAADAADDADAAAAAAAAAAAAAAAAADDPYJWCYAAADAAAAAAAAAADAADDAAAAAAAAAAAAAAAAAAAAAAAADAAAAADAAADQCCSWSVDJVAAAAAAAAAAAAAADDAADAAADAAAAAAAAAAAAAAAAAAAADAAAAAAAAAAAAAA : 7NYZ : B :  215 :  613 : 0.470
   7. JBVAJVAAJEWYAAAAAADJBWYPSVPYAJBWYAAAAAAAQYAAAAAADAADAADDAADAAADAAAAAAADJVJVAADAAAAAAAAAAAAAAAAAAAAAAAAAAAAAAAAAAAAAAAAAAAAAAAAAAAAAAAAAAAAAAAAAAAAAAAAAAAAAAAAAAAAAAAAAAAAAAAAAAAAAAAAAAAAADAAADAAAAAAAAAAAAAAAAAAAAAAADAAAAAADAAADAADAAAAAAAAAAAAAAAAAAAAJWSVADAAAAAAAAADDAADJWYAAAAAAAAAAADAAAAAADADDAAAAAAADAAAADDAAAAAAAAAAADAAAAAAADAAAAAAAAADAAAADAAAAAAAAAADAAAAADAADAAADDAAAAAADAAAADAAAADDDAAAJVAJVJBB : 6YVU : B :  291 :  689 : 0.470
   8. ADDAADAAAAAAAAAAAAAAAAADAAAAAAAAAAAAAJVAAAAAJVPCYAADAAAAAAAAAADAAAAADAAADAAAAAAADAAAAAAADAAJVJWZAJWYAAAAAAADDAAAAAAAPSVAAADAAAADAADAAAAAAAAADDAADAJVJVAAAADAAAAADAAAAAAAAAADDAAAAAAAAAAAAAAAAPSBBVJWCYAAAAAADADDAADAAAADAAAAAAADAADAAAAAAAAAAAAAAAAAAAAAAAAAAAAAAAJBVAAAAADDDAAAAAAAADADDAAAAAAAADAAAAAJWSVJVAAAAAAAAAAAAADAAAAAAAAAAAAAAAAAAADAAAAAADAAAAAAAAAJBWYAAAAAADAADAAAAAAAAAAAAAAAAAAAAAAAAAAAAAAAAAA : 6D05 : E :  219 :  617 : 0.469
   9. ADDAADAAAAAAAAAAAAAAAAADAAAAAAAAAAAAAJVAAAAAJVPCYAADAAAAAAAAAADAAAAADAAADAAAAAAADAAAAAAADAAJVJWZAJWYAAAAAAADDAAAAAAAPSVAAADAAAADAADAAAAAAAAADDAADAJVJVAAAADAAAAADAAAAAAAAAADDAAAAAAAAAAAAAAAAPSBBVJWCYAAAAAADADDAADAAAADAAAAAAADAADAAAAAAAAAAAAAAAAAAAAAAAAAAAAAAAJBVAAAAADDDAAAAAAAADADDAAAAAAAADAAAAAJWSVJVAAAAAAAAAAAAADAAAAAAAAAAAAAAAAAAADAAAAAADAAAAAAAAAJBWYAAAAAADAADAAAAAAAAAAAAAAAAAAAAAAAAAAAAAAAAAA : 6D05 : F :  219 :  617 : 0.469
  10. ADDADAAQSWSVAAAAAAAAAAAJBVJVADAAAAAAAAADAAADJVAAAAAAAAAAAAAAJWYJVAAAAAADAAAAAJVJVAAADAAADAAAAAAJWYJVAAAAAAAAAAAAAAAQSVAAAAAPSVAAAAAAAAJBVJVAAADAAADDAAAAAADJWYPSBVJLCZAAAAAAAAAADAPCZAAAAAAAAAAAAJWSWCYAAAADDAADAAAAAAJVJVAAAAAAAADADDAAAAPSVAPYAAAAAADAAAAAAAAAAAPCYAAADDAADAAAAAADDJVADAAAAAAAAPSWYAPCYAAADAAAAAAAAAJWYAAAADAAADAAAAAAJVJVAAAAAADAAAAAAAAAJVAAADDAAADAAAAAAJBBVAAAAAAAAAAAAAADDJVAAAPYAAAAAAA : 4P6Z : B :   36 :  434 : 0.469	
	Coup	..BPCWACCCCCYPCYAAAEVAAVBVCYAPYVAPYAAAAJBEBVAPYAJWAAAJEVAAAAJVAPSWJPYPCSADAAAJAAJUPJOAAADJEDPYAJVAAPCJBALSEVAAJBWEEBLYWUPAJUPJWAJWADPCSWPYBVRWJEBBVWJAQSVAAAADDPRVJAABEAJWWALWSVJVAJYYABWJPUYJVAPCSCWAAJVAPYAAPCYAAAAAJWCSBEEVPZAAQVYWAJEEVAAAAAEAWBBSLBWPCCREREWPSBWAEVBWBLAYDJYAWZPYAADJRVSWAAAAAAJWCCCSJVAAAAAAADAADDAAAAAADAEEYAAAAAAAAAAADAAAAAAAAAAAAAADAAAAAADAAADDPVAJBBVDAAAAAAPSWAAAAAAADAPRBSVAAAA..
   1. VAJVAAJEWYAAAAAADJBWYPSVPYAJBWYAAAAAAAQYAAAAAADAADAADDAADAAADAAAAAAADJVJVAADAAAAAAAAAAAAAAAAAAAAAAAAAAAAAAAAAAAAAAAAAAAAAAAAAAAAAAAAAAAAAAAAAAAAAAAAAAAAAAAAAAAAAAAAAAAAAAAAAAAAAAAAAAAAADAAADAAAAAAAAAAAAAAAAAAAAAAADAAAAAADAAADAADAAAAAAAAAAAAAAAAAAAAJWSVADAAAAAAAAADDAADJWYAAAAAAAAAAADAAAAAADADDAAAAAAADAAAADDAAAAAAAAAAADAAAAAAADAAAAAAAAADAAAADAAAAAAAAAADAAAAADAADAAADDAAAAAADAAAADAAAADDDAAAJVAJVJ : 
6YVU: B :  293 :  687 : 0.516
   2. DAAAAAAAAAAAAAAAAADAAAAAAAAAAAAAJVAAAAAJVPCYAADAAAAAAAAAADAAAAADAAADAAAAAAADAAAAAAADAAJVJWZAJWYAAAAAAADDAAAAAAAPSVAAADAAAADAADAAAAAAAAADDAADAJVJVAAAADAAAAADAAAAAAAAAADDAAAAAAAAAAAAAAAAPSBBVJWCYAAAAAADADDAADAAAADAAAAAAADAADAAAAAAAAAAAAAAAAAAAAAAAAAAAAAAAJBVAAAAADDDAAAAAAAADADDAAAAAAAADAAAAAJWSVJVAAAAAAAAAAAAADAAAAAAAAAAAAAAAAAAADAAAAAADAAAAAAAAAJBWYAAAAAADAADAAAAAAAAAAAAAAAAAAAAAAAAAAAAAAAAAAA : 6D05 : E :  224 :  618 : 0.515
   3. DAAAAAAAAAAAAAAAAADAAAAAAAAAAAAAJVAAAAAJVPCYAADAAAAAAAAAADAAAAADAAADAAAAAAADAAAAAAADAAJVJWZAJWYAAAAAAADDAAAAAAAPSVAAADAAAADAADAAAAAAAAADDAADAJVJVAAAADAAAAADAAAAAAAAAADDAAAAAAAAAAAAAAAAPSBBVJWCYAAAAAADADDAADAAAADAAAAAAADAADAAAAAAAAAAAAAAAAAAAAAAAAAAAAAAAJBVAAAAADDDAAAAAAAADADDAAAAAAAADAAAAAJWSVJVAAAAAAAAAAAAADAAAAAAAAAAAAAAAAAAADAAAAAADAAAAAAAAAJBWYAAAAAADAADAAAAAAAAAAAAAAAAAAAAAAAAAAAAAAAAAAA : 6D05 : F :  224 :  618 : 0.515
   4. AADDAPCSBVAAAAAAAAAAAAAAAAAAAAAAAAAAAAAAAAADAADDAAAADAAAAAAAAADAADAAAAAAAAAAAAAAAAAAAAAAAAAAAAAAAAAAAAAAAAAAAAAAAAAAAAAAAAAAAAAAAAAAAAAAAAAAAAAAAAAAADAADAAAAAADAAAAAADAAAAAAADAAAAAAAAAADAAAAAAJBWYAAAAAAAAAADAAADDAPYJVJBWYDAAADAADAAAAAAAAAADAAADAADDADAAAAAAAAAAAAAAAAADDPYJWCYAAADAAAAAAAAAADAADDAAAAAAAAAAAAAAAAAAAAAAAADAAAAADAAADQCCSWSVDJVAAAAAAAAAAAAAADDAADAAADAAAAAAAAAAAAAAAAAAAADAAAAAAAAAAAA : 7NYX : B :  217 :  611 : 0.513
   5. AAAAAAAQSBVADAAAAAAAADAJVJBVPCCCCYAAAAAADAAAAAAAAAPSBVADAAAADAAAAJVPYJWSVAAAAAJVAAADDAADADAADAAAAAAQSWSBVADAAAADAAAAJVDDADAAADDAAAAAAADAAAQSBVAAAAAAAAAAAAAAADAAAAPCYAJVAAAAAAAAAAADAAAAAAAAAAAAJVAAAAPYAAAAAAAAAADAJWCSVDAPCYAAAADDAAAAAAAAAAAAAAAPCYPYPYAAAAAAJVAAAAAADDAAAPCCZAAAAAAAAAAAAAAAAAAAAAQSBVAAAADAAADAADAAAAPYAAAAAAAAAAAAAAAAAAAAPCZDAAAADAADAAAAAAJVADAAAAAAJVAAADAAAAAAAAAAAADDAAAAAJVAAAA : 7TYR : A :  2955 :  3349 : 0.513
   6. ----..SBWSWSVPSVAAAAAAAAAAAAAAAAAAADAAAAAAAAAADAJVAAAAAAADAAAAAAPZAAAAAAADAAAAAAADAAAAAAAAAAAAAAAAAAAAAAAAAAADDJVJVADAAAAAAAAAAAAAAAAAAAAAADAAAAAAAAAAAAAAAAAAAAAAAAAAAADAAQSVAAAAAAPYJWSWSVAAADDAAAAAAAPYPYAPCSWYAJWYAAJVDAAAAAADAADAAAAAAAAAAJWSBBVQYJBBVJVADAAAAAAAADAAAAAAAAAAAAAAAAAAAAAAAAAAAAAAAAAADAAAAAADJBVAAAAAAAAAAAAAAADAAAAAAAAAAADDAAAAAAAAAAAAAAAAADAAAAAAPYAJWYAAADAAADAAAAAAAAAAAAAAAAAAA : 6TQF : A :    1 :  395 : 0.510
   7. ----..SBWSWSVPSVAAAAAAAAAAAAAAAAAAADAAAAAAAAAADAJVAAAAAAADAAAAAAPZAAAAAAADAAAAAAADAAAAAAAAAAAAAAAAAAAAAAAAAAADDJVJVADAAAAAAAAAAAAAAAAAAAAAADAAAAAAAAAAAAAAAAAAAAAAAAAAAADAAQSVAAAAAAPYJWSWSVAAADDAAAAAAAPYPYAPCSWYAJWYAAJVDAAAAAADAADAAAAAAAAAAJWSBBVQYJBBVJVADAAAAAAAADAAAAAAAAAAAAAAAAAAAAAAAAAAAAAAAAAADAAAAAADJBVAAAAAAAAAAAAAAADAAAAAAAAAAADDAAAAAAAAAAAAAAAAADAAAAAAPYAJWYAAADAAADAAAAAAAAAAAAAAAAAAA : 6TQF : B :    1 :  395 : 0.510
   8. AAJBVDDAAAAAAAAADAAAAPCYAAAAAAAAAAAAAADDAAAPYJBVAAAAADDAAAAAAAAAJWYPSVQSVDAAAAAAAAAAADAJWYAAAAAAAAAAAAADDAAAAAAAJBVAAJWZAAAAAAAAAAAAAAAAAAAAAADJVAAAAAAAAAAAADAAAAAAPYJVADAAAAAAADAAAAAADJVDAAAAAADDAAAAAAAAAAJVAPYAAAAAAAAAAAAADAJVDAAAAAAAAAAADAAAAAAJVAPCYAAAAAAAAAAAADAADJVAAAAAAAADAAAAAAAAAAJWCCCYPYAAAAAAAAAAAAAAAAAADAAADAAAJVADAAAAAAAAAAAAAAAAAJVADAAJVAAAADDAAAADAAAAAJVPSVAAAAAAJVADDAAAAADDAJV : 5OWU : A :  444 :  838 : 0.510
   9. APCYAAAAAAAAAAAAAAAQSWSVAAAAAAAAAAAAAAAJVAAAAAAADAAAADAAJVAAAAJVAAAAAAAAAAAAAAAAAAAAAAPSBVADAAAAAADDAADADDAAAAAAAJVAAAAAAAAAAAAAAAAAJWYPCYDDAJVAAAAAAAAAAAAAAAAAAPCYDDAADDADDAAAAAAAAADAAAAAAPCCCZAAAAAPCYAAAAAAAAAAADAADAAAAAAADJVDAADADAAADDDADDDAJBWCSBVADAAADAAAAADAAAJWCYAJVAAAADAAAAAAAPSVAJVAAAAAAAAAAAAAAAAAADJVAAAADAAAAAAAAAAJVAAADDDAAAAAAAADDJVAAAADAAAADAAAAAADAAPSVPCZAAAAAAAAAAAAAAAAPCYAAAA : 2X19 : B :  213 :  607 : 0.509
  10. AAAAAAAAQYJVADAAAAAAAAAAAJVPYAPSVAAAAAAAAAAAAQSBVAAAAAAAAAAAAQSBVJVAAAAAAAAAAADPSBVADAAAAAAAAAAAJVAJVAAAAAAAAAAADQYJVAAAAAAAAAAAADJVAAJWYAAAAAAAAAAAAAAAAAAAAJWYJVAAAAAAAAAAADAAAAJVJVADAAAAAAAAAAAPYJVAAAAAAAAAAAADJVAJVAAAAAAAAAJVAAAJVAAAAAAAADAAADJVAJVAADAAAAAAJVJVAAAAAAAAADAAAADDJBBVAAAADAAAAADDJVADAAAAAAAAAAAAAAAJBVDDJVAAADAAAAAAAAAAAAQSBVAAAAADAAAAADQSVAJVAAAAAAAAAAAAQYJVAAAAAAAAAAAAAJVAJVA : 6HD5 : t :   59 :  453 : 0.508	
	Mix12-1	..BPCWACCPCWBPVADAAWZAABBVSYAVYVAPPADAAAAEABAPAASWAAAJAADACYJAAWEAVPYWCAAAAAEJAFJUPAOADADPEDPABCYACPCJBALSAAAJWJPBEBLYWUPASUPJVAYJAVCCSCLABARPSPBCVWSAQBVAWABECCAVJJBBEAJWWBLPSAAAAJYYAEAJCUYWCAPCLCBSAWYSPWAAEPYSAAAAJWSWAEEVPEAWYVYWAJEWVBWAAAEAWBBSLBPPCWRERESVSSCAEVBPBLAYDCYAWZWAAADJRSSWAAAAAVJACCCSJCCVAWAAADADDDAAAAEADAEPYEAECAADAAASDAAAABCZAYAAAAAAAAAWAAAAAEDDPBAJWBAWAAAYASEWAAAAAAAAYEBBPSSBAAE..
   1. VPSWCYAAAAAAAAAAAAAAAQYPRBVAAAAAAAAAAAJVJVAAAAAAAAAAAAJEBVJVAAAAAAAAAAAAAAAJVJVAAAAAAAAAADDJBWYAAADDAADAAAAAAAJVAPCYAAADAAAAAAADAADDJVAAJWSWYAAAAAAAAAAJVJVAAAADAADDAADAADDJVADAAAAAAAAAAAAAADDAPCZAAJVAAJW$YPCSVAAAAAAAAAAAAPCCSVJVAAAAAAAAAAAAAAAAAAPCSVJVJVAAAADAAADAAAAAAAAADJVAJVAAAAAAAAAAAAAAAJVJVAAADAAAAAAAAAAAJVAAAAAAAAAAAAAAAAADAJVJVAAAAAAAAAAAAAJWYDAAAAAAAAAAAAADAPCYAAAAAAAAAAAAAADDJBVJVAA : 7RW8 : A :   47 :  441 : 0.457
   2. AAAADDDAAAAAAADDDADAADADDAAAAADAAADAAAAAADAAAAAADAAAAAAAAAAAADDADAAAAADAAAAAAAAAAAAAAAAADDDAADAAAAADDAAAADAAAAAAAAAAADAAAAAAAADAAAAAADAAAAAADAAAAAADAAAADAADADAAADDAAAADDAAAAADAAAAAADDDDAAAAAAADAAAAAAAADAAAADAAAAAADDAADDADAAAAAAAAADDAADAAAAAAAAAADAAADAADAAAAAAAAADAADAAAAAAADDAAAAADADDADAAAAAAADDDAAAADDAAAAAADDAAADAAAADDAADADAAADAAAAAADDAADAAAAAADDADADDADAAAADDDAADDAAAAAADDAAAAAAADDAADDAAAAAAAA : 7KOG : A :  1464 :  1858 : 0.456
   3. ADAAAADADDDJVAJVADAAAADAAAAAAADAAAAAADAAAAAAAAJVJVAAAADDDAAADDDDAAAAAAADAADAAAAAPCSVAAAAAAAAAAAAAAAAADADDAAAJVJBEWCYAJBVDAAADAAADAAAAAAAAADAAAAAADJVJBVAAAAAAAAAAAAAADAADADAAAAAAJVJVAAAAAAAADAAAAADDAAAAADAADDAAAJVJBVAAAAAAADAADAADDDAAAADAAJVAJWSVDDDDADDAAAAAAAAQSBWSVJBWCYAAAADAAADAAAAADADAAAADAAAAPCYAAAAAAAADDAAAADDAADAAAAAAAAJVJVADAAADADDAAAAAAAAADDDAAAAAADDJVAJVAAAAADADAAAAAAAAAAAAAADAPSVAJV : 6R9T : A :  965 :  1359 : 0.456
   4. ----..AAAJVAAPZAAAAAAAAAJVJVAAAAAAAAAAAAAAAQSWSVAAAAAAAAAAAAJVJVAAAAAAAAAAAAAADQSVAAAAAAAAAAAAAAAJVJVAAAAAADAAAAAJVPYAAAAAAADAADAAAJBVJVAAAAAADAAAAAAAAPYPSBVAAAAAAAAAAAAAAPYJVAAAAAAAAAAAAAAAJEWYAAJWSBVADAAAAAADAAAAPCZAAAADDAAAAAAJEBVJVAAAAAAAADAAAAJWYJWZADAAAAAADAAAAAAPSVDAJVAADAAADAAAAAAAAAAAPCYDAAADAAAAAADAAADJVAAAAAAAAAAPSWYAPCYAAADAAAAAAAAAJWYAAAADAAADAAAAAAPYJVAAAAAADAAAAAAAAAJVAAAAAAAAA : 7Z5C : B :    4 :  398 : 0.454
   5. AAADDDADDDDAADDAADAAAAAAAAAAAAAAADDADAAAAAAAAAAAAAAAAAAAAAAAAAJBBBBVADAAAADAAAAAADAAAAAAAAAAAAAAAAAAAAAAAAAAAAAAAAAPSVJBBWSBBVAJVAJBWCCCYAAADDAADDAAAAADAAAAAAAAAJBBBWYAAAAAAADADAAAADAAAAADAAPCYAAAAAAAAAAAAAAAAAAAAAAAAAAAAAAJWYAJWSBBEVJVAJBBBW$CSVAAAAJBWCCCCYADAAAAAAAAAAAAAAAAAAAAAAAAAAAAAAAAAAAAAAAAAAPSWYAADAAAAAAADAAAAAAAAAAAAADAAAAAAAAADDAAAAAAAAAAADAADADDAAAAAAADAAAAAAAADDAAAAAAAAADAAAAAAA : 8AT3 : D :   55 :  449 : 0.454
   6. ADAAAPSVAAAADDAAADDAAAAAJVAJVJVAAAAAADAAAJVAAAAAAAADADAAAAAAJBVAJBWCYDAAAAAAAAAAAAAAAAAAAAAAAAAQSVAJVAAAAAAAAAAAAAAAJVAPSVAAAAAJVAAAAAAAAAAJVAPCYAAAAAAAAAAAADDAAJVJVADJVAAAADAAAAAAAAAAAAAAAAAPSBVAAAAAAAADDAAADADAAAAADDJVAP$ZJBVAAAAAAAAAAAJVJVAAAAAAAAAAAAAADDAAAJVJVAAAAAAAAAAADDAAAAAAAAJWCYJBWCCCSVAPZAAAAAAAAAAAAADAAADPCYADAAAAAAAAAAAPSWCYAAAAAAAAAAADAAAAPSVAJVADAAAAAAAAAAJWYAPCYAAAAAAAAAAAAAA : 6GX9 : A :   95 :  489 : 0.454
   7. AAAAADAADDAAAADAAAAAAAAADAADAAAAAAAAAAAAAAAAAAAAAAAAAAAAAAAAAAAAAAAAAAAAAAAAAAAAAAAAAAAAAAAAAAAAAAAAAAAAAAAAAAADAADAAAAAADAAAAAADAAAAAAADAAAAAAAAAADAAAAAAJBWYAAAAAAAAAADAAADDAPYJVJBWYDAAADAADAAAAAAAAAADAAADAADDADAAAAAAAAAAAAAAAAADDPYJWCYAAADAAAAAAAAAADAADDAAAAAAAAAAAAAAAAAAAAAAAADAAAAADAAADQCCSWSVDJVAAAAAAAAAAAAAADDAADAAADAAAAAAAAAAAAAAAAAAAADAAAAAAAAAAAAAADAAAPYJEBVPYAAAAAAAAAAAAAAAAAADAAAAA : 7NYX : B :  255 :  649 : 0.454
   8. DADAAQSWSVAAAAAAAAAAAJBVJVADAAAAAAAAADAAADJVAAAAAAAAAAAAAAJWYJVAAAAAADAAAAAJVJVAAADAAADAAAAAAJWYJVAAAAAAAAAAAAAAAQSVAAAAAPSVAAAAAAAAJBVJVAAADAAADDAAAAAADJWYPSBVJLCZAAAAAAAAAADAPCZAAAAAAAAAAAAJWSWCYAAAADDAADAAAAAAJVJVAAAAAAAADADDAAAAPSVAPYAAAAAADAAAAAAAAAAAPCYAAADDAADAAAAAADDJVADAAAAAAAAPSWYAPCYAAADAAAAAAAAAJWYAAAADAAADAAAAAAJVJVAAAAAADAAAAAAAAAJVAAADDAAADAAAAAAJBBVAAAAAAAAAAAAAADDJVAAAPYAAAAA : 4P6Z : B :   38 :  432 : 0.454
   9. JVJBVPCCCCYAAAAAADAAAAAAAAAPSBVADAAAADAAAAJVPYJWSVAAAAAJVAAADDAADADAADAAAAAAQSWSBVADAAAADAAAAJVDDADAAADDAAAAAAADAAAQSBVAAAAAAAAAAAAAAADAAAAPCYAJVAAAAAAAAAAADAAAAAAAAAAAAJVAAAAPYAAAAAAAAAADAJWCSVDAPCYAAAADDAAAAAAAAAAAAAAAPCYPYPYAAAAAAJVAAAAAADDAAAPCCZAAAAAAAAAAAAAAAAAAAAAQSBVAAAADAAADAADAAAAPYAAAAAAAAAAAAAAAAAAAAPCZDAAAADAADAAAAAAJVADAAAAAAJVAAADAAAAAAAAAAAADDAAAAAJVAAAAAPCYADAAAAAAAJBBVJVPYAA : 7TYR : A :  2978 :  3372 : 0.454
  10. ADJWSBBBBVAADAAAAAADAAAAAAAADDAAAAAADAADAAAAAAJWYAAADAAAAAAAAAAAAAAAAADDAAAAAAAAAAAAAAAAADDDDAAAJVPSVAAAAAAAAAAAADAAADAADAAADAAAAAAAAAAAAAAAAAAAADAAAAAADAAAAAAAJBBBBWYDAADAAAADAAAAAAAAAAAAAAAAAAAAAAAAAAAAAAAAAAAAAAAAAAAAAAAAADAAAAAAAAAAADAAAQZAAQZAAJBVAAAAPCYAAAAAAAAAAAAAAADAAAAJVDAAAAAAAAAJVAJVAAAAAADAADDDAAAAAJBVADJVJVJVAAAAAAAAAAADAAADAAAAAAAAAAAAAAAAAAAAAAAAAAAAAAAAAAAAAAAJBBW$YJVJEBBBEWC : 7NYZ : A :  885 :  1279 : 0.453	
	Mix12-2	..BPCWAPCCCABCVYADAEZDAVBBCBAPYYABPDAEAJAEAVAAWJSAADAEAVADCCJVABECY$YPCSADAPACAAJUPJOAADDJEAPYBEVJCLCEBALCEDAAWBPEEBLYWUPASUPJWJYAACCBSWPVBVRWSEBPYWSAQSVVWWAWCWAVJABCYAJJWELWSYJVAVYYABADPUYCCWPALJWVAJYAPAACWCYCAVAAJPCSAEESPZAAQVYWASEEVWADAEEJWBBSLWPCCCRARVPPSBCBECBWBLAYDJYAWZZYAADJRVSWAAAEAAJWCJCSJVCCAAAAADAADBAAAEAWDWEAYAAAADAAADAADAWDAACYAVADACADADAAADDAAADDPVAABDADAVAAAAPAAVADAEAVDWBRPSSAADA..
   1. AAAAAAPCYAQZAADAADAAAAAAJWYPYADADDAAAAAAAAADDJVAAAADAADDAAAAADAAADAJWYDAAAAAAAADAAADDDJVAAAAPYADAADAAAAAAAADDAJWYAJVAAAAADAAAAAAADAJVDAAAAAAADAAAAAAAAAADAAAAAAADAQSVAAPCCCSVAAAADAAADADDAADDJVDAAAADAAAPYAADAAAAAAAPYJVADDAAAAAAAAAADDQSVADAAAAAADAADAAAAAJBVAJVADADDADAAAAAAAADJWZAAAAAAADAAAAAAAADAPYJWCYADAAAAAAAAADAAADJVADAAAAAAAAADDADADAAAPCSWYAAAAAAAAAADAAADJVAAAADJVAAAAADAAAJVJWCYAAAAAAAAADAAD : 2OT8 : B :  466 :  860 : 0.451
   2. AAAAAAPCYAQZAAAADDDAAAAAJWYJVAAADDDDDAAADAADDJVAAAAAAADDAAADDAAAADAJWZAAAAAAAAAAAAADDDJVAAAAPYADAADAAADDAAAAAAJWYJBVAAAAADAAAADAADDJWZAAAAAAADADAAAAAAAAAAAAAAAAAADJVDAPFRWSVAAAAAAAAADDAAADDJVAAAAAAAAAPYADDAAAAAAAJVJVAAAAAAADAAAAADDQSVADAAAAAADAAAAAAAAJBVAJVDDAADADAAAAAAADDJWZAAAAAAADAAAAAADAAAJVJWCYAAAAAAAAAAAAAADDJVADAAAAAADAADAADADAAAPCSWYAAAADAAAAADAAADJVAAAADAAAAAAADAAAJVJWCYAAAADAAAADAAA : 2QMR : A :  466 :  860 : 0.446
   3. VADDAAPSVAQZAADAADDAAAAAJBVJVAADDDAAAAAAAAADDJVAAAAAAAADDAADAAAADDAJWYAAAAAAAADAAAADADJWYAAAJVAAAADAAADAADAAAAJWYAJVAAAAADADAADAAAAJWZAAAAAAADAAAAAAAAAAAAAAAAAAAADJVAAPFRWSVAAAAADAADDDAAAADJVDAAAAAAAAJVAAAAAAAAAAPYJVAAADDAAAAADDAADQSVADAAAAAAAAAAAAAAAJBVAJVDAAADDAAAAAAAADDJWZAAAAAAADAAAAAAAAAAPYJWSVAAADDAAAAAAAAADDJVADAAAAAADAAAADDAAAAAPCYPYAAAAAAADAADAAADJVAAAADAAAAAAAAAAAJVJWCYDAAAADADDAAAD : 5YVG : A :  466 :  860 : 0.443
   4. AAAAAAPCYADDAADDADAAAAAAAAPCZAAADDDDDAAAAAADDJVAAAAAAAADAAAADAAAADAJWYDAAAAAAAAAAAADDDJVAAAAPYADAADAAAADAAAADAJWYAJVAAADAAAAAADAADAJVDAAAAAAADAAAAAAADDAAADAAAAAADDJVDAPCCCSVAADAAAAAAAAAAADDJVAAAAADAAAPYAADAAAAAAAPYJVADAAAAAAAAADAADDJVADAAAAAAAAAAAAAAAAJVAJVADAAAAAAAAAAAAADJWZAAAAAAADAAAAAAAAAAPYJWCYAAAAAADAAAADAADDJVADAAAAAAAAADDAAADAAAPCSWYDAAAAAAAAAAAAADJVAAAADJVAAAAAAAAAJVJWCYAAAAAAAADDAAA : 2H4M : A :  466 :  860 : 0.442
   5. AAAAAAPCYAQZADDAADAAAAAAJWYJVAAAADDADAAAAAAADJVAAAADAAAAAAADAAAAAAAJWYDAAAAAAADDAAAAADJWYAAAPYAAAADAAAAAAAAAAAJWYAJVAAAAAAADAADAADDJVDAAAAAAADAAAAAAADDAAAAAAAAAAADJVDAJWCCSVAADAAAAAAAAADDDDJVAAAAAAAAAPYAADAAAAAAAJVJVADAAAAAAAAAAAADQSVADAAAAAADAAADAAAAAJVAJVAAAAAADAAAAAAAAAJWZAAAAAAADAAAAAAAADAPYAPCYADADAAAADDDDAAADJVADAAAAAADAADAADADAAAPCSWZAAAADAAAAAAAAAQSVAAAADJVAAAAADAAAJVJWCYAAAAADAADDAAA : 5J3V : A :  466 :  860 : 0.442
   6. JWCYAAAADDDADDDDAADDAADAAAAAAAAAAAAAAADDADAAAAAAAAAAAAAAAAAAAAAAAAAJBBBBVADAAAADAAAAAADAAAAAAAAAAAAAAAAAAAAAAAAAAAAAAAAAPSVJBBWSBBVAJVAJBWCCCYAAADDAADDAAAAADAAAAAAAAAJBBBWYAAAAAAADADAAAADAAAAADAAPCYAAAAAAAAAAAAAAAAAAAAAAAAAAAAAAJWYAJWSBBEVJVAJBBBW$CSVAAAAJBWCCCCYADAAAAAAAAAAAAAAAAAAAAAAAAAAAAAAAAAAAAAAAAAAPSWYAADAAAAAAADAAAAAAAAAAAAADAAAAAAAAADDAAAAAAAAAAADAADADDAAAAAAADAAAAAAAADDAAAAAAAAADAA : 8AT3 : D :   50 :  444 : 0.442
   7. AAAAAAPCYAQZAAAADDDAAAAAJWYJVAAADDDDDAAADAADDJVAAAAAAADDAAADDAAAADAJWZAAAAAAAAAAAAADDDJVAAAAPYADAADAAAAAAAAAAAJWYAJVAAAAADDDAAAAADAJWZAAAAAAADADAAAAAAAAAAAAAAAAAADJVDAPFRWSVAADAAAAAADDAAADDJVAAAAAAAAAPYADDAAAAAAAJVJVAAAAAAADAAAAADDQSVADAAAAAADAAAAAAAAJBVAJVDDAADADAAAAAAADDJWZAAAAAAADAAAAAADAAAJVJWCYAAAAAAAAAAAAAADDJVADAAAAAADAADDADADAAAPCSWYAAAADAAAAADAAADJVAAAADAAAAAAADAAAJVJWCYAAAADAAAADAAA : 2QMR : D :  466 :  860 : 0.441
   8. AAJVJVADAAAAAAAAAAAAAQSWSVAAAAAAAAAAAAJVJVAAAAAAAAAAADAADDJVAAAAAAAADAAAAAAPYJVAAAAAAADAAAAJBBVAAADDAAAAAAAAAAPYJVAAAAAAAAAAAAAAAPYPSBVAAAAAAAAAAAAAAJVJVAAAAAAAAAAAAAAAJBWZAAJWSBVDDAAAAAADAAAAPCZAAAADAAAAAAAJEBVJVDAAAAAAAAAAAAJWYJWZADDAAAAAAADAAAAPCCYAPYAAAAAAAAAAAAAAAAAAPCYAAAAAAAAAAADAAADJVAAAAAAAAAAPSWYAPCYAAAAAAAAAAAAAJWYAAAADAAAAAAAAAAPYJVAAADAAAAAAAAAAAAJVAAAAAAAAAAAAAAAJBBVAAAAAAAAAAAA : 7RW8 : B :   22 :  416 : 0.441
   9. AAAAAAPCYAQZAAAAADDAAAAAJWYJVAAADDAADAAAAAAAAJVAAAAAAADDAAADAAAAADAJWZAAAAAAAAAAAAADADJVAAAAPYAAAADAAADAAAAADAJWYAJVAAADADAAAADDAADJVDAAAAAAADADAAAAAADAAAAAAAAAAAQSVADJWCCSVAAAAAADAAAAAADDDJWYAAAADAAAPYAADAAAAAAJWYJVAAAAAAAAAAAAAADQSVADAAAAAADAAAAAADAJBVAJVDAAADAAAAAAAAAADJWYAAAAAAADAAAAAAAAAAJVJWCYAAADAAAAAAAAAADDJVADAAAAAADAAAAAAADAAAPCSWYAAAAAAAAAAAAAADJVAAAADJVAAAAAAAAAJVJWCYAAAAAAAADDAAA : 7VPW : A :  466 :  860 : 0.441
  10. AAAAAAPCYAQZADAAADDAAAAAJWYJVAAADDAADAAAAAAAAJVAAAAAAAAAAAADAAAAADAJWYAAAAAAAAAAAAAAADJVAAAAPYAAAADAAADDAAAAAAPCYAJVAAADADADAADAADDJVDAAAAAAADAAAAAAADAAAADAAADAAADJVDAJWCCSVAAADAAAAAAAAAADDJVAAAADDAAAJVAAAAAAAAAAJVJVAAADAAAAAAADAADQSVAAAAAAADDAAAAAAAAJBVAJVAAAAAAAAAAAAAADDJWZAAAAAAADAAAAAAAAAAPYJWCYAAAAAAAAAAAAAADDJVADAAAJVAPYAADADADAAAPCYPYAAADAAAAADDAAADJVAAAADJVAAAAAAAAAJVJWCYAAAAAAADDDAAA : 4FQ3 : A :  466 :  860 : 0.439	
	Mix12-3	..BPCWACPCCWPCVADAEEZABVBVSYWPYVJBPADAWJAEBVAPAAJAAAJEAADAACJAJBEAVPCPCADDAAEJYAJUJJOADARJEDAYBCYAYLCJAALSAABAWJDEEBLYBUPABUPJVASAAVWBSCLAWVRPEEBCVWJAQBJVWABEEWAVJABBEASJWBLWSAAASVYYABAJCUVCCACALCBSEJYSCAAAEPCCAABAJWSWBEEVYZAWYVVWAJJEVBWABEEAWBBSLBCCCWRARESVCBCAWCBPBLDYDCYAWZWADADJSVSWAASEAVYWCCCSAVCVDAAAADAADDDAAAEADWEPYAAECAQAAAWADAAAAACZDVAAAAJDAAPAAAAAAADDCVAJWBSDAAPAASEWWVAADEAAYEYRPSYAAAE..
   1. AAAADAAPSVAJVADAAAAAAAAAAAAAAAAAAAAJWZAJVAAAAADDAAAADAAAAADDJBBVAAAADAAAAAAAAAPYJVAAAAAADAAAJBVAAAAAAAAADDAAAAAPZAPSEWCYAAAAAAAADDAAPCYAAAAAAAAJVJVAQSVAAAAAAAADAAAAJVAJBVJVAAAAAAADAAAAJVAPCYAAAAAAAAAAADAAAAAAAJVJEBVAPYAAAAAAAAAAP$SVAAAAAAADDAQSVPYAAAAAAAAAAAAADAAAAADADDAPSVAAAADJBWYAAAPYAAAPCZAAAAAJVAAAADAADJBVAAAAAAAADAPYJVAAAAAAAAAAAAADDAJVJWCYAAAAAAAAADAAPYJVAAAAAAAAJBWZJBVAADAAAQSBVJVADAA : 5ZCS : E :  501 :  895 : 0.433
   2. AAAADAAPSVAJVADAAAAAAAAAAAAAAAAAAAAJWZAJVAAAAADDAAAADAAAAADDJBBVAAAADAAAAAAAAAPYJVAAAAAADAAAJBVAAAAAAAAADDAAAAAPZAPSEWCYAAAAAAAADDAAPCYAAAAAAAAJVJVAQSVAAAAAAAADAAAAJVAJBVJVAAAAAAADAAAAJVAPCYAAAAAAAAAAADAAAAAAAJVJEBVAPYAAAAAAAAAAP$SVAAAAAAADDAQSVPYAAAAAAAAAAAAADAAAAADADDAPSVAAAADJBWYAAAPYAAAPCZAAAAAJVAAAADAADJBVAAAAAAAADAPYJVAAAAAAAAAAAAADDAJVJWCYAAAAAAAAADAAPYJVAAAAAAAAJBWZJBVAADAAAQSBVJVADAA : 5ZCS : F :  501 :  895 : 0.433
   3. DAADADQSWSVAAAAAAAAAAAJWYJVAAAADAAAAAADAAAAJVAAAAAAAAAADAAAAJVJVAADAAAADAAAAJVJVAAAAAAAAAAAAAAAPYJVAAADAAAAAAAAAAADJVAADAAPSVADAAAAAAAPYJVADADAAADAADDDAAAPCSVJWSBWCYAAAAAAAAAAAAPCZAAAADAAAAAAAJEBVPYAADAAAAADAAAAAAJVJVAAAAAAAADAADAAAAPSVAPYAADAAAAAAAAADAAAAAPCYAAAADAAAAAAAAAADJVAAAAAJVAAAPSWYAPCYAAAAAAAAAAADAJWYAAAAADDADAAAAAAPYJVAAAAAAAAAAAAADAAJVAAAAAAAAAAAAAAAJBWYAAAADAAAAAAAAADDJVAAAAAAAAA : 6D83 : B :   37 :  431 : 0.429
   4. AAAAAQSWSVAAAAAAAAAAAJBVJVAAAAAAAAAADDAAADJVAAAAAAAAADAAAAAPYJVAADDAAAAAAAAJVJVAAAAAAAAAAAAAAAJVJVAAADAAAAAAAAAAADJVAADADJBVADAAAAAAAPYJVAAADAAADAAADAAAAPCSVJBVJWCYAAAADADDAAAAPCYAAAAAAAAAAAAJEBVJVAADAADAADAAAAAAJVJVAAAAAAAADADDAAAAPSVAPYAAAAAAAAAAAADAAAAAPCYAAAADAAADAADAADDJVAAAAAAAAAAPSWYAPCYAAAAAAAAAAAAAJWYAAAAADDAAAAAAAAPYJVAAAAAADAAADAADAAJVAAAAAAAAAAAAAAAJBBVAAADAAAAAAAAAAADJVAAAAAAAADA : 6DFF : B :   38 :  432 : 0.423
   5. AAAAAAAPSBBBVAJBBBWSBBBVJVJWCYJWCZAAAAAAAAAAAAAAADAAAADAAAPSWSEWSEWYAAADAADAAAAAAAADAAAAAAAAAAAAAAAAAAAAAAAAAADAAAAAAAAAAAAAAAAAAAAAAAAAAAAAAAAAAAAAAAAAAAAAAADDDAAAAADDAADAAAAAADAAAAAAAAAAAAAAPCYADDADAAAJVJLCYDDADDAAAAAAAPYJBBVJVAAADDDAAJVAAADAAAAJWYAAJWYAADDAADAAADDAADAAJVDAAAAQSVAAAAAAAADAAAAAAADAAAAAADDDAAAADDADDAAAAAAAAAAADDDAAAAAAAAAAAAAAAADAAAAAADAAAAAAAAAAAADAAAAAAAAAADAAADAAAAAAAADAAA : 5NVU : A :  3106 :  3500 : 0.423
   6. AAAAAAAPSBBBVAJBBBWSBBBVJVJWCYJWCZAAAAAAAAAAAAAAADAAAADAAAPSWSEWSEWYAAADAADAAAAAAAADAAAAAAAAAAAAAAAAAAAAAAAAAADAAAAAAAAAAAAAAAAAAAAAAAAAAAAAAAAAAAAAAAAAAAAAAADDDAAAAADDAADAAAAAADAAAAAAAAAAAAAAPCYADDADAAAJVJLCYDDADDAAAAAAAPYJBBVJVAAADDDAAJVAAADAAAAJWYAAJWYAADDAADAAADDAADAAJVDAAAAQSVAAAAAAAADAAAAAAADAAAAAADDDAAAADDADDAAAAAAAAAAADDDAAAAAAAAAAAAAAAADAAAAAADAAAAAAAAAAAADAAAAAAAAAADAAADAAAAAAAADAAA : 5NVU : B :  3106 :  3500 : 0.423
   7. AAAAAAQSWSVAAAAAAAAAAAJWYJVAAAADAAADAADAAADJVAAAAAAAAADAAAAAJVJVAAAAAAAAAAAAJBBVAAAAAAAADAAAAAAPYJVAAAADAAAAAAAAAADJVAADADJBVADAAAAAAAPYJVAAAADAADAAAADAAAPCSVJBVJLCYAAAADAADAAAAPCZAAAAAAAAAAAAJEBVPYAADADDAADAAAAAAJVJVAAAAAAAADADDAAAAPSVAPYAADAAAAAAAAADAAAAAPCYAAAADAAADAAAAADDJVAAAAAAAAAAPSWYAPCYAAAAAAAAAAAAAJWYAAAAADDAAAAAAAAJVJVAAAAAADAAADAAAAAJVAAAADAAAAAAAAAAJBBVAAAADAAAAAAAAADDJVAAAAAAAAD : 6CM9 : B :   37 :  431 : 0.422
   8. AADAAAPSVAAAADDAAADDAAAAAJVAJVJVAAAAAADAAAJVAAAAAAAADADAAAAAAJBVAJBWCYDAAAAAAAAAAAAAAAAAAAAAAAAAQSVAJVAAAAAAAAAAAAAAAJVAPSVAAAAAJVAAAAAAAAAAJVAPCYAAAAAAAAAAAADDAAJVJVADJVAAAADAAAAAAAAAAAAAAAAAPSBVAAAAAAAADDAAADADAAAAADDJVAP$ZJBVAAAAAAAAAAAJVJVAAAAAAAAAAAAAADDAAAJVJVAAAAAAAAAAADDAAAAAAAAJWCYJBWCCCSVAPZAAAAAAAAAAAAADAAADPCYADAAAAAAAAAAAPSWCYAAAAAAAAAAADAAAAPSVAJVADAAAAAAAAAAJWYAPCYAAAAAAAAAAAAA : 6GX9 : A :   94 :  488 : 0.422
   9. AAAAAAPCYAQZADDAADAAAAAAJWYJVAAAADDADAAAAAAADJVAAAADAAAAAAADAAAAAAAJWYDAAAAAAADDAAAAADJWYAAAPYAAAADAAAAAAAAAAAJWYAJVAAAAAAADAADAADDJVDAAAAAAADAAAAAAADDAAAAAAAAAAADJVDAJWCCSVAADAAAAAAAAADDDDJVAAAAAAAAAPYAADAAAAAAAJVJVADAAAAAAAAAAAADQSVADAAAAAADAAADAAAAAJVAJVAAAAAADAAAAAAAAAJWZAAAAAAADAAAAAAAADAPYAPCYADADAAAADDDDAAADJVADAAAAAADAADAADADAAAPCSWZAAAADAAAAAAAAAQSVAAAADJVAAAAADAAAJVJWCYAAAAADAADDAAA : 5J3V : A :  466 :  860 : 0.422
  10. AAADDDADDDDAADDAADAAAAAAAAAAAAAAADDADAAAAAAAAAAAAAAAAAAAAAAAAAJBBBBVADAAAADAAAAAADAAAAAAAAAAAAAAAAAAAAAAAAAAAAAAAAAPSVJBBWSBBVAJVAJBWCCCYAAADDAADDAAAAADAAAAAAAAAJBBBWYAAAAAAADADAAAADAAAAADAAPCYAAAAAAAAAAAAAAAAAAAAAAAAAAAAAAJWYAJWSBBEVJVAJBBBW$CSVAAAAJBWCCCCYADAAAAAAAAAAAAAAAAAAAAAAAAAAAAAAAAAAAAAAAAAAPSWYAADAAAAAAADAAAAAAAAAAAAADAAAAAAAAADDAAAAAAAAAAADAADADDAAAAAAADAAAAAAAADDAAAAAAAAADAAAAAAA : 8AT3 : D :   55 :  449 : 0.420	
	Mix12-4	..BPCWAPCPCABPVYADAWZDABBBCBAVYYAPPDAEAAAEABAAWJSWADAJAVADCYJVAWECY$YWCSAAAPACAFJUPAOAADDPEAPABEVJCPCEBALCEDAJWBPBEBLYWUPASUPJWJYJACCCSWPVBARWSPBPYWSAQSVAWWAWCCAVJJBCYAJWWELPSYJVAJYYAEADPUYWCWPCLJWVAWYAPWACWCYSAVAAJPCSAEESPEAAQVYWASEWVWADAAEJWBBSLWPPCCRERVPPSSCBEVBWBLAYDJYAWZZYAADJRSSWAAAAAAJACJCSJCCCAWAAADADDBAAAEAWDAEAYEAAADADADASDAWDABCYAYADACAAADAWADDAAEDDPBAABDAWAVAYAAPAAAADAAAVDWBBPSSBADA..
   1. VPSWCYAAAAAAAAAAAAAAAQYPRBVAAAAAAAAAAAJVJVAAAAAAAAAAAAJEBVJVAAAAAAAAAAAAAAAJVJVAAAAAAAAAADDJBWYAAADDAADAAAAAAAJVAPCYAAADAAAAAAADAADDJVAAJWSWYAAAAAAAAAAJVJVAAAADAADDAADAADDJVADAAAAAAAAAAAAAADDAPCZAAJVAAJW$YPCSVAAAAAAAAAAAAPCCSVJVAAAAAAAAAAAAAAAAAAPCSVJVJVAAAADAAADAAAAAAAAADJVAJVAAAAAAAAAAAAAAAJVJVAAADAAAAAAAAAAAJVAAAAAAAAAAAAAAAAADAJVJVAAAAAAAAAAAAAJWYDAAAAAAAAAAAAADAPCYAAAAAAAAAAAAAADDJBVJVAA : 7RW8 : A :   47 :  441 : 0.435
   2. AAAADDAADAAAAAAAAADAAADPSBVAAAAAAAAAAAAAAAAAAADAAAAAAAAAAAPYAJEWCCSWYPSVAAAAAAAAAAADAAAAAAJVJBWYAAAADAAAAAADAAAAAAAAADAAAAAJVJVAAAAAADAAAADDDAAAADAAAAAAAAPZJBWYAAAAAAAAAAAAAAADAAAAAAAAAPYAAPCCCCSWCZAAAAAAAAAAAAAAAAAAAAAAAAAAAAAAADJVAJVAAADAAAAAAAAADAAADAADAAAAAAPYAAAAAAAAAAAAAAAAAAAAAAAAAAAQYAJWCSVAPCYAAAAAAAAAAAAAAAAAADAAAAAAQYJVAAAAAAAAAAAAAAADADDAADDAAAAJVJVAAAAAAAAAAAAAAAAAAAAADAAADJWSBVA : 5IC1 : A :  1393 :  1787 : 0.433
   3. AAAAAPYPYAAAAAAADPSBBVAAJVAAAAAAAAAAAAJVAAAAAAAAAAAADAAAAAJVPSVAAADAADAAAAAADAAAAAAAADAJW$ZAAAAAPCCYAAADDDPSWCYDAAAADAAAAAAAAAAPSVAAAAAAAAJBWSBVAPSVAAPSVADADAAAAJWYAAAJEBVAAAAAAADADAAAAAPYPCYAAADAAAAAAAAAADAAAAAAADAAAAAAAAADAAAAAAAJBBVAAAAAAAADDAAAAAAPCYAAAAAAAAAADDAPCYDAAAAAAAADAAPYAADAAAAAJWZAAAAAAAAAAAAADAAAAADDDDPRWCZDAAAADAAAAAAAAAAAADAAAPCCYADAAAAAAAAADAAAAAAAJVAAAAAAAAAAAAAAAPSEVPSVAAD : 7V2W : G :  790 :  1184 : 0.430
   4. AAAAADAADAAAAAAAADDAAADPSBVAAAAAAAAAAAAAAAAAAADAAAAAAAAAAAPYAJLRLFRWYPSVAAAAAAAAAAADAADAAAPYJWCZAAAAAAAAADAADAAAAAAAAAAAAAAJVJVAAAAAADAAAAADAAAAAAAAAAJBVAJVADAAAAAAAAAAAAADAAAAAAAAAAAAAJVAAJBBWCSBWZAAAAAAAADAAAAAAAADAAAAAAADAAAAADJVAJVAAADAAAAAADAADAAADAAAAAAAAJWZAAAAAAAAAAAAAAAAAAAAAAAAAAAQYAJWCSVAPCYAAAAAAAAAAAAAAAAAADAAAAAAQYJVAAAAAAAAAAAAAAADADAAADDAAAAJVJVAADAAAAAAAAAAAAAAAAAADAAADJWSWYJ : 5IC0 : A :  1393 :  1787 : 0.429
   5. AAAAAAPCYAQZAADAADAAAAAAJWYPYADADDAAAAAAAAADDJVAAAADAADDAAAAADAAADAJWYDAAAAAAAADAAADDDJVAAAAPYADAADAAAAAAAADDAJWYAJVAAAAADAAAAAAADAJVDAAAAAAADAAAAAAAAAADAAAAAAADAQSVAAPCCCSVAAAADAAADADDAADDJVDAAAADAAAPYAADAAAAAAAPYJVADDAAAAAAAAAADDQSVADAAAAAADAADAAAAAJBVAJVADADDADAAAAAAAADJWZAAAAAAADAAAAAAAADAPYJWCYADAAAAAAAAADAAADJVADAAAAAAAAADDADADAAAPCSWYAAAAAAAAAADAAADJVAAAADJVAAAAADAAAJVJWCYAAAAAAAAADAAD : 2OT8 : B :  466 :  860 : 0.429
   6. VJEWCYAAAAAAAAAAAAAAAQSWSBVAAAAAADAAAAJVJVAAAAAAAAAAAAJEBVJVAAAADAAAAAAAAAAJVJVADAAAAAAAAADJBWYAAAADAADAAAAADAJVAPCYAAADAAAAAAAAAADDJVAAJWSWYAAADAAAAAJBVJVAAAADAAAAAADAAADJVAAAAAAAAAAAAAAAADAAPCZAAJVDAJW$YJWSVAAAAAAAAAAAAPCCSVJVAAAAAAAAADAADAAAAAPCSVJVJVAAAADAAADAAAAAAAAADJVAJVAAAAAAAAAAAAAAJBVJVAAADAAAAAAAAAAAJVAAAAAAAAAAAAAAAAAAAJVJVAAAAAAAAAAADAJWYDAAAAAAAAAAAAADAPCYAAAADAAAAAAAAADDJBVJVAA : 7Z5C : A :   47 :  441 : 0.429
   7. AADDAADAAAAAAAAAAAAAAAAADAAAAAAAAAAAAAJVAAAAAJVPCYAADAAAAAAAAAADAAAAADAAADAAAAAAADAAAAAAADAAJVJWZAJWYAAAAAAADDAAAAAAAPSVAAADAAAADAADAAAAAAAAADDAADAJVJVAAAADAAAAADAAAAAAAAAADDAAAAAAAAAAAAAAAAPSBBVJWCYAAAAAADADDAADAAAADAAAAAAADAADAAAAAAAAAAAAAAAAAAAAAAAAAAAAAAAJBVAAAAADDDAAAAAAAADADDAAAAAAAADAAAAAJWSVJVAAAAAAAAAAAAADAAAAAAAAAAAAAAAAAAADAAAAAADAAAAAAAAAJBWYAAAAAADAADAAAAAAAAAAAAAAAAAAAAAAAAAAAAA : 6D05 : E :  218 :  612 : 0.428
   8. AADDAADAAAAAAAAAAAAAAAAADAAAAAAAAAAAAAJVAAAAAJVPCYAADAAAAAAAAAADAAAAADAAADAAAAAAADAAAAAAADAAJVJWZAJWYAAAAAAADDAAAAAAAPSVAAADAAAADAADAAAAAAAAADDAADAJVJVAAAADAAAAADAAAAAAAAAADDAAAAAAAAAAAAAAAAPSBBVJWCYAAAAAADADDAADAAAADAAAAAAADAADAAAAAAAAAAAAAAAAAAAAAAAAAAAAAAAJBVAAAAADDDAAAAAAAADADDAAAAAAAADAAAAAJWSVJVAAAAAAAAAAAAADAAAAAAAAAAAAAAAAAAADAAAAAADAAAAAAAAAJBWYAAAAAADAADAAAAAAAAAAAAAAAAAAAAAAAAAAAAA : 6D05 : F :  218 :  612 : 0.428
   9. AAAAAAAAAPYJVAAAAAAAAAAAADJVAJVAAAAAAAAAJVAAAJVAAAAAAAADAAADJVAJVAADAAAAAAJVJVAAAAAAAAADAAAADDJBBVAAAADAAAAADDJVADAAAAAAAAAAAAAAAJBVDDJVAAADAAAAAAAAAAAAQSBVAAAAADAAAAADQSVAJVAAAAAAAAAAAAQYJVAAAAAAAAAAAAAJVAJVAAAAAAAAAAAAQYJVAAAAAAAAAAPSVAAPYPYAAAAAAQSBVAAAADAAAAAAAAAAAAAAAAAAAAAJWSWZJBVADAAAAAAAAAAAAAAAAAAAAAAAAAAAADAAAAAADAAAAAAADAADDPSBVAAAAAAAAAADAAAAJVAAAAAAAAAAAAAAAAADAADDAAAAPYJVADAAJWY : 6HD5 : t :  245 :  639 : 0.428
  10. ADAAAADADDDJVAJVADAAAADAAAAAAADAAAAAADAAAAAAAAJVJVAAAADDDAAADDDDAAAAAAADAADAAAAAPCSVAAAAAAAAAAAAAAAAADADDAAAJVJBEWCYAJBVDAAADAAADAAAAAAAAADAAAAAADJVJBVAAAAAAAAAAAAAADAADADAAAAAAJVJVAAAAAAAADAAAAADDAAAAADAADDAAAJVJBVAAAAAAADAADAADDDAAAADAAJVAJWSVDDDDADDAAAAAAAAQSBWSVJBWCYAAAADAAADAAAAADADAAAADAAAAPCYAAAAAAAADDAAAADDAADAAAAAAAAJVJVADAAADADDAAAAAAAAADDDAAAAAADDJVAJVAAAAADADAAAAAAAAAAAAAADAPSVAJV : 6R9T : A :  965 :  1359 : 0.428	
	Mix12-5	..BPCWACPCPWPCWADAEEYABVEVSYWPSVJBAADAWJDEBVJPAAJADAJEEADAACAAJBSAVPCPSADDDAEJYAPUJJOADARJVDAYYCYAYLEJAALSAABABJDEEBLYBUPABUPJVASAJVWBCCLAWVEPEEWCVWJASBJVAABEEWBVJAABEASJBBLWCAAASVAYABWJCUVCVACAACBSEJZSCABAEPCCYABAJWSWBEBVYZVWYVVWCJJEPBWABEEAWBBSLBCCSWRARESVCBSAWCEPBLDYDCYAWZWADABJSVSWAASEDVYWVCCSAVAVDAZAADAADDDAQAEADWRPYADECAQADAWAPAAAAAAZDVDAAAJDJAPAJAAAAAADCVJJWBSDJAPABSEWWVDADEDAYEYRDSYADAE..	
	Mix13-1	..BPCWACCWCWBSVAJAAEZAAVBVWYAWYVAWPAWAADAEAWAPYASYAAAAAAWACZJAAEEAJPYJCAAWAAAJAJJUPWOAVADJEDPVBCCACYCJBALSBAADWJPEEBLYWUPASUPJYAYWAVCVSCWABYRPSABCYWSAQBVDWAEECPAVJDBBWAJSWBLWSAPAAVYYARAJJUYYCAPSLCASACYSPBAACPYBAAAAJWBWAEEVPAAWBVYWAJEBVBBAAJEAWBBSLBPSCWRAREWVSCCAEEBPBLAYDCYAWZCAAADJRWSWAAAVAVJDCCCSJWCVADAAADASDDAAAABADWEPYDAEWAAYAAAPDAWAAVCZAAAAAAAEAAADAABAADDDPWAJJBAAAAABASBWAJAAAVAAAEBSPSSWAAW..	
	Mix13-2	..BPCWAWCCCCBCVDAAAEZPAVBSCCAPYCABPVADAJAEAVACWVSAAJAEACAWCCJEABESYPYPCDADADAAAAJUPJOAAJDJESPYBJVDCLCABALJEJAAWEPEEBLYWUPASUPJWAYAADCBSYPDBVRBSEBBYWSAQJVVWCADCWAVJABWYAJJWALWSVJBAVYYABAAPUYCCCPALSWAAJYPPAARWAYCAAAAJCCAAEECPZAWQVYWAAEEVVAAAEEPWBBSLEPCCARARUPWSBCWECBBBLAYDCYAWZZBAADJRVSWAAAEAPJWCACSJVCWAAAAADAADAAAABADDWEEYAAJAVAAAPAADAWAAACAAVASAAADAPAAAWDAAADDPVABBVADASAAAPPSAVAVAEADDABRPSSAAAA..	
	Mix13-3	..BPCWACWCCWYCVAJAJEZAWVBVWYVPYVDBPAWABJAEAVAPYAAAAAVEAAWAVCJABBEAJPSPCAJDAAAJAAJUPJOAVASJEDVYBCCASLCJPALSBAEAWJAEEBLYWUPAJUPJYACAAVPBSCWABVRPBEBCYWAAQBYVWAEEWWAVJABBWAPJWBLWSAPADVYYABAJJUYCCASALCASVJYSAAAACPWCAAVAJWBWAEEV$ZAWBVCWAJAEVBBAEEEAWBBSLBWCCWRAREWVRBCABCBPBLJYDCYAWZCAVADJRVSWAADEAVJWCCCSJVCVSAAAADAADDJAAABADWEPYAAEWAWAAADADAWAAACZAVAAAAEDAAJAAABAAADDYVAJJBYDAAYAASBWJVAABEAAAEPRPSAAAAW..	
	Mix13-4	..BPCWAWCWCCBSVDAAAEZPAVBSCCAWYCAWPVADADAEAWACWVSYAJAAACAWCZJEAEESYPYJCDAWADAAAJJUPWOAAJDJESPVBJVDCYCABALJEJADWEPEEBLYWUPASUPJWAYWADCVSYPDBYRBSABBYWSAQJVDWCADCPAVJDBWYAJSWALWSVJBAVYYARAAPUYYCCPSLSWAACYPPBARWAYBAAAAJCCAAEECPAAWQVYWAAEBVVAAAJEPWBBSLEPSCARARUPWSCCWEEBBBLAYDCYAWZZBAADJRWSWAAAVAPJDCACSJWCWADAAADASDAAAABADDWEEYDAJAVAYAPAPDAWAAVCAAAASAAAEAPADAWDAADDDPWABBVAAASABAPPSAJAVAVADDABSPSSWAAA..	
	Mix13-5	..BPCWACWCWWYCCAJAJEVAWVSVWYVPPVDBWAWABJBEAVAPYAAACAVEVAWAVCSABBWAJPSPJAJDSAAJAAJUPJOAVASJWDVYACCASLYJPALSBAEAJJAEEBLYWUPAJUPJYACABVPBSCWABVBPBEBCYWAAQBYVCAEEWWRVJAWBWAPJLBLWVAPADVZYABAJJUYCYASAECASVJVSAAWACPWCAAVAJWBWAEEV$ZBWBVCWYJAEWBBAEEEAWBBSLBWCYWRAREWVRBWABCWPBLJYDCYAWZCAVAAJRVSWAADEYVJWSCCSJVPVSAEAADAADDJAVABADWEPYAJEWAWAWADAAAWAAAVZAVJAAAEDBAJADABAAARDYVVJJBYDZAYAJSBWJVYABEJAAEPRCSAACAW..	
	Mix14-1	..BPCWACCACWBJVAVAAEZAAVBVZYAAYVAAPAJAAWAEAVAPDASPAAACAAJACAJAAPEASPYACAABAAAJALJUPJOAJADJEDPZBCEACFCJBALSEAAYWJPEEBLYWUPASUPJWAYPAVCSSCEABSRPSWBCYWSAQBVVWADECYAVJPBBZAJJWBLWSAYAAVYYABAJPUYJCAPBLCESAJYSPDAALPYWAAAAJWEWAEEVPBAWEVYWAJEAVBCAAEEAWBBSLBPJCWRARECVSWCAEJBPBLAYDCYAWZYAAADJRCSWAAAEAVJVCCCSJACVAAAAADAADDAAAARADWEPYCAEDAASAAACDAWAAPCZABAAAAALAAAVAADAAADDPVAJCBAVAAAJASWWABAAADAAJEBAPSSAAAR..	
	Mix14-2	..BPCWAACCCPBCVJAAAEZVAVBCCAAPYVABPBAVAJAEAVADWSSAASAEAPAECCJJABEWYPYPCAADAJAPAAJUPJOAAADJEZPYBPVPCLCJBALREPAAWSPEEBLYWUPASUPJWAYAASCBSSPJBVRASEBEYWSAQAVVWDALCWAVJABLYAJJWBLWSWJSAVYYABAVPUYCCBPALCWBAJYQPAADWWYCAAAAJACBAEEUPZAWQVYWAREEVAAAAEEYWBBSLBPCCPRARBPVSBCEECBCBLAYDCYAWZZVAADJRVSWAAAEASJWCBCSJVCSAAAAADAADDAAAYACDWEPYAACAJAAAJAADAWAAACCAVAAAAADAVAAABDAAADDPVAWBYADAWAAACPJAVAAAEAJDJBRPSSAAAA..	
	Mix14-3	..BPCWACACCWCCVAVAVEZARVBVZYAPYVABPAJAEJAEAVAPDAYAAAYEAAJAYCJAABEASPJPCAADAAAJAAJUPJOAJAAJEDJYBCEAALCJBALSEAVAWJWEEBLYWUPASUPJWAJAAVCBSCEABVRPJEBCYWSAQBSVWADEDWAVJABBZABJWBLWSAYAWVYYABAJPUYCCAJALCESPJYSBAAALPACAAAAJWEWAEEVBZAWEVYWAJPEVBCAAEEAWBBSLBPCCWRARECVWBCAECBPBLAYDCYAWZYAAADJRVSWAAEEAVJWCCCSJVCVVAAAADAADDSAAARADWEPYAAEDADAAAVADAWAAACZAVAAAARDAASAAADAAADDJVAJCBVDAAAAASWWSVAAJEAAJEVRPSRAAAR..	
	Mix14-4	..BPCWAACACPBJVJAAAEZVAVBCCAAAYVAAPBAVAWAEAVADWSSPASACAPAECAJJAPEWYPYACAABAJAPALJUPJOAAADJEZPZBPVPCFCJBALREPAYWSPEEBLYWUPASUPJWAYPASCSSSPJBSRASWBEYWSAQAVVWDALCYAVJPBLYAJJWBLWSWJSAVYYABAVPUYJCBPBLCWBAJYQPDADWWYWAAAAJACBAEEUPBAWQVYWAREAVAAAAEEYWBBSLBPJCPRARBPVSWCEEJBCBLAYDCYAWZZVAADJRCSWAAAEASJVCBCSJACSAAAAADAADDAAAYACDWEPYCACAJASAJACDAWAAPCCABAAAAALAVAVABDAAADDPVAWBYAVAWAJACPJABAAADAJDJBAPSSAAAA..	
	Mix14-5	..BPCWACCJCWBBVABAAEZAAVBVEYABYVASPASAASAEAVAPJASJAAAPAALACDJAAAEACPYPCAAJAAAJAWJUPJOAPADJEDPBBCBACJCJBALSEAABWJPEEBLYWUPASUPJWAYCAVCBSCBABDRPSBBCYWSAQBVVWAAECZAVJABBSAJJWBLWSACAAVYYABAJPUYSCAPWLCWSAJYSPVAAPPYYAAAAJWCWAEEVPWAWQVYWAJELVBVAAEEAWBBSLBPVCWRAREAVSECAEWBPBLAYDCYAWZVAAADJRVSWAAAEAVJPCCCSJBCVAAAAADAADDAAAAYADWEPYVAEVAAAAAAWDAWAAJCZADAAAAARAAALAADAAADDPVAJLBACAAACASAWAWAAACAADEBVPSSAAAA..	
	Mix15-1	..BPCWACCJCWBBVABAAEZAAVBVEYABYVASPASAASAEAVAPJASJAAAPAALACDJAAAEACPYPCAAJAAAJAWJUPJOAPADJEDPBBCBACJCJBALSEAABWJPEEBLYWUPASUPJWAYCAVCBSCBABDRPSBBCYWSAQBVVWAAECZAVJABBSAJJWBLWSACAAVYYABAJPUYSCAPWLCWSAJYSPVAAPPYYAAAAJWCWAEEVPWAWQVYWAJELVBVAAEEAWBBSLBPVCWRAREAVSECAEWBPBLAYDCYAWZVAAADJRVSWAAAEAVJPCCCSJBCVAAAAADAADDAAAAYADWEPYVAEVAAAAAAWDAWAAJCZADAAAAARAAALAADAAADDPVAJLBACAAACASAWAWAAACAADEBVPSSAAAA..	
	Mix15-2	..BPCWAJCCCJBCVVAAAEZSAVBPCSAPYVABPZAAAJAEAVAQWPSAABAEAAAJCCJCABEVYPYPCAADAAASAAJUPJOAAADJEDPYBBVQCLCJBALZEYAAWPPEEBLYWUPASUPJWAYAAWCBSZPBBVRCSEBCYWSAQCVVWSAACWAVJABBYAJJWBLWSAJEAVYYABAJPUYCCSPALCWCAJYJPAAWWPYCAAAAJBCWAEEEPZAWQVYWABEEVJAAAEEAWBBSLBPCCWRARLPVSBCAECBSBLAYDCYAWZZZAADJRVSWAAAEAWJWCWCSJVCRAAAAADAADDAAAWABDWEPYAAVARAAAAAADAWAAACLAVAAAAADAJAAAJDAAADDPVAEBSADAPAAARPBAVAAAEASDVBRPSSAAAA..	
	Mix15-3	..BPCWACJCCWACVABALEZACVBVEYAPYVABPASAPJAEAVAPJAZAAADEAALAJCJAABEACPYPCAADAAAJAAJUPJOAPABJEDWYBCBAVLCJBALSEAWAWJPEEBLYWUPASUPJWABAAVCBSCBABVRPAEBCYWSAQBCVWAAELWAVJABBSAYJWBLWSACABVYYABAJPUYCCAWALCWSSJYSJAAAPPJCAAAAJWCWAEEVIZAWQVYWAJBEVBVAAEEAWBBSLBPCCWRAREAVBBCAECBPBLAYDCYAWZVAAADJRVSWAAAEAVJWCCCSJVCVRAAAADAADDPAAAYADWEPYAAEVACAAAAADAWAAACZAVAAAADDAAQAAADAAADDLVAJLBDDAAAAASAWDVAAAEAADEWRPSVAAAA..	
	Mix15-4	..BPCWAJCJCJBBVVAAAEZSAVBPCSABYVASPZAAASAEAVAQWPSJABAPAAAJCDJCAAEVYPYPCAAJAAASAWJUPJOAAADJEDPBBBVQCJCJBALZEYABWPPEEBLYWUPASUPJWAYCAWCBSZPBBDRCSBBCYWSAQCVVWSAACZAVJABBYAJJWBLWSAJEAVYYABAJPUYSCSPWLCWCAJYJPVAWWPYYAAAAJBCWAEEEPWAWQVYWABELVJAAAEEAWBBSLBPVCWRARLPVSECAEWBSBLAYDCYAWZZZAADJRVSWAAAEAWJPCWCSJBCRAAAAADAADDAAAWABDWEPYVAVARAAAAAWDAWAAJCLADAAAAARAJALAJDAAADDPVAEBSACAPACARPBAWAAACASDVBVPSSAAAA..	
	Mix15-5	..BPCWACJCJWACYABALECACVBVEYAPBVABJASAPJAEAVAPJAZAVADEBALAJCDAABDACPYPWAADAAAJAAJUPJOAPABJEDWYCCBAVLAJBALSEAWASJPEEBLYWUPASUPJWABAEVCBSCBABVRPAEBCYWSAQBCVJAAELWWVJADBSAYJWBLWWACABVYYABAJPUYCCAWALCWSSJSSJAAAPPJCAAAAJWCWAEEVIZDWQVYWSJBEYBVAAEEAWBBSLBPCVWRAREAVBBVAECBPBLAYDCYAWZVAAADJRVSWAAAEBVJWCCCSJVCVRAAAADAADDPASAYADWEPYAZEVACAPAAASAWAAAJZAVAAAADDEAQABADAAADDLVAJLBDDYAAAESAWDVJAAEBADEWRZSVAAAA..	
