# Supplementary material for: Conformational ensembles for protein structure prediction
Source: Sci Rep. 2025 Mar 12;15:8513. doi: 10.1038/s41598-024-84066-z (PMC11904239; doi:10.1038/s41598-024-84066-z)
Supplement: Supplementary file 4 — Supplementary Information 4. [file 41598_2024_84066_MOESM4_ESM.rtf]

Q8GT36 · Q8GT36_SPIOL
Protein  Thylakoid soluble phosphoprotein
Gene  tsp9


PFVM (Protein Folding Variation Matrix)
     0000000000000000000000000000000000000000000000000000000000000000000000000000000000000000000000000001111 
     0000000001111111111222222222233333333334444444444555555555566666666667777777777888888888899999999990000   
     1234567890123456789012345678901234567890123456789012345678901234567890123456789012345678901234567890123   
     MSSLPFVFGAAASSRVVTAAAAKGTAETKQEKSFVDWLLGKITKEDQFYETDPILRGGDVKSSGSTSGKKGGTTSGKKGTVSIPSKKKNGNGGVFGGLFAKKD
   1 ..APAAQADDAAAEAAAWYAAACYCAAJAABAWYAAAAAAAAAYVCBJWAESBAAWYSCAACYACWAZCAAAASPZCYSCBBPDJBAQCAVYASDAAAAAB..
   2 ..JJCBDEWADWDAWRBPADDDYCJVBAYJYVPABDDDJYDEVZCBCADWWCVDJECJPVBAPBBPJ$JYPWBAJ$JPEAEEBSCVWW$QJPCVJCCRDDW..
   3 ..PCBSEDABCESCEEJDZVCCSVPCDBJDDSBDD  E SJBJBYJJVABBJRPCQJYBDESQSEESADSSPJVQADBPSJAJVVYCDASQSPALWWPVJD..
   4 ..WYDDYBVJVDYJVBEADBJBJAABJVWEWBAJE    DSDWAAPSBBSABWJDABPJEJBUYJA$SBWVJSJASBACDAJEAPASBBJZAQDAVDCJPE..
   5 ..BWREJVJZWPBWDDSVJEEVBWYJVEBBAJJVW    CCSDWJSVYJECAESVBVBSBCPJWYVDCACBYDYVCACBELVCCSJJVQYWVSRBYPDYWJ..
   6 ..CAJJBWBEJVZPB VEBJBJVBBWCWVVPUCZ     E JCJPAWSEVJVJBWCACZZDWDPSJEVWJJVWBBVWWWYWYAJWQBSWVAJBEYDYB BC..
   7 ..VBSR  EWZYCBP WBECPQWJSYSCDZSWEE     P PPSSVPELYS ZCEYSWAYVQWCPBVPSBCBPPYPSVVWDWSYAPPAJPCQWYPLJJ VP..
   8 ..  VY  C PJQVJ  CWPZSA VRYRECVYIL     J QBCWWR CCY  WYVPAYJPEAJRQBQYVUCRWWQYEARP WBECLYSWEWJWEJSV  A..
   9 ..  E   P SZP L  IR$SPE WSEYPWE SS     I REEBY  PLP  V$$EVVPWFSVDYRJPPWSCD JPQYJ  R L$ EVZPCVJCPZS  L..
  10 ..      L BS  S  JVRVWR ZPQ SPQ        F VQP    VZZ   PJW RQ JCZAZUYQ Y E  YQSJB  Y    PEBBZYZQS E  S..
  11 ..      S EB     LSSY   QD  U          W  RV           D$ W  LVEWS BE   L  BEZZL  L    R CSDZBW  Z  U..
  12 ..        YC     YPW     E             R   D           ZL $  UEIFD W    U  W  F        Z EYB CF     V..
  13 ..        FL      QY     Q             Z                D D  VR$LR E    V  E             IDE PS     Y..
  14 ..        LQ      CF                                    F     BLVC I       I             LRL  U      ..
  15 ..        QR                                            I      DZF                        UO         ..
  16 ..        R                                             R      Q L                                   ..
  17 ..        H                                             U      U                                     ..
  18 ..        U                                                    F                                     ..
  19 ..                                                             R                                     ..
DonE!


A set of mutiple conformations in PFSC strings formed from PFVM
PFVM-01:	..APAAQADDAAAEAAAWYAAACYCAAJAABAWYAAAAAAAAAYVCBJWAESBAAWYSCAACYACWAZCAAAASPZCYSCBBPDJBAQCAVYASDAAAAAB..
CouPlinG: 	..APCSEEWDAAAAAAAPYAAAJVJVAJWADAPYAAAAAAAAAAAJBVDAAJBBVQYJBVAAPSBWAAJWAAAAPZJBEEBBBVJBWQCSVPCSLCCRVAD..
2-1 MixinG ..APCAQAWDAWAEWAAWAAADCYJAAJYABVWYBAAAJAAEAYCCBJDAECBAJWYSPAAAYABWAZJAAWASJZCYECBEPDCBAQ$AVPASJAAADAB..
2-2 MixinG ..JPAADADDDADEAABWYADAYYCABJAAYAPYAADAAADAVYVCCJWAWSVAAWCSCABCPACWJZCAPABSPZJYSCEBBDJBWQCAJYCSDACAAAW..
2-3 MixinG ..AJAAQEDDAWAAAAAPYAADCCCAAAAABVWAAAADAAAEAZVCBAWAECBDAWYJCAAAYBCWA$CAAWAAPZCPSCBEPSJBAWCAVPAVDAARAAB..
2-4 MixinG ..JPCADAWDDADEWABWAADAYYJABJYAYAPYBADAJADAVYCCCJDAWSVAJWCSPABCPABWJZJAPABSJZJYECEBBDCBWQ$AJYCSJACADAW..
2-5 MixinG ..AJABQEDAAWAAARAPYDADCCCVAAAJBVWAADADAYAEAZVBBAWWECBDAEYJCVAAYBCPA$CYAWAAP$CPSABEPSJVAWCQVPAVDCARADB..
3-1 MixinG ..APBAQAADAEAEEAAWZAACCYPAAJJABSWYDAAAAAABAYYCBJAAEJBACWYSBAASYAEWAZDAAPASQZCYPCBAPDVBAQAAVSASLAAAVAB..
3-2 MixinG ..PPAAEADDCASEAAJWYACASYCADJAADABYAAAAAAJAJYVCJJWABSRAAWJSCAECQACWSZCASAJSPZDYSCJBJDJBCQCAQYPSDAWAAAD..
3-3 MixinG ..ACAAQDDDAEACAAADYAACCVCAABAABSWDAAAEAAABABVCBVWAEJBPAWYYCAASYSCWAACAAPAVPZCBSCBAPVJBADCAVSAADAAPAAB..
3-4 MixinG ..PPBAEAADCASEEAJWZACASYPADJJADABYDAAAAAJAJYYCJJAABSRACWJSBAECQAEWSZDASAJSQZDYPCJBJDVBCQAAQYPSLAWAVAD..
3-5 MixinG ..ACASQDDBAEACAEADYVACCVCCABADBSWDAAAEASABABVJBVWBEJBPAQYYCDASYSCEAACSAPAVPACBSSBAPVJYADCSVSAADWAPAJB..


Conformation Structure Search
Homology structure search according each PFSC string from PFVM 

PFVM-01:	..APAAQADDAAAEAAAWYAAACYCAAJAABAWYAAAAAAAAAYVCBJWAESBAAWYSCAACYACWAZCAAAASPZCYSCBBPDJBAQCAVYASDAAAAAB..
   1. WYADAADAAAAAADAAADAAAAAAAAAAAAAADAAAADAAAAAAAAAADDDJVAJVAAAAAAAAAAAAAAAAADAADPSWSVAAAAADAPCYAAAAAAA : 6IVP : E :   55 :  153 : 0.601
   2. AAAAADJVAAADDDAADAAAAAAJVAAAAAAAAAAAAAADDAAAAAAAAAAAAAADADJVAAPSWCYDAAAADAAAAAAAAAAPCYAAAADAAAAAAAA : 4C0O : B :  498 :  596 : 0.596
   3. BVAADAAAADAAAAAAAAAAAAAAAAAAAAAADAAAAAAAAAAAAAAAAAAAAAADAAAAAAAAAAAAAAAAAAAAPCSBVAAAAADDAAAAAAAAAAA : 6GY7 : A :  247 :  345 : 0.596
   4. VAAAAADAADAAAAAAJVAAAADAADAAAAAADDAAAAAAAAAAAAAAAADJVAAPSWCYAAAAADAAAAAAAAAAPCYAAAADAAAADAAADAADAAA : 4C0O : A :  498 :  596 : 0.596


CouPlinG: 	..APCSEEWDAAAAAAAPYAAAJVJVAJWADAPYAAAAAAAAAAAJBVDAAJBBVQYJBVAAPSBWAAJWAAAAPZJBEEBBBVJBWQCSVPCSLCCRVAD..
   1. EEBVPSBEWCYAAAAAAAAAAAAAADAAAAAAAAAAAAAAAADAAAAADAAAAAAPYPCZAAAADDAAAAAAADPSBVJEEBBWYPCCSBWCSWCCZAA : 6PU2 : A :  276 :  374 : 0.596
   2. DDAAAAAAAAAAAAAADAAAAAAAADAADDAAADAAAAAAAJVDAAAAAAAAAAAAAJBVAAAAAAAAAAAAAAAAAADJBVJBBBVPCSVPCSVPS-- : 2M1N : A :    9 :  107 : 0.591
   3. EEBVPSBEWCYAAAAAAAAAAAAAAAAAADAAAAAAAAAAAAAAAAAAAAAAAAAPYPCYADAADDAAADAAAAPSBVJEEBVPCYPSBBWCSWCCZDA : 6PTX : B :  276 :  374 : 0.591
   4. AADAAAAJWYPYAAAAADAAAQSBEBWZAAAAAAAAAAAADAAAADAAAAADAAADAAAAAAAJBBVJBEBEEEEUPYADDAJVAAADAAAAAAAAAJV : 6E0I : A :  354 :  452 : 0.586
   5. BVJEEELSBBW$YPSWCREBEEBVPSEEWCYAAAAAAAAAAAAAAAAAAAAAAAAAAADAAAAAAAAAAAAAAAAAJVJVAAAADDAAAAAAAAPSBVJ : 6G1Z : A :  279 :  377 : 0.586
   6. EEBVPSBEWCYAAAAAAAAAAAAAAAAAADDAAAAAAAAAAAAAADAAAAAAAAAPYPCZAAAAADAADAAAAAPSBVJEEBBWYPCSBBWYJWCCYAA : 6PTQ : A :  276 :  374 : 0.586
   7. EEBVPSBEWCYAAAAAAAAAAAAAAAAAADAAAAAAAAAAAAAAAAAAAAAAAAAPYPCZAAAADDJVAAAAAAPSBVJEEBBWYPCSBBWCSWCCZAA : 6PTX : A :  276 :  374 : 0.586
   8. AAAAAAAADAAAAAAAPCYAPYAAAAAAAAAAAAAAADAAAAAAAAJVAJVJBBVAADAAAADDAAAAAAAAADAAAAQSBBBVJEWZDJEEWCSWYAA : 5MDN : A :  476 :  574 : 0.581
   9. AAAAAAAADAAAAAAAPCYAPYAAAAAADAAAAAAADDAAAAAAAAJVAPYJBBVAADAAAADDAAAAAAAAADAAAAQSBBBVJEWZDJEEWCSWYAA : 5MDN : B :  476 :  574 : 0.581
  10. DPCYAADAAAAAADPSVADDAAAPYAAADAAAAAAAAADAAAAAAAAJVJVAAAAAAAAAAADJVJVPYAAPCCCCYAAAAPSBEWCYAAAAAJEBVJE : 1W36 : E :  676 :  774 : 0.581


2-1 MixinG ..APCAQAWDAWAEWAAWAAADCYJAAJYABVWYBAAAJAAEAYCCBJDAECBAJWYSPAAAYABWAZJAAWASJZCYECBEPDCBAQ$AVPASJAAADAB..
   1. AAAAAAAAAAJWYJVAAAAAAAAAAAAAAAJBBVAAAAAAAAAADDAADAAJBVAJVAAAAAAAAAAAAAAAAAAAPYPCSVAAAAAAAAAAAAAAAAD : 5VCH : B :  124 :  222 : 0.545
   2. -------------AAAAAAAADAAAAJVPYAAAAADADDDAAJVDAPCYADDDAADAAAAJWZAAAAAAADDAAADAADAAAAAJWCYPYDAAADDADA : 4NMC : A :   68 :  166 : 0.540
   3. AAAAAADPCZAAAAAAAAAAADAAAAAAAAPSWCYAAAAAAAAAAAADAADPCYAPCSVADAAAADADAAAAAPSWCCCCYDADDAADDAADDDDAAAD : 5HBJ : B :   27 :  125 : 0.535
   4. EWSVJELRBBEEEEBBVJWCSEEBBVADDDJWSBVAAAAAAAAJWYADADDAAADAAAAAAAADDDAAAJBBBVAAAAAAAPYJBBBWYJBVAAAJ--- : 6U7K : C :  942 :  1040 : 0.535
   5. --VPZADAADAADAAAADAAAAADADAAAAJBWSVAAAJWCYPCCSVJVJVADDAAAADDDAAAAAADAAADDAJWCYADJVPYAAADAAAAAAAAAAA : 2MTC : A :   31 :  129 : 0.530
   6. ADAAAJVAAAAAAADAAAAAAAAQSBVJEEEEWSVAAAJVAAPYPSVPSWCCSVJWYAAAAAAAADADAAAADDPYPSBEEEEBLSVAJVAAAAAAAAA : 1W1I : E :   72 :  170 : 0.530
   7. AADAAAAAAAJWYJVAAAAAAAAAAAAAAAJBBVAAAAAAAADADAAADAAJBVAJVAAAAAAAAAAAAAAAAAAAPYPCSVAAAAAAAAAAAAAAAAA : 5VE8 : B :  124 :  222 : 0.530
   8. VAAADAADAAAADAAAAJWYAAAAAAAADAAADAAADAAAAAAAAAAAAAAAAAAADDDDAAAPYAAADAADAADAAAAAAAAAAAAAAADAAJWCYAJ : 6SP2 : A :  161 :  259 : 0.530
   9. VAAADAADAAAADAAAAJWYAAAAAAAADAAADAAADAAAAAAAAAAAAAAAAAAADDDDAAAPYAAADAADAADAAAAAAAAAAAAAAADAAJWCYAJ : 6SP2 : B :  161 :  259 : 0.530
  10. VAAADAADAAAADAAAAJWYAAAAAAAADAAADAAADAAAAAAAAAAAAAAAAAAADDDDAAAPYAAADAADAADAAAAAAAAAAAAAAADAAJWCYAJ : 6SP2 : C :  161 :  259 : 0.530


2-2 MixinG ..JPAADADDDADEAABWYADAYYCABJAAYAPYAADAAADAVYVCCJWAWSVAAWCSCABCPACWJZCAPABSPZJYSCEBBDJBWQCAJYCSDACAAAW..
   1. DQYAJWSWSVAADAAADAAAAAAAQYAJVPYAAAAAAAAAPYAADAAADJVAAPSWCYAJVAJWCSVAAAAADAADDAAAAAAPYAJEBBVDJBBELRW : 6QV4 : A :  943 :  1041 : 0.535
   2. AADAQYAJWRWSVAADAAADAAAAAAAQYAJVPYAAAAADAAAPYAADAAADJVAAPSBWYAJVAJVPSVAAAAAAAADAAADAAAQYAPRBBVDJBBE : 6QV3 : A :  943 :  1041 : 0.530
   3. ADADAADAAADAAAAAPCYAAAAAAAAAAAAAAAAAAAAAAAQSWCCSVAAJVDAJBVAAAAAAADDDDAAADAADAAADJVAAAAAAAAAAAADAAAA : 1Y2H : B :  384 :  482 : 0.525
   4. YAAAAADDADDAPCYAJVAADAAAAAAAAAAAAAAAAAADAAADPCZJWSVAAADDJWCCZAAAAAADAAAAAAADAAAADAADAJVPYAAAAADDADA : 6RD8 : 1 :  378 :  476 : 0.525
   5. ADAAAAAADADAAAAAPCYADAAAAAAAAAADAAAAAAAAAAQSWCCCYAAJVDAJBVDAAAAADDDDDAAADAADAAADJVAAAAADAAAAAAAAAAA : 6F6U : B :  476 :  574 : 0.520
   6. AAAAAJVAADDAADAAAAAAADAAAADA---------AAAAADAAAAAAAAAAADAAAAJVDAAAAAADADAAADAAADDDAAAAAAAADAAADAADDJ : 6E1P : A :  529 :  627 : 0.520
   7. AAAAAADAAADAAAAAPCYDDAAAAAAAAAADAAADAAAADAPSWSBBVAAJVAAJWYDADAADADDDDAAADAADAAADJVAAADDDAAAAAAAAAAA : 3DYQ : A :  394 :  492 : 0.520
   8. AAAAAAAADAAAAAAAAAAAAAPYPSVAAAAAAAAAAAAADAAAAAAAAAPSVAJVAAAADAAADAAAAAAAAPSVAPSVADJVJVJVDAPYPYAPYAA : 6D03 : A :  615 :  713 : 0.520
   9. AAAADAAAAJVAAAAAAAAAAAAAADJBEEWYQSWSBBVPYAAAAADAAAADAAADAAAAAPCYADAAAAAAAAAADAAAAAAAAAAQSWCCCYAAJVA : 3G45 : A :  556 :  654 : 0.520
  10. AAAAAAAADDAAAAADDDDAAADAJBVAAAAAPSVADAAAAAAAAPYJBEWYAAAAADAADAAADAAPYJBVDJWSWYAAQSBVAJVPCYAAAADAAAA : 1UBU : L :  115 :  213 : 0.520


2-3 MixinG ..AJAAQEDDAWAAAAAPYAADCCCAAAAABVWAAAADAAAEAZVCBAWAECBDAWYJCAAAYBCWA$CAAWAAPZCPSCBEPSJBAWCAVPAVDAARAAB..
   1. DDAAAADAAAAAAAAAAAAAADJVAAAAAAADDAAAAAAAAAPSVADAAAAAADJVAAAADAAAAAAAAAAAAAADAAJBBVADAAADDAAAAADAAJV : 6U60 : A :  199 :  297 : 0.571
   2. ----------------------------------------------------------DAADAADAJVAAAAAADAAAAAAAAAAAAAAAAAA------ : 6D3S : A :  1045 :  1143 : 0.566
   3. AAAPYADDDDDAAADAAAADAQCCYADAAAAADAAAADJVAJEEWZAADAAAADADJVAAAAAAAAPSVADDDAPYPCCYJLCSWSVADAAAAAAAAAA : 3FUN : A :  378 :  476 : 0.566
   4. AAAPYADDDDDAAAAAAAADAQCCYADAAAAADAADADJVAJEEWZAADAAAADADJVAAAAAAAAPSVADDDAPYPCCYJLCSWSVADAAAAAAAAAA : 3FH7 : A :  378 :  476 : 0.566
   5. AAAAAJVAAAADAAAAAAAAADPYAJBVAJVADAAAAAAAAAADADAAAAAPCYADAAAAAAAAAAAAAAAAAAAAAQSBBWCYAAJVAJVAAAAAAAA : 2OUS : B :  631 :  729 : 0.566
   6. AAAAAAAADDDDAAAAADAAAAPCYADAADAAAAAAAAAAAAAAAAAADAPCSWCZAAAAAAAAAAAAAAAAAAADAAAJVAJVAAADAAADADAAAAA : 1TJF : A :   53 :  151 : 0.566
   7. WYAAAADAAAAAAAADAPCYADAAAAAAAAAAAAAAAAAAADAAADDJVAPCYAAAAAAAAAADAAAAAAADAAAAAADAJVPYAAAAAAAAAAAAAAA : 3HLX : A :  120 :  218 : 0.566
   8. WYAAAADAAAAAAAADAPCYADAAAAAAAAAAAAAAAAAAADAAADDJVAPCYAAAAAAAAAADAAAAAAADAAADADDAJVPYAAAAAAAAAAAAAAA : 4NY7 : A :  120 :  218 : 0.566
   9. --AAAAAAAAAAAAADAAAAADPSBVAAAAAAAAAAAAAAAAPCYAAAAADDAAAAAAAAAPSWCYADAAAAAAADDAAAADPSBVAAAAAAAAAAAAA : 3TXN : A :   40 :  138 : 0.561
  10. AAAPYADDDDDAAADAAAADAQCCYADAAAAADAADADJVAJEEWYAADAAAADDDJVAAAAAAAAJBVADDDAPYPCCYJLCSWSVADAAAAAAAAAA : 6END : A :  378 :  476 : 0.561


2-4 MixinG ..JPCADAWDDADEWABWAADAYYJABJYAYAPYBADAJADAVYCCCJDAWSVAJWCSPABCPABWJZJAPABSJZJYECEBBDCBWQ$AJYCSJACADAW..
   1. BWZAAAAAAAAAAADAADAAAAADAAAAAAAAAJVAAAAAAADQCCYDDAAAAAADAAAAAAJVJEWZAAAAAAADAAAAAAAQSBWYAAAAAAAAAAD : 1OR6 : A :   45 :  143 : 0.495
   2. DQYAJWSWSVAADAAADAAAAAAAQYAJVPYAAAAAAAAAPYAADAAADJVAAPSWCYAJVAJWCSVAAAAADAADDAAAAAAPYAJEBBVDJBBELRW : 6QV4 : A :  943 :  1041 : 0.495
   3. AAAJVAADAAAAAAAAAAAAAAAAAAAQYPSVAJBVAJVAAAAPCCYADDAJVAJVJVADAAAAJVAAAAJVAAPSBLSWSBBW$SWCZAAAAAAAAAD : 3J32 : B :  3104 :  3202 : 0.490
   4. AAAAAAAAAAAJBVAAAAAAAAAAAJBBVAAAPYPYAAAAAAJVPSBEBEWSVAJVDJBVAAAAADAAAAAAQSWSEEEEEEEWYJVDAAADAJVADAA : 3I1I : B :  263 :  361 : 0.490
   5. PCZAAAADAADAAAAAAAAAAAAAAAPCYAAAAADADAAAAAADPCZAAAAAAAAAQSWCZAAAAAAAAAAAAAADPYPYPSVQCSWREWSVPSVAAAA : 3FVY : A :   28 :  126 : 0.490
   6. AAAAAAAAAAAAAAAAJWYAAAAAAAAAAAAAAAAAAAAAAAPYPSVAAAADAAAAAAAPSBVAJWSBVAJVPSWSVAAPSBBLCYADDAAAAAADAAD : 4A13 : C :  401 :  499 : 0.485
   7. AAAAAADDDDAADADAAAAAAAAAAAAPYAAAAAAAAAAADAAAPCYJEWYADDDDAJVAAAAADAADAADJBVJVJEEWCSVPSBBW$YJEWCYAPSB : 4G3J : B :  284 :  382 : 0.485
   8. AAAAAAAPSVAADDDAAAADAAAAAAAAAAAAAAAAAADDDDJBVJVJWSWCYAJWCSVJBVAJBVAAAAAAAJBVJVAAADAPSBVAAAAAAAAAAAA : 4IZM : A :   67 :  165 : 0.485
   9. AADAQYAJWRWSVAADAAADAAAAAAAQYAJVPYAAAAADAAAPYAADAAADJVAAPSBWYAJVAJVPSVAAAAAAAADAAADAAAQYAPRBBVDJBBE : 6QV3 : A :  943 :  1041 : 0.485
  10. VAAAAADADDAADDDAADAAAAAAAAAAAAAAAAAAAAAADAAAADAAAJWZAAADAAAAAAAJBVJBWYAAJVDAAAAAADAAAAADDAAAADAAAAA : 6EL1 : O :  147 :  245 : 0.485


2-5 MixinG ..AJABQEDAAWAAARAPYDADCCCVAAAJBVWAADADAYAEAZVBBAWWECBDAEYJCVAAYBCPA$CYAWAAP$CPSABEPSJVAWCQVPAVDCARADB..
   1. DDAJVAAAAAAAAAAAAAADAADDJVAAAAAAAAAAAAAAAAAADPSBVAAADDAAAAAAAAAAQYAPCYAAAAAAAADAAAPSBVADAAAAAADAAAA : 5GMK : Z :   65 :  163 : 0.515
   2. ----------------------------------------------------------DAADAADAJVAAAAAADAAAAAAAAAAAAAAAAAA------ : 6D3S : A :  1045 :  1143 : 0.510
   3. AAAAAJVAAAADAAAAAAAAADPYAJBVAJVADAAAAAAAAAADADAAAAAPCYADAAAAAAAAAAAAAAAAAAAAAQSBBWCYAAJVAJVAAAAAAAA : 2OUS : B :  631 :  729 : 0.510
   4. YDADAAAADDADAAAJVPYAADDDAAAAAPSVDAAAAAAAAAAAAAADJVPCSWYJVAJVAAAAAAADAAAAAAAQYPSVAADDADAJWCYPYAAAADD : 6RE2 : 4 :  103 :  201 : 0.510
   5. YDADAAAADDADAAAJVPYAADDDAAAAAPSVDAAAAAAAAAAAAAADJVPCSWYJVAJVAAAAAAADAAAAAAAQYPSVAADDADAJWCYPYAAAADD : 6RE3 : 4 :  103 :  201 : 0.510
   6. ADAADDAAAAA---------------AAAAAAAADAADAAAAADAAAPRBBVAPCSWCCYADAAAAADAAAAJBVAAAAADDAJVAPSVAAPCSVAAPS : 5DLQ : B :  575 :  673 : 0.505
   7. AAAAAJVAAAADAAAAAAAAADPYAJBVAJVAAAAAAAAAAAADADAAAAAPCZADAAAAAAAAAADAAAAAAAAAAQYPSWCYAAJVAJVAAADAAAA : 4HF4 : A :  621 :  719 : 0.505
   8. AAAADADAAAAAAAAAAAAAAAAAAAPYADDAPCYAAAAAAAAAAADAAAAAJVDAJWSWCYAAAAAAAAADDDAAAAJVAAAAADAAAADADAAAAAJ : 2DGJ : A :  130 :  228 : 0.505
   9. AAAAAADAAAAAAAAPYAAAAPCYAAAAAAAADAADAAAAAAAAAADQYAPCYDAAAAAAAAAAAPSWCYADAAAAAAAAAAAAAJBWCZAAADAAAAA : 5Q0R : A :  296 :  394 : 0.505
  10. AAAJVJVADAAAAAADAAAAAAPCYAAAAADAAPYAADDAAAAAAJVJVADAADAAADDAAAAAPCZADAAADAAQSVAAAAAAAAAJVJVAAADAAAA : 3TJ3 : B :  264 :  362 : 0.505


3-1 MixinG ..APBAQAADAEAEEAAWZAACCYPAAJJABSWYDAAAAAABAYYCBJAAEJBACWYSBAASYAEWAZDAAPASQZCYPCBAPDVBAQAAVSASLAAAVAB..
   1. ADAAAAAAAAAAAAAAAAAAAPCYAAAAAJVAAAAAAAAAAAAAAAAAAAADJVPSWSBVAAAAAAAADAAPYAAAAAAAAAAAAAAAAAAAAAAAAAA : 6C9A : B :  144 :  242 : 0.545
   2. ADAAAAAAAAAAAAAAAAAAAPCYAAAAAJVAAAAAAAAAAAAAAAAAAAADJVPSWSBVAAAAAAAADAAPYAAAAAAAAAAAAAAAAAAAAAAAAAA : 6C9A : A :  144 :  242 : 0.545
   3. AAAAADAAAAAAAADAPCZAAAAAAAAAAAAADAAAAAAAADAAAAAAAQYJBVJWYAAAAAAAAAAADAADAAAQYAPSVAPSVAAAAAAAAAAADDA : 2Q24 : B :   70 :  168 : 0.545
   4. QYJVAAAAADDAADJWYAAAAADAAADDDDDDDDAAAAAAAAAAA------------------------------------------------------ : 6NT3 : A :  893 :  991 : 0.545
   5. -------SWCYAADAAAJVAJVAAAADAADAADDDAAAAAAAADAAAAADAAAPYPSVAAAADAAAAAAAAADAAADAAADAAAADDADAAAAAAAAAA : 6S7P : A :  779 :  877 : 0.540
   6. AAAPSBWZAAAAAAAAADAAAAAAAAAJWYJEWYAAAADADAAAAADAAADDAAJVPSVAAAADDDADDAAAADDDQCSWSVADDAADAAAAAAAAAAA : 2N3E : A :   29 :  127 : 0.540
   7. AAAAADAJVPYADAADAADAAAAAADAJVAAAAAJVADAAAAAAAAAJ--------AADDAAAAAAAAAAADJVAAAAAAAAAAAAAAAADAAJVAJBW : 5ZCS : A :  1013 :  1111 : 0.540
   8. AAAAADAJVPYADAADAADAAAAAADAJVAAAAAJVADAAAAAAAAAJ--------AADDAAAAAAAAAAADJVAAAAAAAAAAAAAAAADAAJVAJBW : 5ZCS : B :  1013 :  1111 : 0.540
   9. AAAJWYAJBVAAAAAAAAAAAJBVAAPCYAAAAAAAAAAAAAAAAAAAJVAAAAAAAAAAAAAAAPYAAADAADDAAAAAADJVAAAAAAAAAAAAADA : 3ZKV : A :  546 :  644 : 0.540
  10. EBVPYADAAAAAADAAAAAAAAAAAAAAAAAADAAAAAAAAAADAAAAAAAAAAAAAADAAAAAADAADAAAPYAPCYAAAAAAAAAAAAAAAAAAAAA : 6IOK : C :  128 :  226 : 0.540


3-2 MixinG ..PPAAEADDCASEAAJWYACASYCADJAADABYAAAAAAJAJYVCJJWABSRAAWJSCAECQACWSZCASAJSPZDYSCJBJDJBCQCAQYPSDAWAAAD..
   1. AAAAAAJVJVAAAAAAADAAADAADQSVADAAAAAADAAADAAAAJBVAJVDDAAAADAAAAAAAADJWZAAAAAAADAAAAAAAAAAJVJWCYAAAAD : 2QMR : B :  742 :  840 : 0.520
   2. PYAAAAAAAAAAJVJVAAAAAAADAAADAADQSVADAAAAAADAAAAAAAAJBVAJVDDAAAADAAAAAAAADJWZAAAAAAADAAAAAAAAAAJVJWC : 2QMR : C :  742 :  840 : 0.520
   3. JWYAJVAAAAAAADAADAAAAJWZAAAAAAAAAAAAAAAAAAADAAAADAAADJVDAPREWSVAAAAAAAADADAAAADJWZAAAAAAAAPYAAAAAAA : 5YVG : B :  742 :  840 : 0.515
   4. ------PZDAPCYAAAAJVAAJWZDDAAADAAAAAAAJWYAAAAAAAAAAAAAAAAAAAADAAJWYJWSVADDDAAAAAAAAAAADPSWCYAAADADAA : 6FSH : B :   93 :  191 : 0.510
   5. AAAAAAAADDDJBBVAADAAAAAAAADAAAAPYAAAAAAAJVAAAAAAAAAAAAAAJBWYDDAAAAAAAAAAAAADDDDAJBVAJVAAAAAAAAAAAAA : 5NW5 : B :  505 :  603 : 0.510
   6. AAAAAADAAADAAAAAPCYADAAAAAAAAADDAAAAAAAAAAQSWCCSVAAJVDAJBVAAAADAADDDDAAADAADDAADJVAAAAAAAAAAAAAAAAA : 3D3P : A :  384 :  482 : 0.505
   7. ADPCZAAAADAPFCCYAADDPSLSBVDPSBEWCSVAAAAAAADDDJWYAAAAAPYAAAADAADDAAAAAAAADAAAAJVADAAAAAAAAJWCSBVAADD : 6H2J : B :  753 :  851 : 0.505
   8. AAADAAAAADDQSVADAAAAAADAAAAAAAAJBVAJVDDAADADAAAAAAADDJWZAAAAAAADAAAAAADAAAJVJWCYAAAAAAAAAAAAAADDJVA : 2QMR : A :  742 :  840 : 0.505
   9. AAAAAADAAADAJVAAAAPCCYAAAAAAAADAAAAAAAAAAAAAAAAAAADAAAADAAAAAAAAAPCZAAAAAAPCSWCCSVAAPSWYAAADAADAAAA : 2OEX : A :  462 :  560 : 0.505
  10. AAAAAAAJWSWCCSVPCYAAAAAAAADAAAAAAAADAAAADAADDJVAAAAAADADAAAAAAAAAADAAAAAAAJVJVADAAAAAAAAAADAAAAAAAA : 6R9T : A :  1926 :  2024 : 0.505


3-3 MixinG ..ACAAQDDDAEACAAADYAACCVCAABAABSWDAAAEAAABABVCBVWAEJBPAWYYCAASYSCWAACAAPAVPZCBSCBAPVJBADCAVSAADAAPAAB..
  1. AAAAAAADAAAAAAAAAAAAPCYADAAAAAAAAAAAAAAADDAJVJBVJVAAPYADAAAAAAAAAAAAAAAADAAAAAJVDAAAJVAAAAAAAAAAAAA : 1ZB1 : B :  120 :  218 : 0.556
   2. VAAAAAAAAAAAAADDAAAAAAAAAAAAAAAAAADDJBBVAAAAAAAAAAAAAAADAAAAAADAADAJWCYJVJVPYAJVAQCSBVQSVAAJVAAAAAD : 6HZG : A :  612 :  710 : 0.556
   3. ADAAAAADAPSWCYAAAAAAAAAAAAAAAJBBWZAAADAAAAAAAJBVAPYJBVJVAAAAAAAAAAAAAAAAAAADDJVAJVAAAAADAAAAAAAAAAA : 3OMK : A :  352 :  450 : 0.551
   4. AAADADAAAAADADAAAAAAJVJVDAAAAAAAAAAAAAAAAAAAPYJVAAAAAAADAAAAAAADADAAAAAAAAAQCSBVJVAAJBBWYADJVAAAAAA : 5LC5 : L :  277 :  375 : 0.551
   5. AAAAAAADAPSWCYAAAAAAAADAAAAAAJBBWZAAADAAAAAAAJBVAPYJBVJVAAAAAAAAAAAAAAAAAADDDJVAJVAAAAADAAAAAAAAAAA : 5Q1F : A :  352 :  450 : 0.551
   6. AAADAAADAPSWCYAAAAAAAADAAAAAAJBBWZAAADAAAAAAAJBVAPYJBVJVAAAAADAAAAAAAAAAAAADDJVAJVAAAAADAAAAAAAAAAA : 5Q1H : E :  352 :  450 : 0.551
   7. AAAAAAAAAAAAAAAAAAAAAAAADADDAAPSVAAAAAAAAAAAAJBBVAAAADAAAAAAAAAAADAAAAAAAAAAJVJVAAAAAAAADAAAADDAAAA : 5XFL : A :  365 :  463 : 0.551
   8. VAAAAAADADAADAAAADDAAADDDJBBVAJBVDAAAAAAAAPSWCYADADAAAAAAAAAAAJBWCZAAADAAAAAAAJBVAPYJBVJVAAAAADAAAA : 3RUU : A :  315 :  413 : 0.551
   9. WYADAADAAAAAADAAADAAAAAAAAAAAAAADAAAADAAAAAAAAAADDDJVAJVAAAAAAAAAAAAAAAAADAADPSWSVAAAAADAPCYAAAAAAA : 6IVP : E :   55 :  153 : 0.551
  10. ----VADADAAADAAAAQYAJVPYAAADAJBBVDAAAADAAAAJVPYADAAAAAAAAAAAAAAAAAAAAAAAAAAAAAAAAAAAAAAAAAADAAAAAAA : 1EZJ : A :    2 :  100 : 0.545


3-4 MixinG ..PPBAEAADCASEEAJWZACASYPADJJADABYDAAAAAJAJYYCJJAABSRACWJSBAECQAEWSZDASAJSQZDYPCJBJDVBCQAAQYPSLAWAVAD..
   1. DAAQSVAAAAAAADDAJWYJVADAADAAAAAAAAAAAPYAAAAAAAAAAAQSVADAAAAAAPYAJWYADAAAAAAAAAAAAAJVAAADAAAAQSVADAA : 1I7W : C :  440 :  538 : 0.500
   2. AAAQSVADADAAAADAJWYJVADAADAAAAAAAAAAAPYAAAAAAAADAAQSVADDAAAAAPYAJWYDDADDAAAAAAAAAAJVAAADAAAAQSVAAAA : 1I7X : C :  440 :  538 : 0.495
   3. AAAQSVAAAAAAAAAAJWYJVADAADAAAAAAAAAAAPYAAAAAAAAAAAQSVAAAAAAAAPYAJWYADAAAAAAAAAAAAAJVAAADAAAAQSVADAD : 2Z6H : A :  440 :  538 : 0.495
   4. DAAQSVAAADAAAADAJWYJVADAADDAAAAAAAAAAPYAAAADDAAAAAQSVAADAAAAAPYAJWYADADAAAAAADAAAAJVAAADAAAAQSVADDA : 1JPW : A :  440 :  538 : 0.495
   5. AAAQSVAAAAAAAAAAJWYJVADAAAAAAAAAAAAAAPYAAAAAAAAAAAQYAAAAAAAAAPYAJWYAAAAAAAAAAAAAAAJVAAAAAAAAQSVADAA : 1JPP : A :  440 :  538 : 0.490
   6. AAAQSVAAAAAAAAAAJWYJVADAAAAAAAAAAAAAAPYAAAAAAAAAAAQYAAAAAAAAAPYAJWYAAAAAAAAAAAAAAAJVAAAAAAAAQSVADAA : 1JPP : B :  440 :  538 : 0.490
   7. AAAQSVAAADAAAAAAJVAJVADAAAAAADAAAAAAAPYAAAAAAAAAAAQSVAAAAAAAAPYAJWYADAAAAAAAAAAAAAJVAAADDAAAQSVADAA : 3OUW : A :  440 :  538 : 0.490
   8. AAAQSVAAAAAAAAAAJWYJVADAADAAAAAAAAAAAPYAAAAAAAAADAQSVAAAAAAAAPYAJWYADAAAAAAAAAAAAAJVAAADAAAAQSVAAAA : 1QZ7 : A :  440 :  538 : 0.490
   9. AAAAAAAAADPYJBVAAADDAPCYAAAAJVDDAAAAAAAAAAAAAAAAAAJBVAAAPRBEEEEWZDAADDAADAQYAPCYAAAAAAADAAADAAAAAAA : 2QJ3 : B :   29 :  127 : 0.490
  10. VAAAAAAAAAAAAAAADDAADAAAAAAAAAAAADAAADAAAAAADDAADAAADAAAJWZQYJWCSVAAAAAAAAAAADAPSVADAADJVJBVAPYADDA : 1W27 : B :  503 :  601 : 0.490


3-5 MixinG ..ACASQDDBAEACAEADYVACCVCCABADBSWDAAAEASABABVJBVWBEJBPAQYYCDASYSCEAACSAPAVPACBSSBAPVJYADCSVSAADWAPAJB..
   1. AAAAPSVDAAAAADAAAAAAAAAPCYAAAAJVADAAAAAAADAJWSBVAJVJVAAAAAAAAAADAADAAAAAAAADDJWZAAAAAAAAAAAAAAAAAAD : 3UP3 : A :  561 :  659 : 0.485
   2. AAAAAAADAAAAAAAAAAAAPCYADAAAAAAAAAAAAAAADDAJVJBVJVAAPYADAAAAAAAAAAAAAAAADAAAAAJVDAAAJVAAAAAAAAAAAAA : 1ZB1 : B :  120 :  218 : 0.480
   3. AAAAPSVDAAAAAAAAAAAAAAAPCYAAAAJVADAAAAAAAAAAPSBVAJVJVAAAAAAAAAADAAAAAADAAAADDJWZAAAAAAAAAAAAAAAAAAA : 3UP0 : A :  561 :  659 : 0.480
   4. AAADADADDAADDDDDAAAAAAPYPSVAAAAADAAAAAAAADDJVJVAAAADDAAAAAAAQSWCYAAAADAADAAADAJBVAAAAAADDAAAAADAAQS : 3V8F : A :  234 :  332 : 0.480
   5. AAADADADDAADDDDDAAAAAAPYPSVAAAAADAAAAAAAADDJVJVAAAADDAAAAAAAQSWCYAAAADAADAAADAJBVAAAAAADDAAAAADAAQS : 3V8F : B :  234 :  332 : 0.480
   6. AAAAAAADAAAAADDADADADAAAADAAAPCCCYJBVDAAAAAAAAAAAAAJVJVADDAADAAAAAAAAAAAAAAAAAAAAAAADAAAAAADAAAADAA : 4WIC : A :  215 :  313 : 0.480
   7. JVAAAAAAADDAAAAAAAAAADDDAAAAAAPSWCCZAADDAAAJVJBBWSLSVAPYAAAAAAAAADAAPSVAJVPCFRWSVJEBBBEWCSVAAAAAAAA : 1WOK : D :  756 :  854 : 0.480
   8. --------------------JWZAAADAAAAAAJVAAAADADAAAAAAAAJVAADDDJVAAAAAAAAAAAAAAAAAAAPCZAAAAAAAAAAAAAAAAAA : 4UWG : A :  503 :  601 : 0.475
   9. AAAAAAAAAAAADDADDAAADAJVAAADDPCCSVAADJBWSWYADAAAAAPYPSBVAAAAAAAAAAAPCCCCZADDAAAAAADADJBVAAAAAAAAAAA : 5ERO : B :  599 :  697 : 0.475
  10. AAAAAAADDAAAAAAAADJVAADAQYAADAAAAAAAADDDAAAAAAAAPCZJVDAAAAADDADAADDJBVAAAAPCSBVJBVJVJVAAPSWCYAJVAAA : 6OIV : F :   75 :  173 : 0.475
